# Novel green synthesis of polyfunctionally substituted phthalazines promoted by visible light, DFT studies and molecular docking with antimicrobial and antibiofilm potency

**DOI:** 10.1038/s41598-026-47154-w

**Published:** 2026-05-05

**Authors:** Ramadan A. Mekheimer, Basma A. Khalifa, Zeinab Shawky Hashem, Samar M. R. Allam, Kamal Usef Sadek, Mohamed R. Eletmany

**Affiliations:** 1 Chemistry Department, Faculty of Science, Minia University, Minia, 61519 Egypt; 2 Botany and Microbiology Department, Faculty of Science, Minia University, Minia, 61519 Egypt; 3 Microbiology and Immunology Department, Faculty of Pharmacy, Minia University, Minia, Egypt; 4 Chemistry Department, Faculty of Science, Qena University, Qena, 83523 Egypt

**Keywords:** Chemistry, Drug discovery

## Abstract

Visible-Light-Mediated synthesis is a green and efficient technique that has garnered interest from organic chemists. A new series of polyfunctionally substituted phthalazine derivatives **3a–j** were synthesized *via* a metal-free reaction of pyridazines **1a–d** with arylidenes** 2a–d** in the presence of a catalytic amount of piperidine, in absolute ethanol as a solvent, under white LED lamp irradiation at room temperature using open air as oxidant, in 90–93% yields. A radical mechanism *via* single-electron transfer (SET) was confirmed using a radical scavenger. All synthesized products **3a–j** were screened as potential antimicrobial and antibiofilm agents. Both compounds** 3g** and** 3j** are the most promising derivatives. Compound** 3g** displayed outstanding activity against *Pseudomonas aeruginosa* and *Klebsiella pneumoniae* with a MIC value of 3.12 and 12.5 µg/mL, respectively. Compound** 3j** gave a Minimum Inhibitory Concentration (MIC) value of 6.25 µg/mL for *P. aeruginosa*. As prospective antibiofilm agents against *P. aeruginosa*, compounds** 3g** and** 3j** exhibited inhibition with 81% and 75%, respectively. The geometrical structures were optimized using Density Functional Theory (DFT), frontier molecular orbitals (HOMO–LUMO), along with global reactivity descriptors, such as chemical potential, electronegativity and electrophilicity, were computed to assess the compounds’ chemical reactivity and stability. Spectral characteristics were interpreted to support the theoretical findings, while Molecular Electrostatic Potential (MEP) mapping was employed to visualize charge distribution and potential reactive sites. Furthermore, topological analyses, including Electron Localization Function (ELF) and Reduced Density Gradient/Non-Covalent Interaction (RDG/NCI) analysis, offered insights into intra- and intermolecular interactions and the nature of non-covalent bonding. Molecular docking simulations were performed and revealed favorable binding affinities and key interactions within the active sites, indicating potential antimicrobial and enzyme-inhibitory properties, especially with** 3j**-*3LD6* with binding energy of − 9.5 kcal/mol.

**Supplementary Information:**

The online version contains supplementary material available at 10.1038/s41598-026-47154-w.

## Introduction

**Fig. 1:**
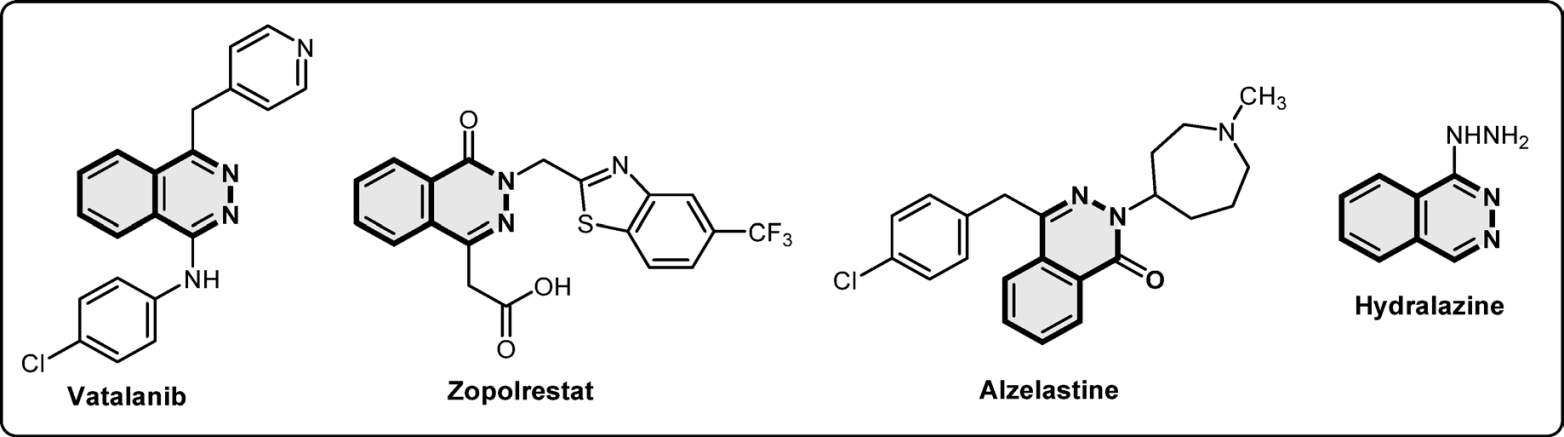
Biologically active phthalazine derivatives.

Phthalazine derivatives are remarkably important nitrogen-containing heterocycles, a fact supported by the high proportion of Food and Drug Administration (FDA) approved drugs^1^. These molecules occupy a unique position in medicinal chemistry owing to their biological activity as antidiabetic^2^, vasorelaxant^3^, antiallergic^4^, PDE4 inhibitors^5^, drug molecules like hydralazine^6,7^, antitumor^8–10^, zopolrestat^11^, anti-asthmatic antimicrobial^12^, antihypertensive^13^ and anti-inflammatory agent^14^. Representative examples identified several key therapeutic areas where phthalazines are prominent illustrated in Fig. 1.

The synthesis of phthalazine scaffolds often involves ring closure reactions between hydrazine or arylhydrazines and dicarbonyl compounds^15,16^, phthalic anhydride^17^, Diels–Alder reaction^4,18^, Ullmann-Goldberg and Buchwald-Hartwig cross coupling or Chan-Evans-Lam coupling^19–22^. Although, these reactions show tremendous improvements, sometimes require harsh reaction conditions; use of toxic reagents and limiting accessibility to the targeted scaffolds^4,23,24^. The transition to visible light mediated reactions represents a paradigm shift in synthetic organic chemistry. Brachet and co-authors^25,26^, and Satyanarayana and Suchand^27^ developed cascade reactions and palladium-catalyzed acylation for phthalazine synthesis promoted by visible light. However, there is a need for looking for efficient, high yielding, and metal-free green strategies that can efficiently produce polyfunctionally substituted phthalazines at ambient temperature.

Antimicrobial therapy is facing a significant problem due to the emergence of multidrug resistance (MDR) in microbial infections as a result of the formation of biofilms. These biofilms are organized communities of bacteria encased in an extracellular polymeric substance (EPS) matrix^28^. The EPS serve as a physical and chemical barrier protecting the bacteria from both antibiotics and the hosts immune response. The resistance is further complicated by quorum sensing (QS), a population-depending signaling mechanism that bacteria use to coordinate biofilm formation and virulence gene expression^29^. Several articles highlight the danger biofilm-related infections on medical devices, particularly those involving *Staphylococcus epidermidis* and *Staphylococcus aureus*^30–32^. Several studies have reported that newly synthesized phthalazine derivatives exhibit significant antibacterial activity against both Gram-positive and Gram-negative bacterial strains at various Minimum Inhibitory Concentrations (MIC)^33–35^.

In continuation to our efforts in the green synthesis of biologically relevant heterocycles^36–40^. we report herein, for the first time, a general, milder and efficient green synthesis of polyfunctionally substituted phthalazine *via *visible light photocatalytic reaction of methylpyridazines with ethyl 2-cyano-3-arylacrylates. Moreover, the biological evaluation reinforces the therapeutic significance of new synthesized phthalazines as promising candidates for antimicrobial and antibiofilm applications.

## Experimental

*General*. Melting points were recorded with an electrothermal melting point apparatus. ^1^H- and ^13^C NMR were run with Bruker DPX instrument (400, 600 MHz) for ^1^H NMR and (100, 150 MHz) for ^13^C NMR spectrometer in DMSO-*d*_6_ as solvent with tetramethyl silane (TMS) an internal standard. Chemical shifts are expressed in *δ* ppm. Mass spectra were measured on a VG Auto spec Q MS 30 and MS 9 (AEI) spectrometer, with electron ionization (70 eV) mode. Microanalyses were performed at the Microanalytical Data Unit, Cairo University. All reactions were monitored by thin layer chromatography (TLC) as aluminum sheets pre-coated Merck Silica gel (60F_254_) until the completion of the reaction. All chemicals were purchased from Aldrich or Merck companies and used without any further purification. The reaction mixture was exposed to visible light in open air using LED lamp (30 ًًً W) at ambient temperature.

*General procedure for the synthesis of phthalazine derivatives 3a–j*. To a mixture of pyridazines **1a–d** (1 mmol) and arylidenes **2a–d** (1 mmol) in abs. ethanol (10 mL), piperidine (30 mol%) was added. The reaction mixture was irradiated with white LED lamp (30W) in open air for 16 h. The reaction progress was monitored by TLC. The resulting solid product was collected by filtration, washed with warm EtOH and dried to afford pure samples of the products **3a–j**.

### Antimicrobial screening

#### Microorganisms, culture conditions, and compound preparation

Antimicrobial testing was performed against clinically multi-resistant isolated strains of *S. aureus*, *K. pneumoniae*, *P. aeruginosa*, and *C. albicans*, obtained from the Microbiology Laboratory, Faculty of Pharmacy, Minia University. Bacterial strains were cultured in tryptic soy broth (TSB), and *C. albicans* was cultured in Sabouraud dextrose broth (SDB). Cultures were incubated at 37 °C for 24 h and adjusted to a 0.5 McFarland standard. A series of synthesized polyfunctionally substituted phthalazine derivatives **3a–j** were evaluated. Each compound was dissolved in dimethyl sulfoxide (DMSO) to prepare 2 mg/mL stock solutions and subsequently filtered using 0.22 μm filters. Working concentrations were prepared by serial dilution in sterile distilled water prior to testing.

#### Antimicrobial susceptibility testing

The agar well diffusion method was performed to assess the antimicrobial activity. Mueller–Hinton agar (MHA) plates for bacteria and Sabouraud dextrose agar (SDA) plates for fungi were inoculated with standardized microbial inocula. Six millimetres wells were punched and filled with 20 μL of each compound. Ciprofloxacin (20 μg/mL) and fluconazole (20 μg/mL) served as reference antibiotics for bacterial and fungal strains, respectively, while DMSO was used as a negative control. The plates were incubated overnight at 37 °C, and inhibition zone diameters were measured.

#### Determination of minimum inhibitory concentration (MIC) and minimum bactericidal/fungicidal concentration (MBC/MFC)

The Minimum Inhibitory Concentration (MIC) values were determined using the broth microdilution method. Two hundred microliters of each phthalazine derivative solutions were added to the first column of a 96-well microtiter plate, followed by serial two-fold dilutions across the row, resulting in final concentrations ranging from 200 to 0.82 μg/mL. Each well received 10 μL of bacterial or fungal inoculum suspension. Columns 11 and 12 served as microbial growth and broth sterility controls respectively. Plates were incubated at 37 °C for 24 h. The MIC was defined as the lowest concentration of an antimicrobial agent that completely inhibits the visible growth of a microorganism. All data were reported as the mean of three independent experiments, each performed in duplicate. The Minimum Bactericidal and Fungicidal Concentrations (MBC and MFC) were determined polystyrene microplates by streaking samples from each MIC well onto agar plates. The lowest concentration at which no visible microbial growth was observed after incubation, indicating bactericidal or fungicidal activity.

#### Biofilm inhibition assay

The antibiofilm activity of the phthalazine compounds was investigated using the crystal violet staining method in 96-well flat-bottom^41^. Each well was loaded with 180 μL of TSB supplemented with 1% glucose, 10 μL of microbial inoculum, and 10 μL of test compound. Negative control wells received an equivalent volume of DMSO. Following static incubation at 37 °C for 24 h, non-adherent cells were removed by washing with phosphate-buffered saline (PBS, pH 7.2). Plates were air-dried and biofilms were fixed with 99% methanol for 15 min. Wells were then stained with 0.5% crystal violet for 30 min, washed with distilled water to remove excess stain, and air-dried. The bound dye was solubilized using 95% ethanol and absorbance was measured at 570 nm using a microplate reader.

The biofilm inhibition percentage was calculated using the following formula:

$$\% {\mathrm{Inhibition}}\, = \,\left[ {\left( {{\text{OD control}}{-}{\text{OD treated}}} \right)/{\text{OD control}}} \right]\, \times \,{1}00$$

All experiments were performed in triplicate and repeated independently three times.

### Computational methodology

#### DFT strategy

Calculations were carried out using the 6-311G(d,p) basis set within the Gaussian 09 software suite^42^. Molecular electrostatic potential (MEP) mapping was performed to pinpoint key nucleophilic and electrophilic regions in the optimized structure. The most stable conformer and its electronic excitation properties were visualized using Chemcraft^43^ and VMD^44^ to elucidate its electronic characteristics. Harmonic vibrational frequencies computed at the 6-311G(d,p) level were scaled using a factor of 0.967, as recommended by NIST (https://cccbdb.nist.gov/vibscalejustx.asp)^45^, to correct for anharmonic effects. ^1^H and ^13^C NMR spectra were computed using the Gauge-Including Atomic Orbital (GIAO) method within the DFT framework^46^. Time-Dependent Density Functional Theory (TD-DFT), a widely adopted approach for simulating UV/vis absorption spectra and characterizing electronic excitation states in various molecular systems^47^, was employed in this study. The TD-DFT calculations were conducted using the Conductor-like Polarizable Continuum Model (CPCM) to simulate solvent effects in DMSO.

Further topological analyses were executed using Multiwfn software^48^, including reduced density gradient (RDG) and non-covalent interaction (NCI) analyses, alongside the electron localization function (ELF), to thoroughly characterize the intramolecular interactions and bonding nature within the heterocyclic framework^49^.

The quantum chemical reactivity parameters for all the designed systems were computed following geometric optimization. These parameters were evaluated using the HOMO and LUMO energy values, based on the equations provided as the following^50,51^.

1 $${\text{Energy Gap }}\left( {\Delta {\mathrm{E}}} \right) \, = {\text{ E}}_{{{\mathrm{LUMO}}}} - {\text{ E}}_{{{\mathrm{HOMO}}}}$$

2 $${\text{Ionization potential }}\left( {\mathrm{I}} \right) = - {\text{ E}}_{{{\mathrm{HOMO}}}}$$

3 $${\text{Electron affinity }}\left( {\mathrm{A}} \right) = - {\text{ E}}_{{{\mathrm{LUMO}}}}$$

4 $${\text{Hardness }}\left( \upeta \right) = \left( {{\mathrm{I}} - {\mathrm{A}}} \right)/{2}$$

5 $${\text{Chemical potential}}\left( \upmu \right) = - \left( {{\mathrm{I}} + {\mathrm{A}}} \right)/{2}$$

6 $${\text{Softness }}\left( \sigma \right) \, = { 1}/ \, \upeta$$

7 $${\text{Electronegativity }}\left( \chi \right) \, = \, - \left( {{\mathrm{E}}_{{{\mathrm{HOMO}}}} + {\text{ E}}_{{{\mathrm{LUMO}}}} } \right)/{2}$$

8 $${\text{Electrophilicity }}\left( \upomega \right) \, = \, \upmu^{{2}} /{2}\upeta$$

#### Molecular docking methodology

The molecular docking procedure followed a well-established protocol to ensure reliable and reproducible results^52,53^. Docking simulations for the investigated heterocyclic compounds were conducted using AutoDock Vina software^54^. Post-docking analysis and visualization of ligand–protein interactions were carried out using Discovery Studio (https://www.3ds.com/products-services/biovia/). The selected receptors S. aureus (ID:2XCT)^55^ and human CYP51 (ID: 3LD6)^56^ were obtained from the Protein Data Bank (https://www.rcsb.org/). Protein preparation involved the removal of water molecules and non-essential atoms, adding polar hydrogen atoms, and assigning partial atomic charges. The ligand and protein files were converted into the PDBQT format for docking. Active site coordinates were determined, and a grid box was defined with dimensions of 40 × 40 × 40 Å and a grid spacing of 0.375 Å. The grid centers were set at coordinates (x = 21.348, y = 24.336, z = 79.243) for 2XCT and (x = 42.348, y = − 0.623, z = − 1.711) for 3LD6. The Genetic Algorithm (GA) was employed as the docking search method to predict optimal binding conformations^57^.

## Results and discussions

### Chemistry

We started our studies through the reaction of equimolar amounts of ethyl 1-(4-chlorophenyl)-5-cyano-4-methyl-6-oxo-1,6-dihydropyridazine-3-carboxylate (**1a**) with ethyl 3-(4-chloro-phenyl)-2-cyanoacrylate (**2a**) in abs. ethanol in the presence of a few drops of piperidine, as a base catalyst, promoted by conventional or microwave heating at 70 °C. The reactants were recovered almost unchanged even after heating for prolonged time. This is in contrast to the previously reported formation of the phthalazines by reaction of methyl-pyridazine **1** with arylidene-malononitriles^58^. This could be explained by the less reactivity of ylidenic double bond in **2** as a result of weaker electron-withdrawing ability of ester function compared to cyano group^59^. Delightly, performing the reaction under visible light in EtOH/pip in oxygen atmosphere for 16 h at ambient temperature afforded the corresponding diethyl 5-amino-7-(4-chlorophenyl)-4-oxo-3-(4-chlorophenyl)-3,4-dihydrophthalazine-1,6-dicarboxylate (**3a**) in 93% yield. Structure of **3a** was established based on spectral and analytical data. Mass spectra of **3a** revealed molecular ion peak [M^+^] at 526 (100%). ^1^H NMR showed two triplets at *δ* = 0.82 and 1.29 ppm (*J* = 7.2 Hz) for two esters CH_3_ groups, two quartets at *δ* = 3.99 and 4.36 ppm (*J* = 7.2 Hz) for two esters CH_2_ groups and NH_2_ function at *δ* = 7.97 ppm appeared, in addition to aromatic protons at *δ* = 7.34, 7.40, 7.56, and 7.60–7.66 ppm. The ^13^C NMR spectrum revealed three distinct carbonyl (C = O) signals at δ 160.34, 162.58, and 166.87 ppm, while the CH₃ and CH₂ groups of the two ester moieties appeared at δ 13.08, 13.91 and 60.94, 61.93 ppm, respectively. We examined the model reaction at 25 °C for 16 h with different solvents and bases and under catalyst free to optimize reaction conditions (Table 1).

**Table 1:** Optimization of reaction conditions for compound **3a**.

Entry	Solvent	Base	(Yield%)
1	DMF	Na_2_CO_3_	25
2	DMF	Et_3_N	30
3	DMF	Piperidine	35
4	CH_3_CN	Na_2_CO_3_	40
5	CH_3_CN	Et_3_N	45
6	CH_3_CN	Piperidine	50
7	THF	Na_2_CO_3_	40
8	THF	Et_3_N	45
9	THF	Piperidine	50
10	Dioxane	Na_2_CO_3_	52
11	Dioxane	Et_3_N	55
12	Dioxane	Piperidine	60
13	EtOH	Na_2_CO_3_	80
14	EtOH	Et_3_N	82
15	**EtOH**	**Piperidine**	**93**
16	EtOH	**–**	**NR**

Signifying the crucial role of the catalyst carrying out the reaction under nitrogen atmosphere which serves as protective shield preventing air oxygen afforded a trace amount of product **3a** implying its necessity for the reaction. The reaction temperature was monitored by inserting thermometer in the reaction mixture which did not exceed 25 °C (room temperature) indicating a photochemical pathway rather than thermal one.

A controlled experiment was conducted to clarify the reaction mechanism. A radical inhibition experiment was performed by adding 1.0 mol% of 1,4-benzoquinone, as a radical scavenger, to the reaction mixture under the same standard reaction conditions. No product was detected after 8 h exposure to visible light indicating the involvement of radical intermediate in the reaction course. It is worth mentioning that in the metal catalyst-free visible light synthesis at least one of the starting materials is able to absorb the light and activate the single electron transfer pathway generating the radical species^60^.

**Scheme 1:**
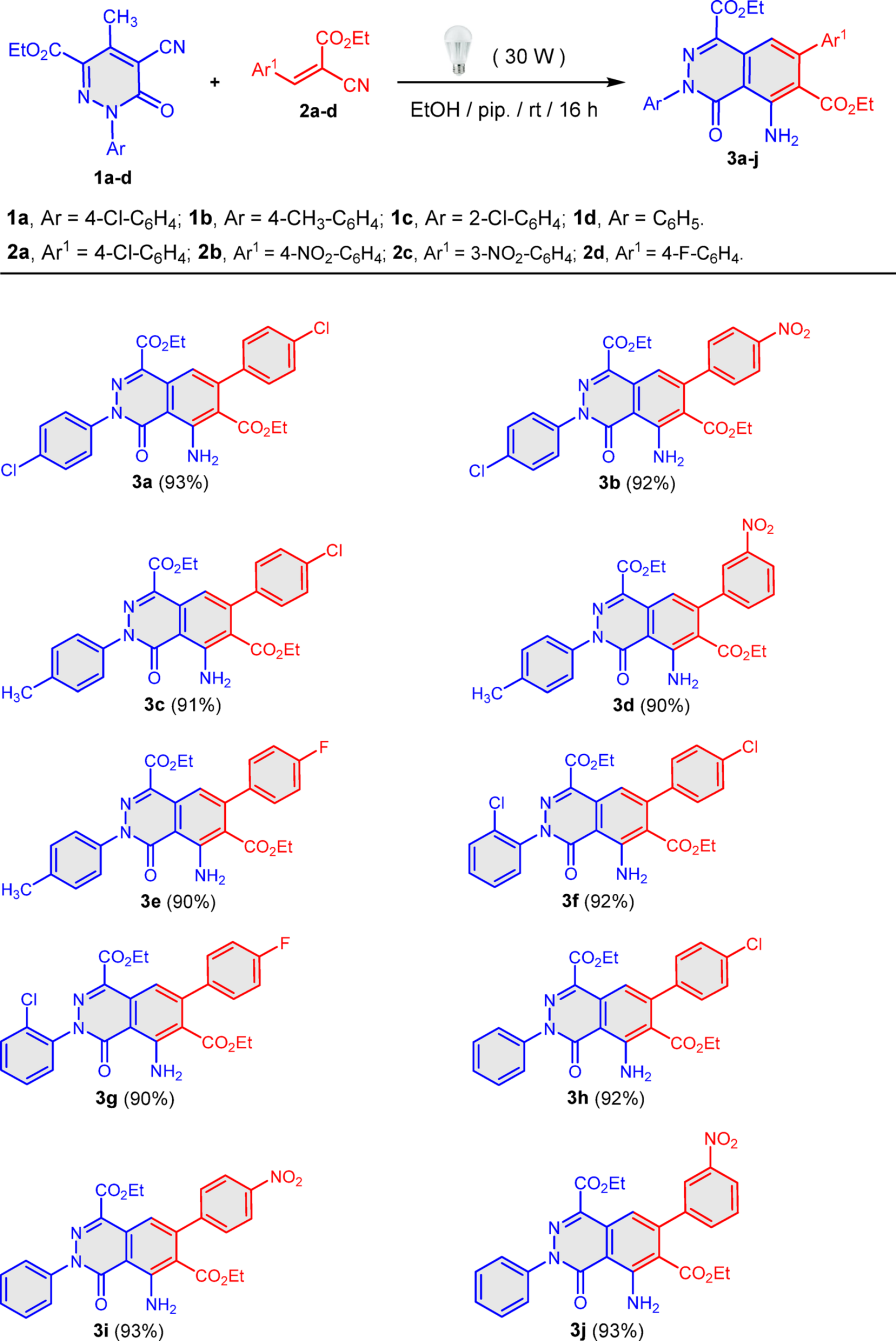
Synthesis of phthalazine derivatives **3a–j**.

With optimizing visible light conditions, the scope and limitation of the reaction was investigated utilizing variety of pyridazines **1b-d** and arylidenes **2b-d** with electron-donating and electron-withdrawing aryl groups. A diversity of polyfunctionally substituted phthalazines were obtained in pure and excellent yields (Scheme 1).

**Scheme 2:**
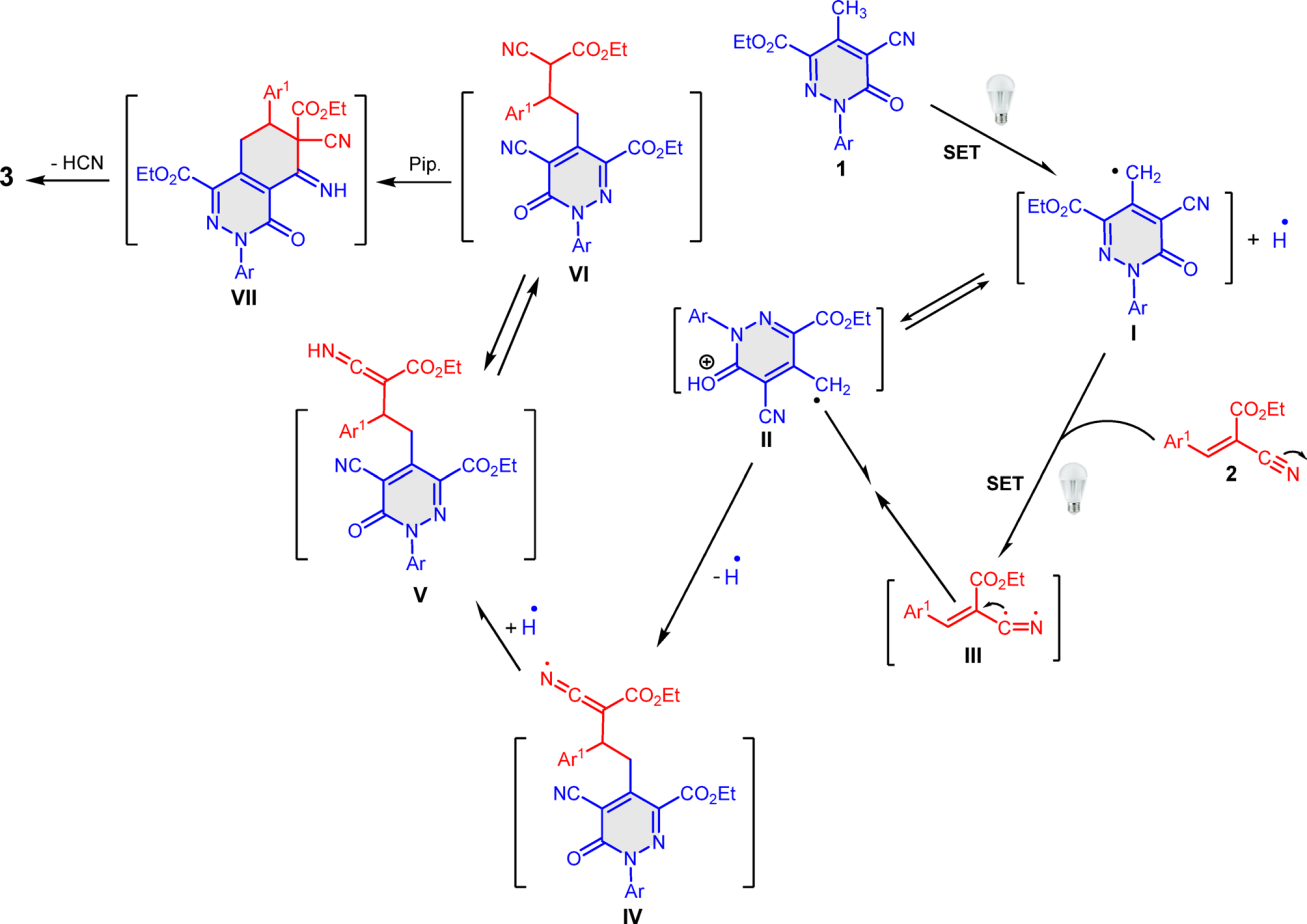
Plausible mechanism to account for the formation of the phthalazine derivatives **3a–j**.

A plausible mechanism based on control experiments and observation is proposed in Scheme 2. Firstly, pyridazine **1** with its active methyl group undergoes visible light, catalyst free, single electron transfer (SET), providing the methylene radical **I** which is in equilibrium with **II**. Visible light absorbance converted arylidene **2** to its excited state **III**. This was followed by coupling of radical **II** with **III** forming intermediate **IV** which abstracts hydrogen radial forming intermediate **V** which undergoes tautomerization and base catalyzed cyclization forming intermediate **VII**. Aromatization of **VII** *via* hydrogen cyanide loss afforded the final isolable product **3**.

### Biological assay

The cup-plate method was used to assess the phthalazines’ antibacterial efficacy against *S. aureus, Klebsiella pneumoniae*, *P. aeruginosa* and *C. albicans*. Table 2 displays the results, which showed different levels of inhibition. Compound **3g** demonstrated the best activity among the compounds studied with inhibition zones of 26 mm against *P. aeruginosa*, 21 mm against *K. pneumoniae*, and 17 mm against *C. albicans*. Compound **3j** also demonstrated significant inhibition, reaching 20 mm for *K. pneumoniae* and 25 mm for *P. aeruginosa*. With inhibition zones measuring 21–23 mm against *P. aeruginosa* and 18–19 mm against *K. pneumoniae*, **3b**, **3e** and **3i** noticeable antibacterial activity. Against *S. aureus*, the highest antibacterial activity was observed for compounds **3g**, **3j**, and **3b**, with inhibition zones of 12, 11, and 11 mm, respectively.

**Table 2:** Antimicrobial activity of the synthesized derivatives against the growth of *S. aureus, K. pneumoniae*, *P. aeruginosa* and *C. albicans* by cup-plate method.

Compound	Diameter of zone of inhibition (mm)			
	*S. aureus*	*K. pneumoniae*	*P. aeruginosa*	*C. albicans*
**3a**	7	15	18	7
**3b**	11	19	21	16
**3c**	7	14	16	7
**3d**	8	16	20	13
**3e**	10	18	23	15
**3f**	10	14	19	7
**3g**	12	21	26	17
**3 h**	7	12	14	7
**3i**	9	18	21	14
**3j**	11	20	25	16
DMSO	–	–	–	–
Ciprofloxacin	25	28	30	–
Fluconazole	–	–	–	26

*Ciprofloxacin was used as a control for bacterial strains and Fluconazole was used as control for *C. albicans*.

To further quantify the antimicrobial potency, the broth microdilution method was used to obtain the (MIC) and Minimum Bactericidal/Fungicidal Concentration (MBC/MFC) (Table 3). The findings confirmed that **3g** exhibited the strongest activity, with a MIC of 3.12 µg/mL against *P. aeruginosa* and 12.5 µg/mL against *K. pneumoniae*, followed by **3j**, which showed a MIC of 6.25 µg/mL for *P. aeruginosa*. Compounds **3b** and **3e** demonstrated moderate inhibitory effects, with MIC values ranging between 12.5 and 25 µg/mL for Gram-negative bacteria and *C. albicans*. On the other hand, **3 h** displayed the weakest antimicrobial activity, with MIC values exceeding 200 µg/mL across nearly all tested strains, indicating negligible inhibition. According to these results, **3g** and **3j** are the most promising candidates for further antimicrobial development.

**Table 3:** MIC and MBC/MFC results of phthalazine compounds for microbial strains.

*Compounds*	Determination MIC and MBC/MFC (µg/ml)							
	*S. aureus*		*K. pneumonia*		*P. aeruginosa*		*C. albicans*	
	MIC	MBC	MIC	MBC	MIC	MBC	MIC	MFC
**3a**	> 200	> 200	100	> 200	50	100	50	100
**3b**	100	> 200	25	50	12.5	25	12.5	25
**3c**	> 200	> 200	100	> 200	50	100	50	100
**3d**	> 200	> 200	50	100	25	50	25	50
**3e**	100	> 200	25	50	12.5	25	12.5	25
**3f.**	> 200	> 200	50	100	50	100	50	100
**3g**	100	> 200	12.5	25	3.12	6.25	25	50
**3 h**	> 200	> 200	> 200	> 200	100	> 200	> 200	> 200
**3i**	> 200	> 200	50	100	25	50	25	50
**3j**	100	> 200	12.5	50	6.25	25	25	50
**Ciprofloxacin**	3.12	6.25	1.56	3.12	1.56	3.12	–	–
**Fluconazole**	–	–	–	–	–	–	3.12	6.25

The microtiter plate method was used to measure the percentages of biofilm inhibition against biofilm forming strains *S. aureus*, *K. pneumoniae*, *P. aeruginosa*, and *C. albicans* in order to evaluate the antibiofilm activity of the phthalazine tested derivatives. The results revealed variations in antibiofilm efficacy among the compounds. Compound **3g** exhibited the highest biofilm inhibition, achieving 81% against *P. aeruginosa*, 65% against *K. pneumoniae*, and 60% against *C. albicans*. Compound **3j** also demonstrated strong activity, achieving 75% inhibition for *P. aeruginosa* and 72% for *K. pneumoniae*. Regarding *S. aureus*, the highest biofilm inhibition percentages were observed for compounds **3g** and **3j**, reaching 35% and 33%, respectively. These findings suggest that **3g** and **3j** are the most promising candidates for further exploration in antibiofilm. Among the other active compounds, **3b** and **3e** showed moderate antibiofilm activities, with inhibition percentages of 55 and 52% respectively for *C. albicans* and inhibition levels ranging between 58 to 71% for Gram-negative bacteria. On the other hand, **3h** showed no detectable antibiofilm activity against all tested strains (Table 4).

**Table 4:** Percentage of biofilm inhibition of phthalazine derivatives against multiresistant isolates.

Compound	% Biofilm Inhibition			
	*S. aureus*	*K. pneumoniae*	*P. aeruginosa*	*C. albicans*
**3a**	17	43	48	–
**3b**	28	60	71	55
**3c**	–	41	50	–
**3d**	25	52	60	47
**3e**	26	58	67	52
**3f**	–	52	45	35
**3g**	35	65	81	60
**3h**	–	–	–	–
**3i**	22	49	55	43
**3j**	33	72	75	58

Biofilm formation plays a crucial role in the development of multidrug-resistant infections and significantly limits the effectiveness of conventional antimicrobial therapies^28,61-72^. In this context, the antibacterial and antibiofilm activities demonstrated by the synthesized phthalazine derivatives highlight their potential as valuable candidates for the development of new agents targeting biofilm-related and drug-resistant pathogens.

### Molecular docking analysis

**Fig. 2:**
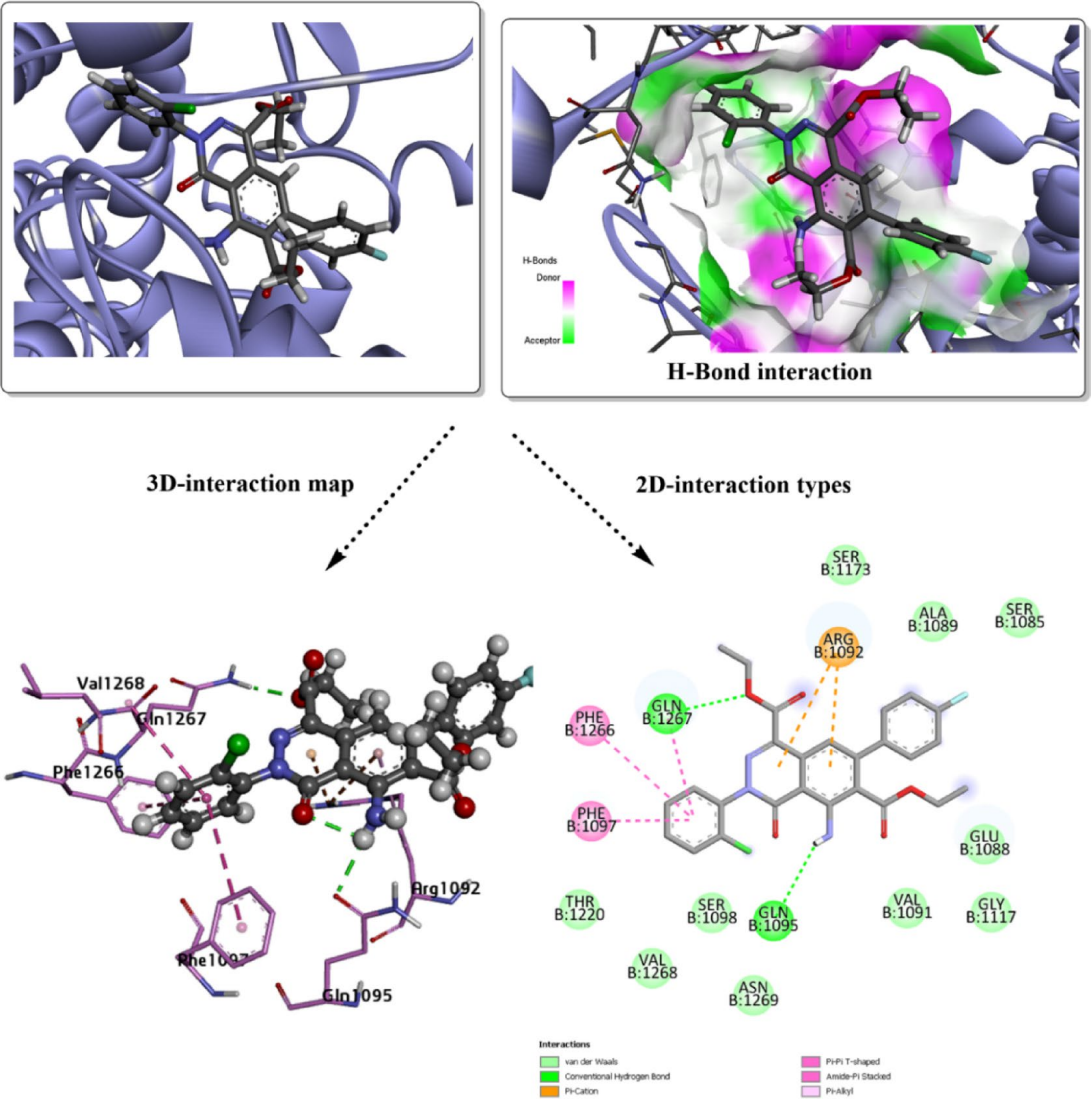
Molecular docking simulation of the best pose **3g** ligand in the predicted active sites of *2XCT*, H-bond interaction with other polar contacts using 3D and 2D maps.

**Fig. 3:**
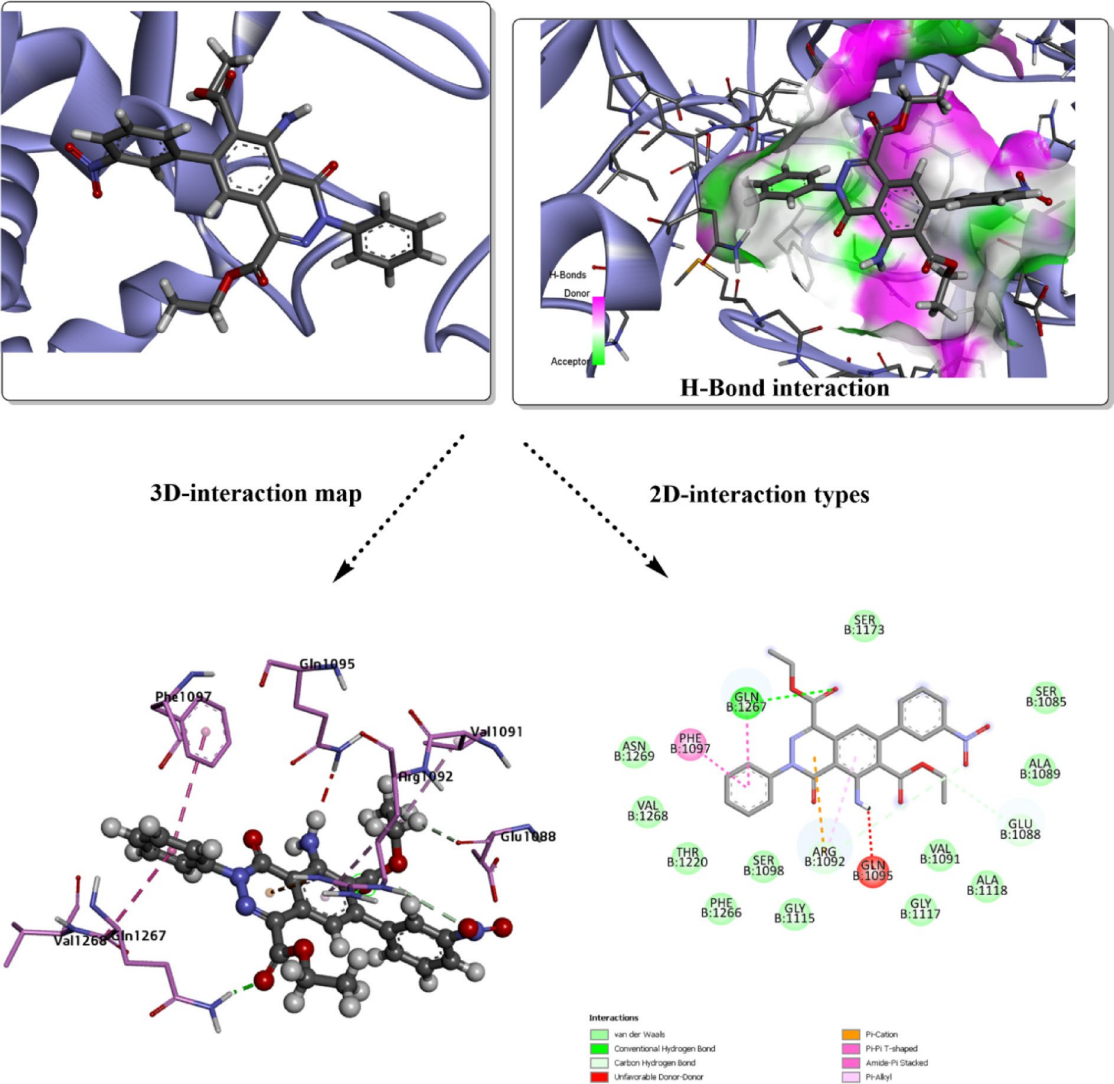
Molecular docking simulation of the best pose **3j** ligand in the predicted active sites of *2XCT*, H-bond interaction with other polar contacts using 3D and 2D maps.

The bioactive potential of the designed ligand system was investigated through in silico molecular docking simulations^73^. To evaluate the binding affinity and interaction profile, the ligand was docked into the active sites of two selected target macromolecules: *S. aureus* (ID:*2XCT*) and the key enzyme in cholesterol biosynthesis, *CYP51* (ID: *3LD6*). The docking simulations were performed to identify the most stable ligand–enzyme complexes and to predict the inhibitory potential of the compound. The results exported from the docking analysis of **3g** and **3j** against *2XCT* are shown in Figs. 2 and 3. The binding affinity property is the best control to understand the docked ligand–protein stability in its predicted active site. The total binding energy for both **3g** and **3j** was estimated with values of -6.8 kcal/mol and -7.7 kcal/mol, respectively. The predicted inhibition showed a similar impact of both ligands with common noncovalent interactions with several amino acids of the selected active site. The hydrogen bond interaction types (conventional and carbon-hydrogen bonds) are the strongest interaction type present within docked complex parts. GLN 1267 is the common interacting amino acid with **3g** and **3j** complexed against *2XCT*. Besides, GLN 1095 is present within **3g** and interacts with the O-acetate group. While the C-hydrogen type present in **3j** with ARG 1092, and GLU 1088 increases the binding affinity of the docking score, π-cation type involves ARG 1092 in both docked ligands through the aromatic rings. Other polar contacts present with PHE 1097 for both compounds, besides PHE 1266 in the **3g**-*2XCT* complex.

**Fig. 4:**
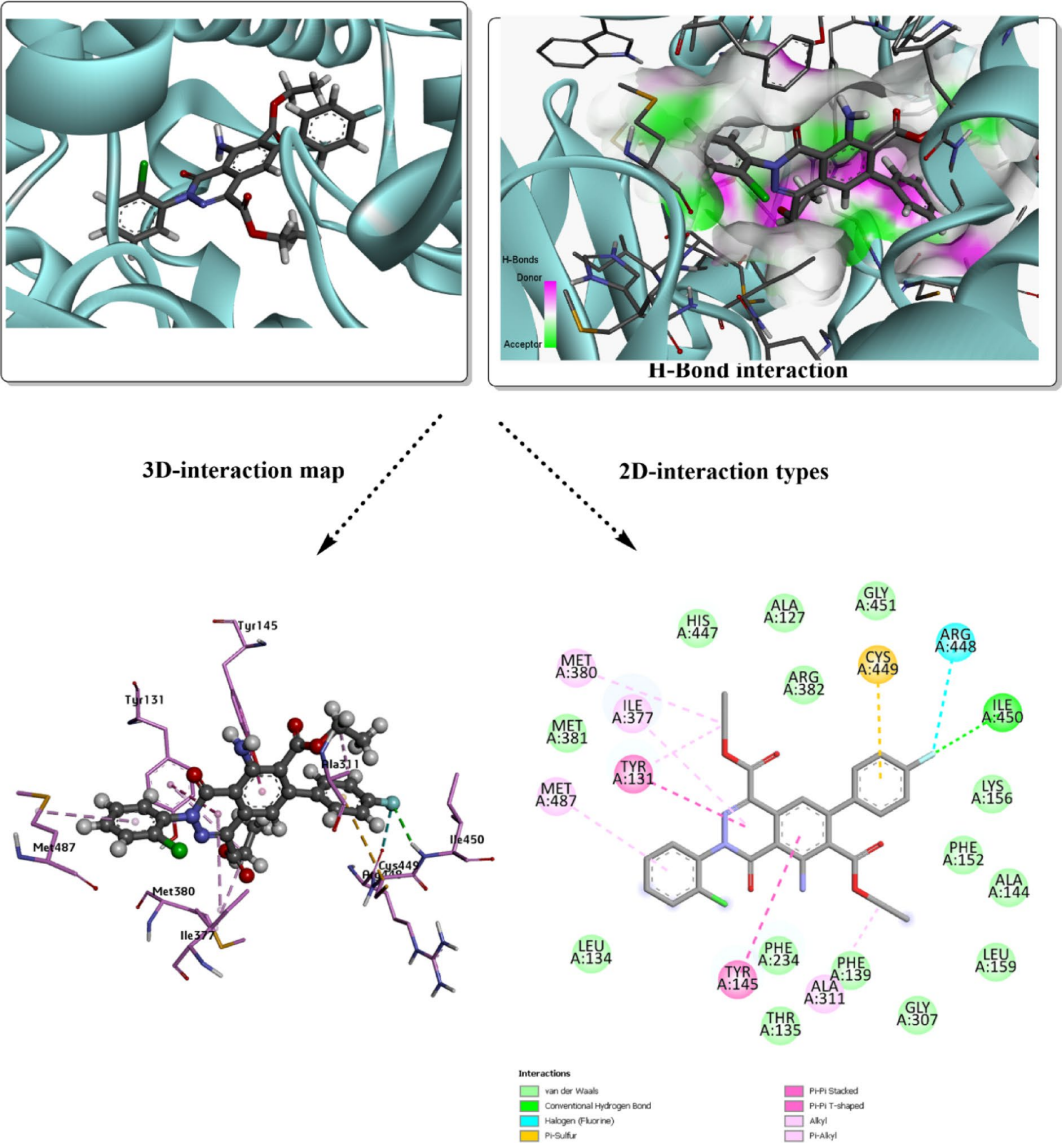
Molecular docking simulation of the best pose **3g** ligand in the predicted active sites of *3LD6*, H-bond interaction with other polar contacts using 3D and 2D maps.

**Fig. 5:**
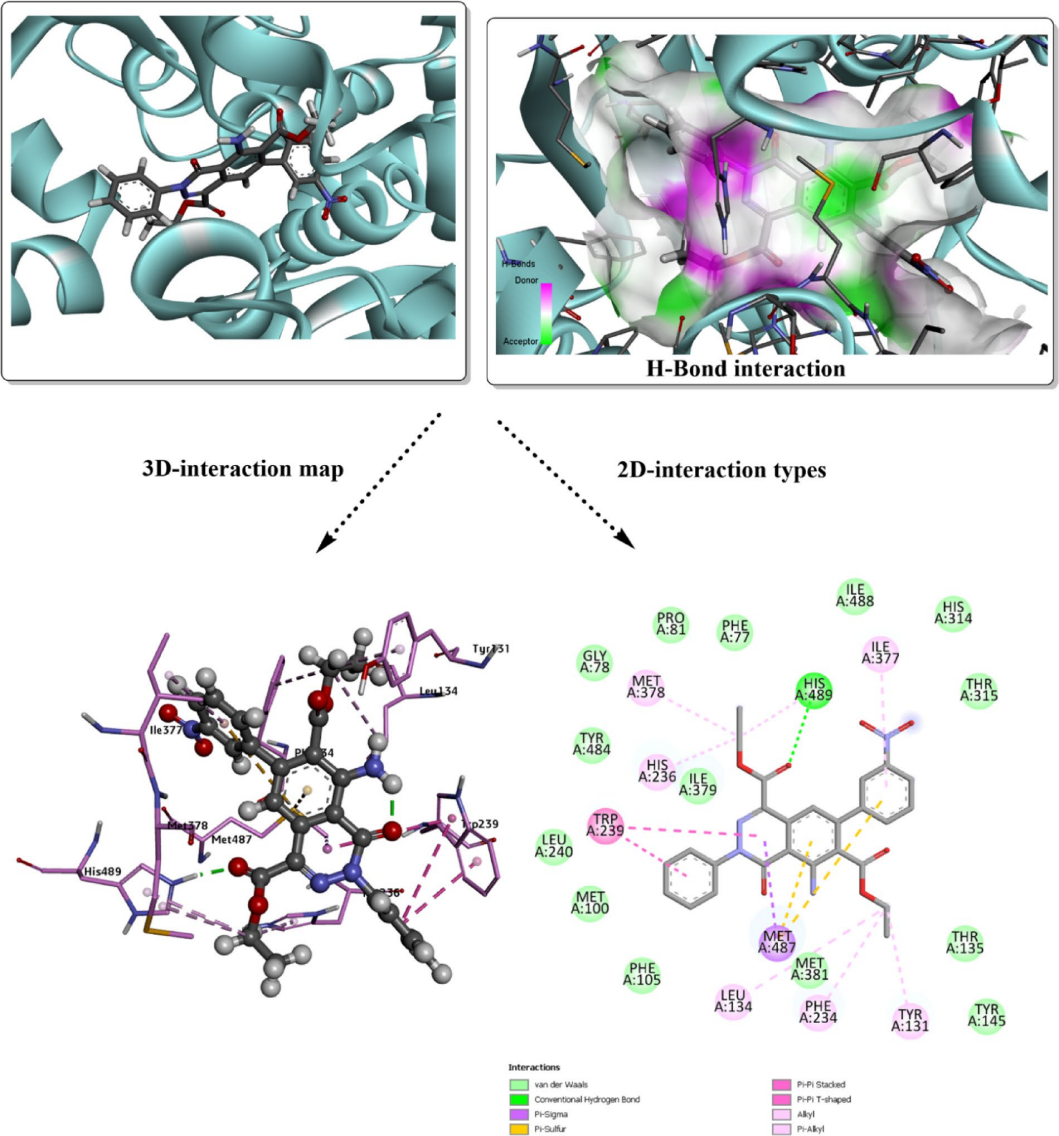
Molecular docking simulation of the best pose **3j** ligand in the predicted active sites of *3LD6*, H-bond interaction with other polar contacts using 3D and 2D maps.

Additionally, there are other amino acid residues within both docked complexes, interacting with the surface of the molecule via van der Waals (vdW) interactions. Unfavorable donor-donor interaction (GLN 1095) may destabilize the analyzed conformer within the active site, but other favorable interactions, such as H-bond types, can eliminate this unfavorable character. The higher inhibition was evaluated with *Human CYP51* (*3LD6*); the results are mapped in Figs. 4 and 5. The total binding energy for both docked conformers is estimated with values − 8.6 kcal/mol for **3g**, and − 9.5 kcal/mol for **3j**. Several interactions appear in this analysis involving the most proper H-bond type. While the interacting amino acids differ in their interaction type from one conformer to another, with the absence of common residues for the specific type. In case of **3g**, the covalent H-bond involves ILE 450, while HIS 489 is present in the **3j** predicted inhibitor. Halogen (fluorine) interaction was found with ARG 448 in **3g**-*3LD6*, while π-sigma type is present within MET 487 of **3j**-*3LD6* complex, π-sulfur type present in both complexes and several other polar interactions. vdW interactions mostly cover the surface of the designed inhibitor. H-bond donor and acceptor character were mapped for each docked structure to understand the probable domains that accept and donate on the surface of the target protein. The docking results, summarized in Table 5, compare the performance of the designed predicted ligands against both target proteins, including interaction types, and total binding energies.

**Table 5:** Binding affinity and docking score analysis for the designed heterocyclic ligands with *2XCT* and *3LD6* receptors.

Model	B.E (kcal/mol)	Ligand interacted part	Residue interacted part	Type of interaction
**3g**-*2XCT*	− 6.8	NH	GLN 1095	Conventional H-bond
		O of the acetate group	GLN 1267	Conventional H-bond
		Aromatic rings	ARG 1092	π-cation
		Chloro-Phenyl ring	PHE 1266, PHE 1097, GLN 1267	π-π T shaped, amide π-stacked, π-alkyl
**3j**-*2XCT*	− 7.7	C=O	GLN 1267	Conventional H-bond
		Ethyl group	GLU 1088	Carbon-hydrogen bond
		Ethyl group	ARG 1092	Carbon-hydrogen bond
		Pyridazine ring	ARG 1092	π-cation
		Phenyl rings	GLN 1267, PHE 1097, ARG 1092	π-π T shaped, amide π-stacked, π-alkyl
**3g**-*3LD6*	− 8.6	F	ILE 450	Conventional H-bond
		F	ARG 448	Halogen (Fluorine)
		Fluoro-aromatic ring	CYS 449	π-sulfur
		π-electrons of aromatic rings and ethyl groups	MET 380, ILE 377, TYR 131, MET 487, TYR 145, ALA 311	π-π T shaped, amide π-stacked, π-alkyl
**3j**-*3LD6*	− 9.5	C=O	HIS 489	Conventional H-bond
		Pyridazine ring	MET 487	π-sigma
		Aromatic rings	MET 487	π-sulfur
		π-electrons of aromatic rings and ethyl groups	ILE 377, MET 378, HIS 236, TRP 239, LEU 134, PHE 234, TYR 131	π-π T shaped, amide π-stacked, π-alkyl

The frontier molecular orbital (FMO) analysis and quantum chemical descriptors provide valuable insight into the potential biological activity of compounds **3g** and **3j**. In biological systems, molecular recognition and binding to biomacromolecules are largely governed by electronic interactions, such as charge transfer, hydrogen bonding, and electrostatic complementarity^74^. The smaller HOMO–LUMO energy gap of compound **3j** (3.564 eV) compared to **3g** (3.755 eV) suggests higher electronic softness and polarizability, which facilitates stronger interactions with biological targets through easier electron donation and acceptance processes. This is good evidence about the significant potential towards inhibition of 2XCT and 3DL6 targets (with binding energy values of − 7.7 kcal/mol, and − 9.5 kcal/mol, respectively, for **3j**). This enhanced reactivity implies that **3j** may exhibit improved binding affinity toward enzyme or receptor active sites. The higher electrophilicity index (ω) of **3j** reflects a greater tendency to accept electron density from nucleophilic residues such as cysteine, histidine, or serine, which are commonly present in biological active sites. Conversely, the relatively higher chemical potential (μ) of **3g** indicates a stronger electron-donating ability, which may favor hydrogen bonding and π–π stacking interactions with aromatic residues. Overall, the combined FMO features, and global reactivity descriptors suggest that both compounds possess promising biological potential, with compound **3j** expected to show stronger bioactivity due to its lower HOMO–LUMO gap, higher electrophilicity, and enhanced charge transfer capability. These electronic characteristics are consistent with the predicted molecular docking behavior and support the suitability of these compounds as potential bioactive candidates.

**Fig. 6:**
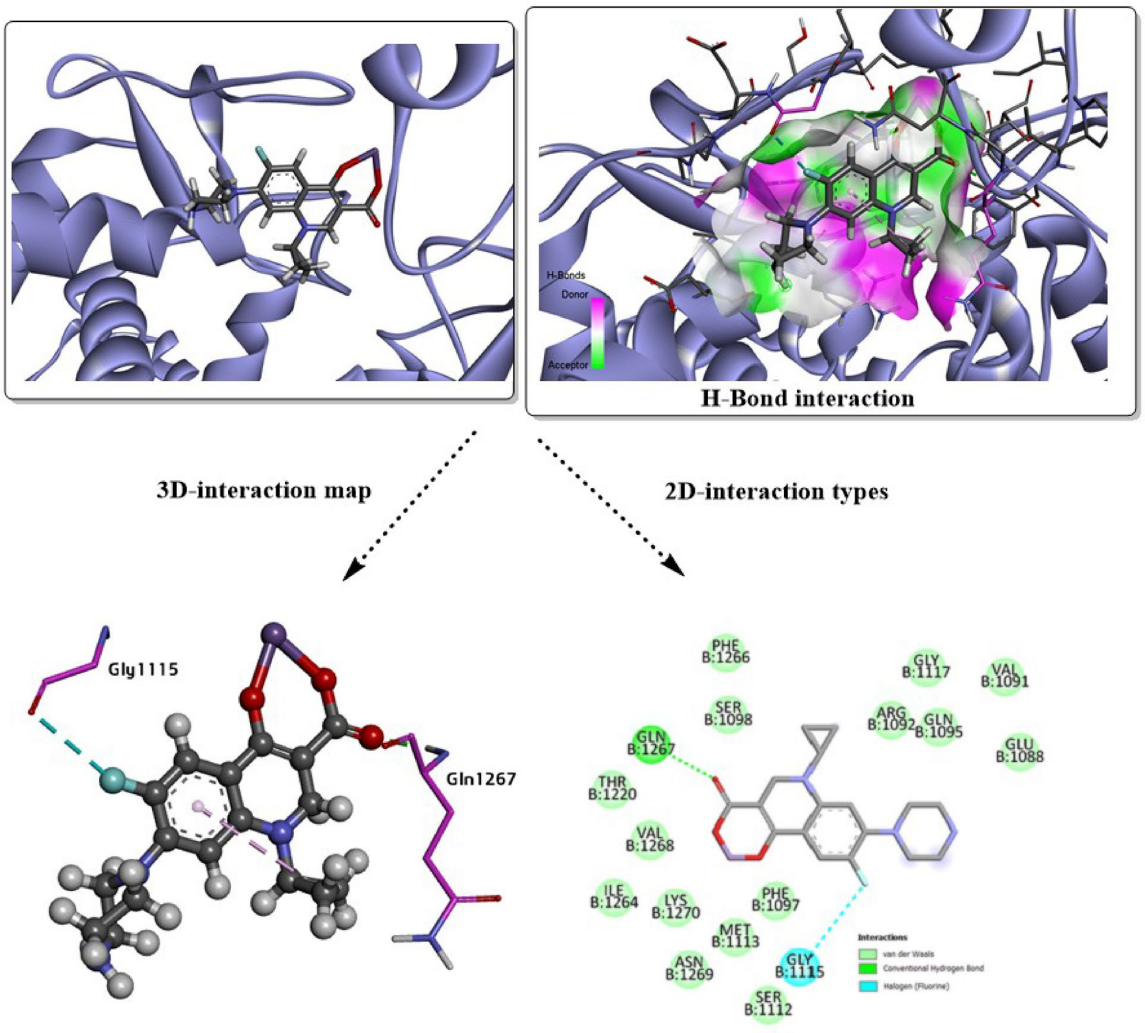
Redocking analysis of the cocrystalized ligand in the predicted active sites of *2XCT*, H-bond interaction with other polar contacts using 3D and 2D maps.

The redocking validation of the co-crystallized ligand from the *2XCT* complex in Fig. 6 demonstrates a reliable reproduction of the experimental binding mode, as evidenced by an RMSD value of 2.149 Å between the docked pose and the crystal structure. This RMSD, being close to the commonly accepted threshold of ≤ 2.0 Å, indicates good consistency and acceptable accuracy of the docking protocol in capturing the native ligand orientation within the active site. As shown in the 3D interaction map, the ligand is well accommodated in the binding pocket and maintains a spatial arrangement highly comparable to the co-crystal conformation, confirming that the key steric and electrostatic features of the site are correctly described. The hydrogen-bond interaction surface further highlights favorable donor–acceptor complementarity between the ligand and surrounding residues, supporting stable complex formation. Detailed 3D interaction analysis reveals the preservation of critical contacts, including halogen (Fluorine) and hydrogen bonding with residues such as GLY1115 and GLN1267, alongside hydrophobic and π-related interactions that anchor the aromatic core of the ligand within the pocket. The 2D interaction diagram corroborates these findings, showing a consistent interaction network involving residues like VAL1268, PHE1097, LYS1270, MET1113, GLN1267 and GLY1115, which collectively contribute to binding stabilization through van der Waals contacts, conventional hydrogen bonds, and halogen interactions.

**Fig. 7:**
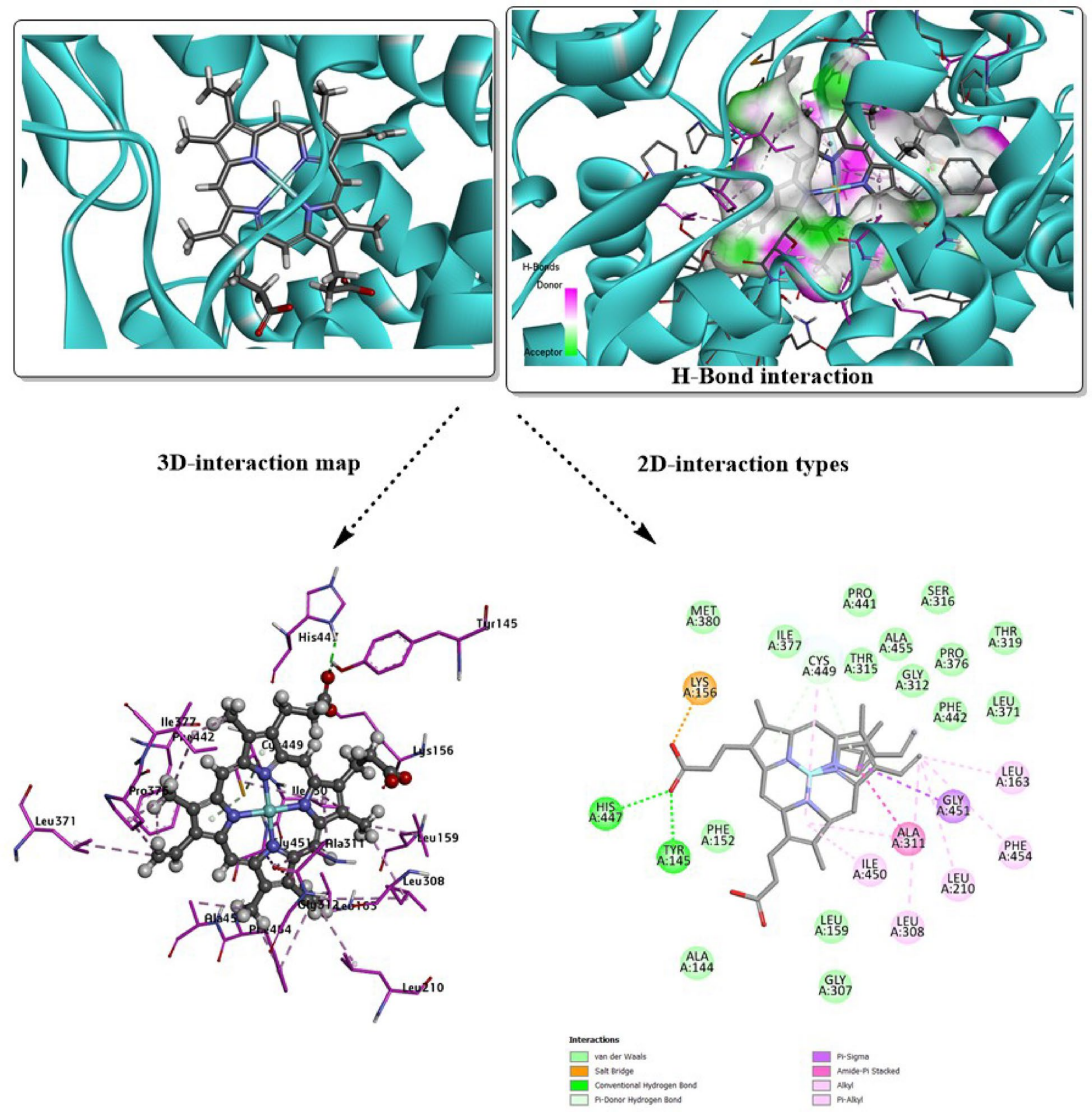
Redocking analysis of the cocrystalized ligand in the predicted active sites of *3LD6*, H-bond interaction with other polar contacts using 3D and 2D maps.

The redocking validation of the co-crystallized ligand from PDB ID *3LD6* into the protein binding site (Fig. 7) demonstrates a reliable and robust docking protocol, as evidenced by the low RMSD value of 1.877 Å between the redocked pose and the experimental crystal conformation. This RMSD, being well below the commonly accepted threshold of 2.0 Å, indicates that the docking method accurately reproduces the native binding mode and key ligand orientations within the active site. As shown in the 3D interaction maps, the ligand is deeply embedded in the binding pocket and maintains its original orientation relative to the surrounding secondary structure elements. The interaction analysis reveals the preservation of critical hydrogen bonds between the ligand heteroatoms and essential active-site residues, TYR 145, HIS 447, and CYS 449, highlighted in both the 3D hydrogen-bond surface representation and the 2D interaction diagram. In addition to hydrogen bonding, the ligand is stabilized by extensive hydrophobic and π-related interactions (π–alkyl, π–sigma, and amide–π stacking) with surrounding residues such as LEU 163, LEU 210, LEU 308, ILE 450, PHE 454, GLY 451, and ALA 311, forming a well-defined hydrophobic environment that complements the ligand scaffold. Van der Waals contacts further contribute to the tight packing of the ligand within the pocket, enhancing binding stability.

The close reproduction of the crystallographic binding modes for both *2XCT* and *3LD6* complexes, with RMSD values within the accepted ≤ 2.0 Å range, is widely regarded as a strong indicator of biologically relevant docking results. Previous studies have demonstrated that ligands maintaining key hydrogen bonds, hydrophobic contacts, and π-interactions with essential active-site residues typically exhibit significant biological activity, as these interactions are directly linked to binding affinity, target inhibition, and functional efficacy^75^. In particular, the conservation of hydrogen bonding with catalytic or recognition residues, together with hydrophobic and aromatic anchoring within the binding pocket, has been shown to correlate well with experimentally observed inhibitory potency and selectivity^76^. Therefore, the validated docking poses and preserved interaction networks observed here strongly support the biological relevance of the predicted ligand–protein complexes and justify the use of this docking protocol for interpreting biological activity and guiding further structure-based optimization.

Molecular docking is a widely accepted computational approach for predicting ligand–protein recognition, binding stability, and inhibitory potential during early-stage drug discovery, and it provides valuable insight into structure–activity relationships prior to experimental validation^76,77^. In the present study, the favorable binding energies observed for compounds **3g** and **3j** against both *2XCT* and *Human CYP51 (3LD6)* suggest the formation of thermodynamically stable complexes, which is often correlated with improved biological activity and target selectivity. Such stable noncovalent interaction patterns, particularly hydrogen bonding, π-interactions, and van der Waals contacts, have been shown to play a decisive role in ligand residence time and, consequently, pharmacokinetic behavior such as sustained target engagement and metabolic stability^78^.

From a pharmacokinetic perspective, ligands forming balanced hydrogen-bond donor/acceptor interactions and extensive hydrophobic contacts within the active site often display enhanced membrane permeability and favorable absorption profiles, while avoiding excessive polarity that may limit oral bioavailability^79^. The interaction profiles observed for **3g** and **3j**, particularly their extensive vdW surface complementarity and limited unfavorable contacts, support the hypothesis that these compounds may possess acceptable drug-like characteristics. Furthermore, targeting *CYP51*, a key enzyme in sterol biosynthesis, has been extensively validated in antifungal and anticancer research, where selective inhibition reduces systemic toxicity and off-target immune activation^80^.

In addition, ligand specificity toward microbial or cancer-related targets is an important factor in minimizing undesirable immune responses, as nonspecific binding can trigger inflammatory signaling or immunogenic side effects^81^. The observed selectivity of **3g** and **3j** toward well-defined active-site residues, rather than nonspecific surface binding, supports a reduced likelihood of immune-related adverse effects. Overall, the molecular modeling results not only rationalize the observed binding affinities but also provide a mechanistic basis for the anticipated pharmacokinetic behavior and biological compatibility of the designed heterocyclic ligands, in agreement with recent computational drug-design studies.

### Structure–activity relationship analysis

The structure–activity relationship (SAR) analysis of the most active derivatives, **3g** and **3j**, together with comparison to **3e**, clearly demonstrates that antimicrobial and antibiofilm potency is strongly governed by the electronic nature of substituents on the 3-aryl ring. Compound **3g**, bearing a para-fluorophenyl group at the 7-position and a 2-chlorophenyl moiety at the 3-position, exhibited the highest antibacterial activity (MIC = 3.12 µg/mL against *P. aeruginosa*, 81% biofilm inhibition). The presence of the ortho-chloro substituent exerts a pronounced − I electron-withdrawing effect, increasing molecular electrophilicity and polarization while also inducing favorable conformational orientation through steric influence. This combination likely enhances hydrophobic interactions, membrane penetration in Gram-negative bacteria, and stabilizing non-covalent interactions within enzymatic binding sites. In contrast, compound **3e**, which contains a p-tolyl (4-methylphenyl) group at the 3-position, showed comparatively lower activity (23 mm vs 26 mm against *P. aeruginosa*), highlighting that substitution with an electron-donating methyl group (+I effect) diminishes electrophilic character and weakens target interactions.

Similarly, compound **3j**, incorporating a meta-nitro substituent at the 3-aryl ring, also displayed strong activity (MIC = 6.25 µg/mL against P. aeruginosa; 75% biofilm inhibition). Although meta substitution limits direct resonance conjugation, the nitro group remains a powerful electron-withdrawing substituent through strong − I effects, significantly enhancing molecular polarization and electrophilicity. DFT analysis supports this observation, revealing a slightly lower HOMO–LUMO gap and higher electrophilicity index for **3j** compared to **3g**, suggesting improved interaction potential with nucleophilic residues in bacterial enzymes. Collectively, these findings establish that strong electron-withdrawing substitution at the 3-aryl ring, such as chloro groups, enhances antimicrobial potency, whereas electron-donating substituents like methyl reduce activity. Thus, electron withdrawal at this position represents a key pharmacophoric requirement, validating **3g** and **3j** as optimized lead scaffolds for further antimicrobial development.

## Conclusion

A series of new phthalazine derivatives **3a–j** was prepared and characterized with different spectroscopic and elemental activity against analyses techniques. Using Visible-Light-Mediated synthesis proved to be a simple and environmentally friendly approach with excellent yields (90–93%). The targeted compounds **3a–j** experienced antimicrobial and antibiofilm activity, compound **3g** gave the strongest activity, with a MIC value of 3.12 µg/mL against *P. aeruginosa* and 12.5 µg/mL against *K. pneumoniae* compared to Ciprofloxacin as a reference drug with a MIC value of 1.56 µg/mL. Compounds **3g** and **3j** displayed biofilm inhibition with 81% and 75%, respectively. Our protocol can pave the way for synthesis of novel heterocycles with several potential applications in pharmaceutical sciences. The DFT study reveals that **3g** is electron electron-donating system slightly more than **3j** with a less reactive structure (∆E = 3.755 eV). Molecular orbital and MEP analyses revealed electron-rich and electron-deficient regions, guiding predictions of possible interaction sites. Topological analyses through ELF and RDG/NCI further confirmed the presence of non-covalent interactions critical to molecular stability and function. Molecular docking studies demonstrated strong binding affinities of both compounds with *S. aureus* (ID:*2XCT*) and human CYP51 enzyme (3LD6), suggesting potential antibacterial and enzyme-inhibitory activity. Human CYP51 enzyme was subjected to more inhibition behavior using the designed **3g** (− 8.6 kcal/mol) and **3j**  (− 9.5 kcal/mol) structures.

## Supplementary Information

Below is the link to the electronic supplementary material.

Supplementary Material 1

## Data Availability

All data generated or analyzed during this study are included in this published article [and its supplementary information files].
