# Supplementary material for: Novel green synthesis of polyfunctionally substituted phthalazines promoted by visible light, DFT studies and molecular docking with antimicrobial and antibiofilm potency
Source: Sci Rep. 2026 May 5;16:14275. doi: 10.1038/s41598-026-47154-w (PMC13144394; doi:10.1038/s41598-026-47154-w)
Supplement: Supplementary file 1 — Supplementary Material 1 [file 41598_2026_47154_MOESM1_ESM.docx]

**Novel Green Synthesis of Polyfunctionally Substituted Phthalazines Promoted by Visible Light, DFT Studies and Molecular Docking with Antimicrobial and Antibiofilm Potency**

**Ramadan A. Mekheimer, Basma A. Khalifa, Zeinab Shawky Hashem, Samar M. R. Allam, Kamal Usef Sadek & Mohamed R. Eletmany**

**Contents**

[1. **Methodology** 2](#_Toc223680260)

[1.1. General Equation 2](#_Toc223680261)

[2. **Theoretical studies** 6](#_Toc223680262)

[2.1. Geometrical structure and Frontier molecular orbitals (FMOs) 6](#_Toc223680263)

[2.2. Quantum chemical calculations 11](#_Toc223680264)

[2.3. IR spectral analysis 12](#_Toc223680265)

[2.4. UV–Vis electronic spectra by TD-DFT method 14](#_Toc223680266)

[2.5. ^1^H NMR and ^13^C NMR Spectra 16](#_Toc223680267)

[2.6. Electron localization function (ELF) 18](#_Toc223680268)

[2.7. Localized Orbital Locator (LOL) 21](#_Toc223680269)

[2.8. Molecular electrostatic potential (MEP) 23](#_Toc223680270)

[2.9. Reduced density gradient/non-covalent-interactions (RDG/NCI) 24](#_Toc223680271)

[2. **Spectral Data** 27](#_Toc223680272)

[3. **References** 43](#_Toc223680273)

1. Methodology
   1. General Equation


***Diethyl 5-amino-3,7-bis(4-chlorophenyl)-4-oxo-3,4-dihydrophthalazine-1,6-dicarboxylate (3a).*** Yellow crystals; yield (93%); mp: 238-240^o^C. IR (KBr): *ν* = 3452, 3328 (NH_2_), 2960 (aliph. CH), 1720, 1704 (ester C=O) cm^-1^. ^1^H NMR (400 MHz): *δ_H_* = 0.76 (t, 3H, *J* = 7.2 Hz, CH_3_), 1.29 (t, 3H, *J* = 7.2 Hz, CH₃), 4.0 (q, 2H, *J* = 7.2 Hz, CH_2_), 4.36 (q, 2H, *J* = 7.2 Hz, CH_2_), 7.34 (d, *J* = 8.8 Hz, 2 Ar-H), 7.40 (s, 1 Ar-H), 7.56 (d, *J* = 8.4 Hz, 2 Ar-H), 7.60-7.66 (m, 4 Ar-H), 7.98 (br, 2H, NH_2_). ^13^C NMR (100 MHz): *δ_C_* = 13.08 (CH_3_), 13.91 (CH_3_), 60.94 (CH_2_), 61.93 (CH_2_), 110.20 (C-6), 112.35 (C-4a), 115.25 (C-8), 128.34 (2 Ar-C), 128.48 (2 Ar-C), 128.75 (2 Ar-C), 129.38 (2 Ar-C), 129.84 (1 Ar-C), 132.73 (1 Ar-C), 132.98 (1 Ar-C), 136.61 (C-1), 139.68 (C-7), 139.78 (1 Ar-C), 146.74 (C-8a), 149.90 (C-5), 160.34 (C-4), 162.58 (C=O), 166.87 (C=O). MS: (EI) *m/z* %: 526 (M^+^, 100), 451 (9), 372 (14), 302 (5), 282 (32), 256 (21), 199 (14), 111 (45). Anal. Calcd. for C_26_H_21_Cl_2_N_3_O_5_ (526.37): C, 59.33; H, 4.02; Cl, 13.47; N, 7.98%; Found: C, 59.44; H, 4.17; Cl, 13.37; N, 8.05%.

***Diethyl 5-amino-3-(4-chlorophenyl)-7-(4-nitrophenyl)-4-oxo-3,4-dihydrophthalazine-1,6-dicarboxylate (3b).*** Pale yellow crystals; yield (92%); mp: 225-227^o^C. IR (KBr): *ν* = 3456, 3324 (NH_2_), 3108 (arom. CH), 2984 (aliph. CH), 1728, 1701 (ester C=O) cm^-1^. ^1^H NMR (600 MHz): *δ_H_* = 0.76 (t, 3H, *J* = 6.0 Hz, CH_3_), 1.29 (t, 3H, *J* = 6.0 Hz, CH_3_), 3.97 (q, 2H, *J* = 6.0 Hz, CH_2_), 4.36 (q, 2H, *J* = 6.0 Hz, CH₂), 7.43 (s, 1 Ar-H), 7.60-7.66 (m, 6 Ar-H), 8.16 (br, 2H, NH_2_), 8.34 (d, *J* = 8.4 Hz, 2 Ar-H). ^13^C NMR (150 MHz): *δ_C_* = 13.51 (CH_3_), 14.41 (CH_3_), 61.57 (CH_2_), 62.47 (CH_2_), 111.39 (C-6), 112.78 (C-4a), 114.49 (C-8), 124.08 (2 Ar-C), 128.83 (2 Ar-C), 129.27 (2 Ar-C), 129.49 (2 Ar-C), 130.65 (1 Ar-C), 133.31 (1 Ar-C), 136.86 (C-1), 140.24 (C-7), 146.78 (C-8a), 147.52 (1 Ar-C), 148.33 (1 Ar-C), 150.92 (C-5), 160.80 (C-4), 163.03 (C=O), 167.03 (C=O). MS: (EI) m/z %: 538 (M^+2^, 18), 536 (M^+^, 38), 246 (46), 245 (76), 243 (98), 117 (58), 90 (80). Anal. Calcd. for C_26_H_21_ClN_4_O_7_ (536.93): C, 58.16; H, 3.94; Cl, 6.60; N, 10.43%; Found: C, 58.03; H, 4.05; Cl, 6.50; N, 10.60%.

***Diethyl 5-amino-7-(4-chlorophenyl)-4-oxo-3-(p-tolyl)-3,4-dihydrophthalazine-1,6-dicarboxylate (3c).*** Yellow crystals; yield (91%); mp: 230-232^o^C. IR (KBr): *ν* = 3422, 3304 (NH_2_), 3148 (arom. CH), 2982 (aliph. CH), 1707 (ester C=O), 1645 (amide C=O) cm^-1^. ^1^H NMR (600 MHz): *δ_H_* = 0.82 (t, 3H, *J* = 6.0 Hz, CH_3_), 1.28 (t, 3H, *J* = 6.0 Hz, CH_3_), 2.40 (s, 3H, CH_3_), 3.99 (q, 2H, *J* = 6.0 Hz, CH_2_), 4.36 (q, 2H, *J* = 6.0 Hz, CH_2_), 7.34-7.36 (m, 4 Ar-H), 7.41 (s, 1 Ar-H), 7.46 (d, 2H, *J* = 6.0 Hz, Ar-H), 7.56 (d, 2H, *J* = 6.0 Hz, Ar-H), 8.0 (br, 2H, NH_2_). ^13^C NMR (150 MHz): *δ_C_* = 13.58 (CH_3_), 14.42 (CH_3_), 21.21 (CH_3_), 61.41 (CH_2_), 62.36 (CH_2_), 110.82 (C-6), 112.75 (C-4a), 115.39 (C-8), 126.82 (2 Ar-C), 128.92 (1 Ar-C), 129.69 (2 Ar-C), 129.89 (1 Ar-C), 130.42 (1 Ar-C), 130.84 (1 Ar-C), 133.44 (1 Ar-C), 136.67 (C-1), 138.45 (1 Ar-C), 139.11 (C-7), 140.29 (C-8a), 147.19 (1 Ar-C), 150.45 (1 Ar-C), 151.17 (C-5), 163.19 (C-4), 164.23 (C=O), 167.43 (C=O). MS: (EI) m/z %: 506 (M^+^, 22), 477 (92), 387 (76), 163 (98), 97 (64). Anal. Calcd. for C_27_H_24_ClN_3_O_5_ (505.96): C, 64.10; H, 4.78; Cl, 7.01; N, 8.31%; Found: C, 64.17; H, 4.91; Cl, 7.10; N, 8.25%.

***Diethyl 5-amino-7-(3-nitrophenyl)-4-oxo-3-(p-tolyl)-3,4-dihydrophthalazine-1,6-dicarboxylate (3d).*** Yellow crystals; yield (90%); mp: 238-240^o^C. IR (KBr): *ν* = 3423, 3304 (NH_2_), 3149 (arom. CH), 2983 (aliph. CH), 1708 (ester C=O), 1645 (amide C=O) cm^-1^. ^1^H NMR (400 MHz): *δ_H_* = 0.82 (t, 3H, *J* = 6.8 Hz, CH₃), 1.29 (t, 3H, *J* = 7.2 Hz, CH_3_), 2.40 (s, 3H, CH_3_), 3.99 (q, 2H, *J* = 7.2 Hz, CH_2_), 4.36 (q, 2H, *J* = 7.2 Hz, CH_2_), 7.34 (d, 2H, *J* = 8.8 Hz, Ar-H), 7.40 (s, 1H, Ar-H), 7.56 (d, 2H, *J* = 8.8 Hz, Ar-H), 7.60-7.65 (m, 4 Ar-H), 7.97 (s, 2H, NH_2_). Anal. Calcd for C_27_H_24_N_4_O_7_: C, 62.79; H, 4.68; N, 10.85%; Found: C, 62.91; H, 4.51; N, 11.00%.

***Diethyl 5-amino-7-(4-fluorophenyl)-4-oxo-3-(p-tolyl)-3,4-dihydrophthalazine-1,6-dicarboxylate (3e).*** Yellow crystals; yield (90%); mp: 218-220^o^C. IR (KBr): *ν* = 3421, 3304 (NH_2_), 3148 (arom. CH), 2982 (aliph. CH), 1708 (ester C=O), 1644 (amide C=O) cm^-1^. ^1^H NMR (600 MHz): *δ_H_* = 0.81 (t, 3H, *J* = 6.0 Hz, CH_3_), 1.28 (t, 3H, *J* = 6.0 Hz, CH_3_), 2.40 (s, 3H, CH_3_), 3.99 (q, 2H, *J* = 6.0 Hz, CH_2_), 4.36 (q, 2H, *J* = 6.0 Hz, CH_2_), 7.34-7.36 (m, 4 Ar-H), 7.41 (s, 1 Ar-H), 7.46 (d, 2H, *J* = 6.0 Hz, Ar-H), 7.56 (d, 2H, *J* = 6.0 Hz, Ar-H), 8.0 (br, 2H, NH_2_). Anal. Calcd. for C_27_H_24_FN_3_O_5_ (489.50): C, 66.25; H, 4.94; F, 3.88; N, 8.58%; Found: C, 66.19; H, 5.02; F, 3.77; N, 8.70%.

***Diethyl 5-amino-3-(2-chlorophenyl)-7-(4-chlorophenyl)-4-oxo-3,4-dihydrophthalazine-1,6-dicarboxylate (3f).*** Yellow crystals; yield (92%); mp: 235-237^o^C. IR (KBr): *ν* = 3422, 3305 (NH_2_), 3150 (arom. CH), 2982 (aliph. CH), 1708 (ester C=O), 1644 (amide C=O) cm^-1^. ^1^H NMR (600 MHz): *δ_H_* = 0.83 (t, 3H, *J* = 6.0 Hz, CH_3_), 1.28 (t, 3H, *J* = 6.0 Hz, CH₃), 4.01 (q, 2H, *J* = 6.0 Hz, CH_2_), 4.36 (q, 2H, *J* = 6.0 Hz, CH₂), 7.37 (d, 2H, *J* = 8.0 Hz, Ar-H), 7.42 (s, 1 Ar-H), 7.46-7.58 (m, 4 Ar-H), 7.72 (s, 2 Ar-H), 7.93 (br, 2H, NH_2_). Anal. Calcd. for C_26_H_21_Cl_2_N_3_O_5_ (526.37): C, 59.33; H, 4.02; Cl, 13.47; N, 7.98%; Found: C, 59.25; H, 4.14; Cl, 13.60; N, 7.92%.

***Diethyl 5-amino-3-(2-chlorophenyl)-7-(4-fluorophenyl)-4-oxo-3,4-dihydrophthalazine-1,6-dicarboxylate (3g).*** Yellow crystals; yield (90%); mp: 248-250^o^C. IR (KBr): *ν* = 3454, 3332 (NH_2_), 2997, 2902 (aliph. CH), 1722 (ester C=O), 1708 (ester C=O), 1682 (amide C=O) cm^-1^. ^1^H NMR (600 MHz): *δ_H_* = 0.77 (t, 3H, *J* = 6.0 Hz, CH_3_), 1.27 (t, 3H, *J* = 6.0 Hz, CH_3_), 3.98 (q, 2H, *J* = 6.0 Hz, CH_2_), 4.35 (q, 2H, *J* = 6.0 Hz, CH_2_), 7.46 (s, 1 Ar-H), 7.58-7.60 (m, 2 Ar-H), 7.64 (d, 2H, *J* = 6.0 Hz, Ar-H), 7.72-7.74 (m, 2 Ar-H), 8.13 (br, 2H, NH_2_), 8.35 (d, 2H, *J* = 12.0 Hz, Ar-H). ^13^C NMR (150 MHz): *δ_C_* = 13.53 (CH_3_), 14.39 (CH_3_), 61.56 (CH_2_), 62.60 (CH_2_), 110.92 (C-6), 113.12 (C-4a), 114.94 (C-8), 124.11 (3 Ar-C), 128.92 (1 Ar-C), 129.53 (2 Ar-C), 130.43 (1 Ar-C), 130.62 (1 Ar-C), 131.48 (1 Ar-C), 131.61 (1 Ar-C), 137.35 (C-1), 138.87 (C-7), 147.02 (1 Ar-C), 147.58 (C-8a), 148.24 (1 Ar-C), 150.77 (C-5), 160.52 (C-4), 162.87 (C=O), 166.90 (C=O). Anal. Calcd. for C_26_H_21_ClFN_3_O_5_ (509.92): C, 61.24; H, 4.15; Cl, 6.95; F, 3.73; N, 8.24%; Found: C, 61.15; H, 4.31; Cl, 6.85; F, 3.61; N, 8.17%.

***Diethyl 5-amino-7-(4-chlorophenyl)-4-oxo-3-phenyl-3,4-dihydrophthalazine-1,6-dicarboxylate (3h).*** Yellow crystals; yield (90%); mp: 222-224^o^C. IR (KBr): *ν* = 3459, 3334 (NH_2_), 2991 (aliph. CH), 1722 (ester C=O), 1705 (ester C=O), 1644 (amide C=O) cm^-1^. ^1^H NMR (600 MHz): *δ_H_* = 0.82 (t, 3H, *J* = 6.0 Hz, CH₃), 1.29 (t, 3H, *J* = 6.0 Hz, CH_3_), 4.0 (q, 2H, *J* = 6.0 Hz, CH_2_), 4.36 (q, 2H, *J* = 6.0 Hz, CH_2_), 7.35 (d, 2H, *J* = 12.0 Hz, Ar-H), 7.42 (s, 1 Ar-H), 7.47-7.60 (m, 8H, 6 Ar-H and NH_2_), 7.65 (d, 1H, *J* = 6.0 Hz, Ar-H). Anal. Calcd. for C_26_H_22_ClN_3_O_5_ (491.93): C, 63.48; H, 4.51; Cl, 7.21; N, 8.54%; Found: C, 63.38; H, 4.59; Cl, 7.08; N, 8.65%.

***Diethyl 5-amino-7-(4-nitrophenyl)-4-oxo-3-phenyl-3,4-dihydrophthalazine-1,6-dicarboxylate (3i).*** Pale yellow crystals; yield (93%); mp: 226-228^o^C. IR (KBr): *ν* = 3422, 3305 (NH_2_), 3150 (arom. CH), 2982 (aliph. CH), 1708 (ester, C=O), 1644 (amide C=O) cm^-1^. ^1^H NMR (600 MHz): *δ_H_* = 0.76 (t, 3H, *J* = 6.0 Hz, CH₃), 1.28 (t, 3H, *J* = 6.0 Hz, CH_3_), 3.98 (q, 2H, *J* = 6.0 Hz, CH_2_), 4.36 (q, 2H, *J* = 6.0 Hz, CH₂), 7.45 (s, 1 Ar-H), 7.49 (s, 1 Ar-H), 7.55-7.62 (m, 8H, 6 Ar-H and NH_2_), 8.35 (d, 2H, *J* = 6.0 Hz, 2 Ar-H). ^13^C NMR (150 MHz): *δ_C_* = 13.52 (CH_3_), 14.42 (CH_3_), 61.48 (CH_2_), 62.43 (CH_2_), 111.49 (C-6), 112.72 (C-4a), 114.30 (C-8), 124.09 (2 Ar-C), 127.06 (2 Ar-C), 128.95 (1 Ar-C), 129.30 (2 Ar-C), 129.50 (2 Ar-C), 130.72 (1 Ar-C), 136.58 (C-1), 141.49 (C-7), 146.71 (C-8a), 147.51 (1 Ar-C), 148.42 (1 Ar-C), 150.97 (C-5), 160.88 (C-4), 163.12 (C=O), 167.06 (C=O). Anal. Calcd. for C_26_H_22_N_4_O_7_ (502.48): C, 62.15; H, 4.41; N, 11.15%; Found: C, 62.24; H, 4.35; N, 11.29%.

***Diethyl 5-amino-7-(3-nitrophenyl)-4-oxo-3-phenyl-3,4-dihydrophthalazine-1,6-dicarboxylate (3j).*** Yellow crystals; yield (93%); mp: 242-244^o^C. IR (KBr): *ν* = 3423, 3317 (NH_2_), 3100 (arom. CH), 1742 (ester, C=O), 1727 (ester, C=O) 1662 (amide C=O) cm^-1^. ^1^H NMR: *δ_H_* = 0.82 (t, 3H, *J* = 6.0 Hz, CH₃), 1.29 (t, 3H, *J* = 6.0 Hz, CH₃), 3.99 (q, 2H, *J* = 6.0 Hz, CH_2_), 4.36 (q, 2H, *J* = 6.0 Hz, CH_2_), 7.34-7.36 (m, 4 Ar-H), 7.4 (s, 1 Ar-H), 7.46 (m, 2 Ar-H), 7.56 (d, 2H, *J* = 12 Hz, Ar-H), 7.66 (s, 1 Ar-H), 8.0 (br, 2H, NH_2_). Anal. Calcd. for C_26_H_22_N_4_O_7_ (502.48): C, 62.15; H, 4.41; N, 11.15%. Found: C, 62.08; H, 4.54; N, 11.09%.

1. Theoretical studies

The designed phthalazine derivatives were subjected to comprehensive computational analysis *via* density functional theory (DFT) to achieve fully optimized geometrical and electronic parameters, employing the hybrid B3LYP functional.^1–4^

- 1. Geometrical structure and Frontier molecular orbitals (FMOs)

Figures 1 and 2 depict the optimized ground-state geometry of the compounds **3g** and **3j**, calculated at the same theoretical level. Two key structural parameters, bond lengths and angles, are discussed to gain deeper insight into its conformational behavior. The deviation from planarity in the molecule is primarily attributed to the orientation of the two aromatic rings and the outward projection of the ester group (CO_2_CH_2_CH_3_) from the molecular plane. The higher similarity in the structural properties makes the two studied molecules **3g** and **3j**, mostly carry similar geometrical parameters and hence electronic features. The presence of Cl atom in **3g** structure makes its related phenyl group take the horizontal plane, intersecting the vertical plane of N1-N2. This finding is the best way to describe the far-located Cl from O1, preventing steric hindrance.

The comparison between geometrical parameters of the two compounds focuses on the bond distances of the H-bond formed with several oxygen atoms. In case of **3g**, H1---O2 bond distance was estimated with a value of 2.194Å, while in **3j**, the value is 2.166Å. This slight difference may be attributed to the effect of the PhNO_2_ group of **3j**, which was classified as a strong electron-withdrawing group, resulting in further interaction of H with O atom. The hydrogen bonds of the amino groups with O1 and O5 (1.013Å and 1.006Å, respectively) are similar in both **3g** and **3j**. However, there is a very small difference in the NH_2_ bond angle (121.46^o^ and 122.67^o^, for **3g** and **3j**, respectively). This point can also be noticed for the O4-C24-O5 angle in both compounds, where it was calculated with 122.04^o^ for **3g**, and 121.54^o^, for **3j**.


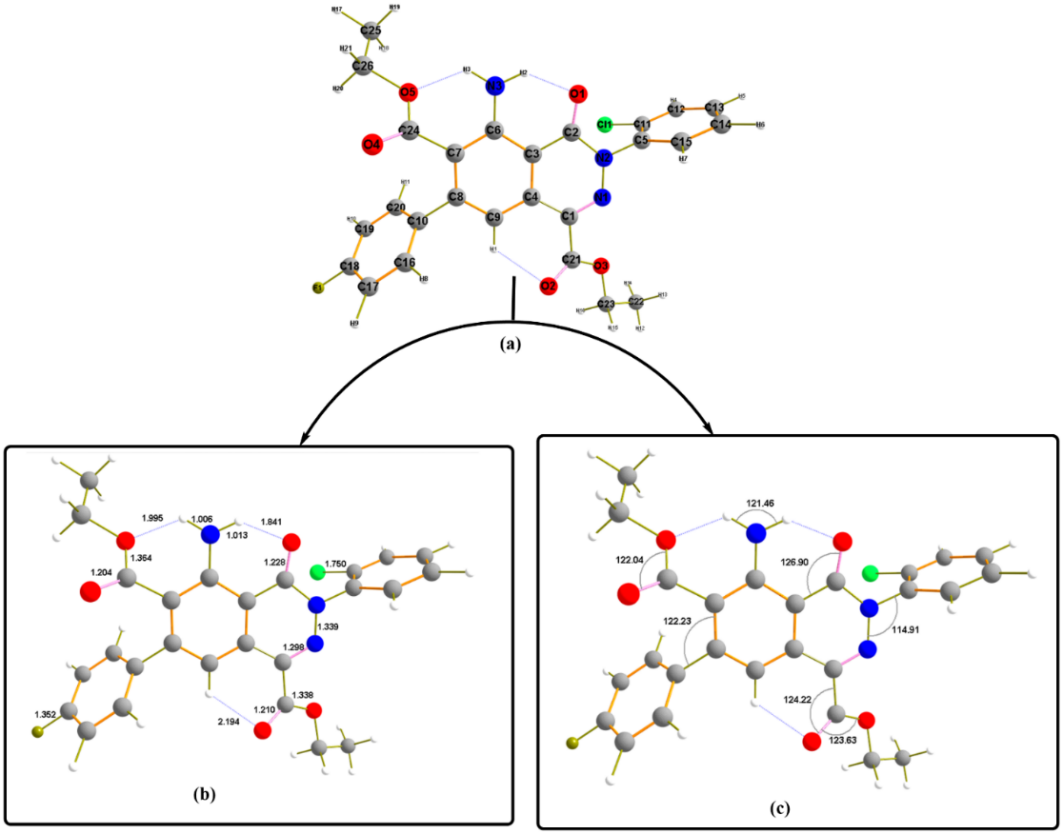


**Figure 1.** Geometrical structure of the designed compound **3g** (**a**) labeled with, (**b**) bond lengths, (**c**) bond angles.


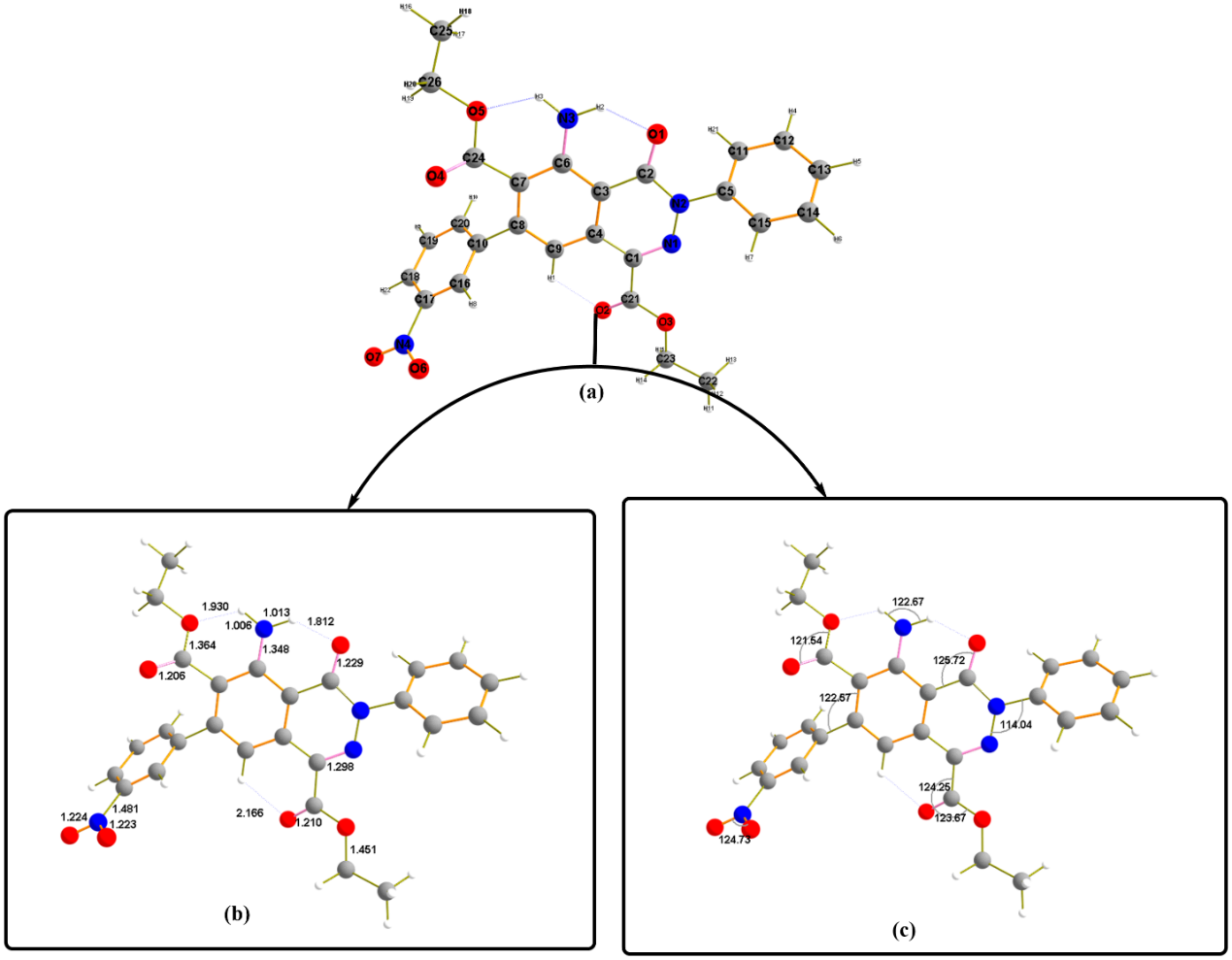


**Figure 2.** Geometrical structure of the designed compound **3j** (**a**) labeled with, (**b**) bond lengths, (**c**) bond angles.

To evaluate the stability and reactivity of molecular systems, analysis of frontier molecular orbitals (FMOs) is essential.^5^ Figures 3 and 4 illustrate the energy distribution of key molecular orbitals—including HOMO-2, HOMO-1, HOMO, LUMO, LUMO+1, and LUMO+2—for the optimized structure in the gas phase. A fundamental indicator of chemical stability, the HOMO-LUMO energy gap (∆E), was calculated to be 3.755 eV for **3g**, and 3.564 eV for **3j** suggesting that **3g** compounds possess a relatively stable electronic configuration compared with **3j**. The molecular orbital distributions across all examined levels are delocalized throughout the structure, reinforcing the stability of both ground and excited electronic states. Notably, significant orbital contributions are localized around the phthalazine ring for both **3g** and **3j**, indicating prominent donor-acceptor interactions. These localized interactions further support the compound’s potential for intramolecular charge transfer in excited states.


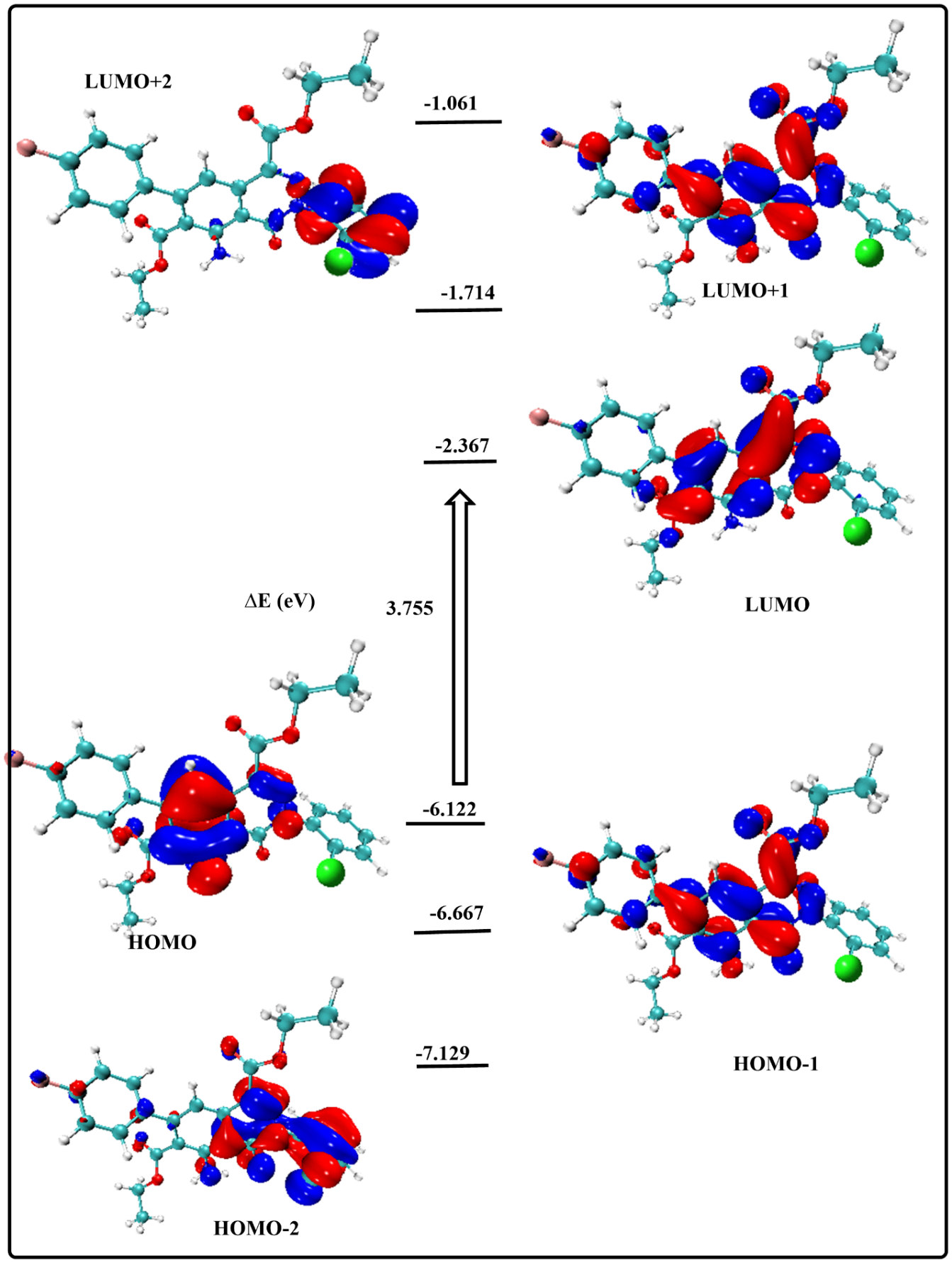


**Figure 3.** Energy excitation levels of **3g** with energy values (eV).


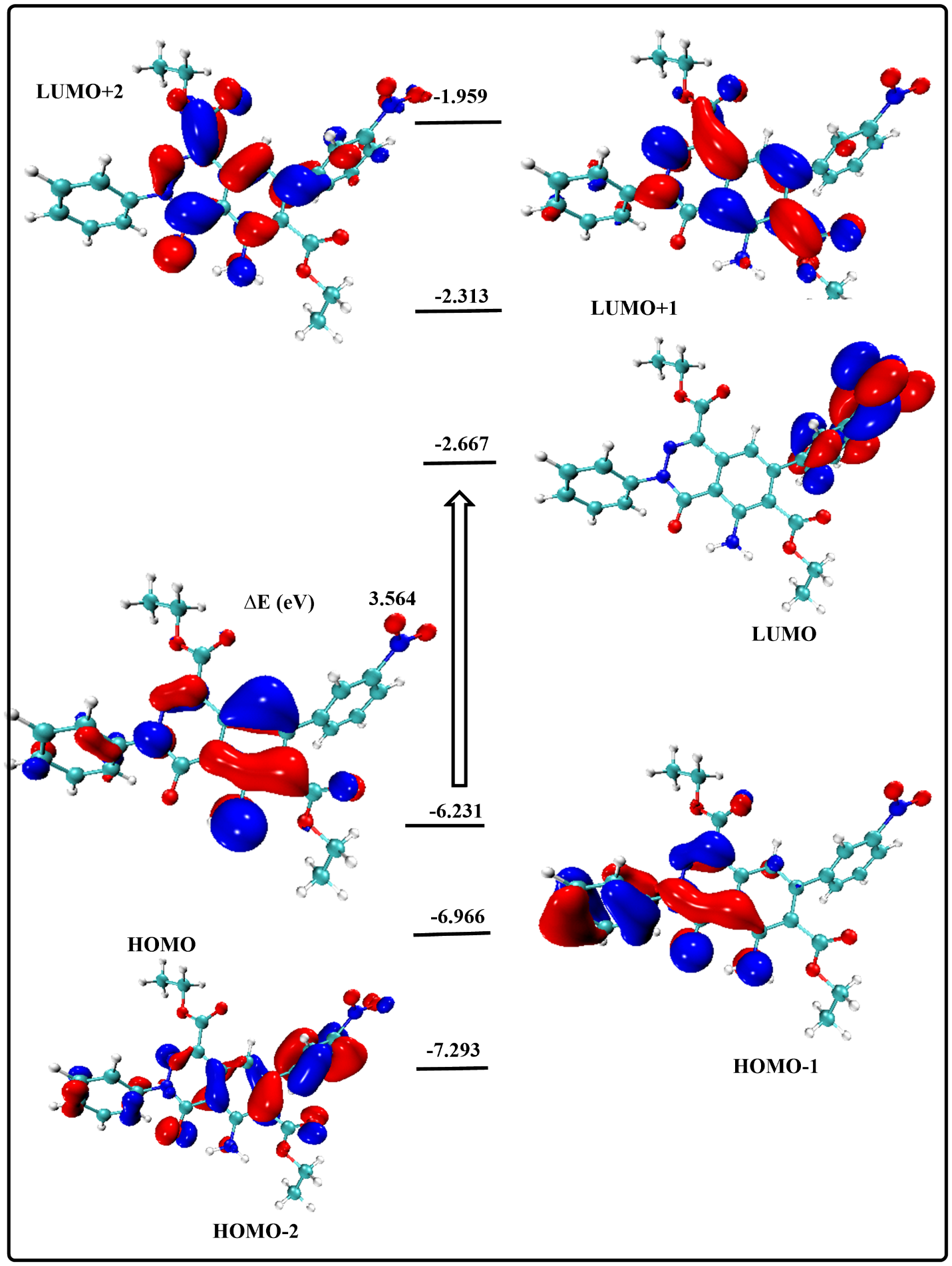


**Figure 4.** Energy excitation levels of **3j** with energy values (eV).

- 1. Quantum chemical calculations

For compounds **3g** and **3j**, quantum chemical descriptors such as chemical potential (μ), nucleophilicity (ɛ), electrophilicity (ω), and electronegativity (χ) are vital for understanding their electronic reactivity and stability. These parameters offer insight into how each compound may behave in chemical environments, including potential interactions with other species. The chemical potential (μ) indicates the energy change associated with the addition of an electron, reflecting the compound's tendency to release or accept electrons. A higher μ (less negative value) suggests a stronger driving force for electron donation, such as in the case of **3g** (-4.245 eV). Electrophilicity (ω), which quantifies a molecule’s ability to accept electron density, helps predict the compound’s behavior as an electrophile; higher ω values imply a greater capacity to interact with electron-rich species, as described for **3j** with a higher value (17.636 eV). Electronegativity (χ), representing the ability of a compound to attract electrons, is another key factor in assessing its reactivity. Compound **3j** can attract electrons slightly more than **3g** based on the ω of each compound. In other words, these descriptors enable a comparative evaluation of compounds **3g** and **3j**, aiding in the prediction of their chemical behavior and guiding the design of molecules with tailored electronic properties (Table 1).

**Table 1.** Quantum chemical parameters for the studied **3g** and **3j** using the DFT level.

| **Structure** | **E_HOMO_**  **(eV)** | **E_LUMO_**  **(eV)** | **E_GAP_**  **(eV)** | **I**  **(eV)** | **A**  **(eV)** | **μ**  **(eV)** | **Χ**  **(eV)** | **ω**  **(eV)** | **Η**  **(eV)** | **Σ**  **(eV^-1^)** |
| --- | --- | --- | --- | --- | --- | --- | --- | --- | --- | --- |
| **3g** | -6.122 | -2.367 | 3.755 | 6.122 | 2.367 | -4.245 | 4.245 | 16.912 | 1.878 | 0.533 |
| **3j** | -6.231 | -2.667 | 3.564 | 6.231 | 2.667 | -4.449 | 4.449 | 17.636 | 1.782 | 0.561 |

The HOMO–LUMO energy gap (ΔE), which is a fundamental indicator of chemical reactivity and kinetic stability, was calculated to be 3.755 eV for **3g** and 3.564 eV for **3j**. The larger energy gap of **3g** suggests higher electronic stability and lower chemical reactivity, whereas the smaller gap of **3j** indicates greater polarizability and higher reactivity toward electronic excitation or charge transfer processes. From a chemical perspective, a smaller HOMO–LUMO gap facilitates electron promotion from the HOMO to the LUMO, enhancing the molecule’s ability to participate in intermolecular interactions and reactive processes. This theoretical prediction is consistent with the experimental UV–Vis results, where compound **3j** exhibits absorption at a longer wavelength (bathochromic shift) compared with **3g**, reflecting its lower excitation energy and narrower energy gap. Thus, the TD-DFT optical behavior correlates well with the computed FMO energies.

- 1. IR spectral analysis

The vibrational infrared (IR) spectra of the studied ligands were computed to validate their optimized molecular geometries. Since quantum mechanical calculations predominantly employ harmonic oscillator models, it is essential to apply a correction factor to align the theoretical vibrational frequencies with experimental data. Table 2 presents the scaled computational frequencies alongside the corresponding experimental values for each ligand. Representative vibrational bands for the two ligands are depicted in Figures 5 and 6. As ab initio methods typically neglect anharmonic effects, the application of a functional-specific scale factor is critical to improve the accuracy of the theoretical predictions. For both compounds, two vibrational modes observed at about 3552 cm⁻¹ and 3375 cm⁻¹ correspond to N–H stretching vibrations. A weaker absorption band around 3145 cm⁻¹ is attributed to =C–H stretching, and another at 3015 cm⁻¹ corresponds to aliphatic C–H stretching. Sharp absorption peaks assigned to C=O stretching vibrations were computed at 1729 cm⁻¹ and 1717 cm⁻¹ for both **3g** and **3j**, respectively. The other predicted peaks were tabulated in Table 2 for the studied compounds.

**Figure 5.** Computational IR spectra of **3g**.

**Figure 6.** Computational IR spectra of **3j**.

**Table 2.** Calculated frequencies from B3LYP/ 6-311G(d,p) and experimental IR frequencies.

| **Functional group** | **Frequency B3LYP/6-311G(d,p)**  **(cm^-1^)** | **Frequency x scale factor**  **(cm^-1^)** |
| --- | --- | --- |
| **3g** | | |
| NH (primary) | 3674 - 3491 | 3552 - 3375 |
| =C-H (aromatic) | 3253 - 3173 | 3145 - 3068 |
| C-H (aliphatic) | 3118 - 3037 | 3015 - 2936 |
| C=O | 1788 | 1729 |
| C=C | 1649 | 1594 |
| C=N | 1594 | 1541 |
| C-F | 1255 | 1213 |
| C-O | 1107 | 1070 |
| C-Cl | 699 | 675 |
| **3j** | | |
| NH (primary) | 3673 - 3486 | 3551 - 3370 |
| =C-H (aromatic) | 3251 - 3168 | 3143-3063 |
| C-H (aliphatic) | 3118 - 3037 | 3015-2936 |
|  |  |  |
| C=O | 1776 | 1717 |
| C=C | 1663 | 1608 |
| N=O | 1602 | 1549 |
| C=N | 1598 | 1545 |
| C-O | 1116 | 1079 |

- 1. UV–Vis electronic spectra by TD-DFT method

The solvation model based on Time-Dependent Density Functional Theory (TD-DFT) combined with the Conductor-like Polarizable Continuum Model (CPCM) was employed to investigate the electronic behavior of the derivatives. All TD-DFT calculations were carried out using the default parameters of the Gaussian 09 software package. The number of excited states was set to NStates = 6 to examine the lowest six electronic transitions. Figures 7 and 8 display three prominent electronic transition bands. According to the Gaussian output log file for the two designed compounds, the first strong singlet absorption for each compound corresponds to an n → π^⁎^ electronic transition. This transition is primarily characterized by a HOMO → LUMO excitation with a contribution of 79.7%, and 70.5 % for **3g** and **3j**, respectively. The excitation energy values of 3.113 eV, and 2.856 eV for **3g** and **3j**, respectively, indicate the best electronic conjugation with further stability of **3g** molecule compared with **3j**. High excitation energy corresponds to absorption at a shorter wavelength, as shown in Table 3. The associated oscillator strength (f) is 0.218 for **3g**, indicating a high probability of electronic excitation to the LUMO. As summarized in Table 3, subsequent transitions involve excitations such as HOMO → LUMO+1 and HOMO–1 → LUMO, and HOMO→LUMO+2 with comparable percentage contributions for both **3g** and **3j**. However, the oscillator strengths vary depending on the transition pathway and the electronic overlap between the involved molecular orbitals, reflecting differences in the likelihood of each excitation.

Therefore, while HOMO–LUMO energy gaps are useful indicators of chemical reactivity and electronic stability, the assignment of λ_max_ must be based primarily on the transition with the highest oscillator strength rather than solely on the lowest-energy excitation. The TD-DFT results clearly show a marked difference in the nature of the electronic transitions for compounds **3g** and **3j**. For **3g**, the lowest-energy transition at 3.113 eV (λ_max_ = 398 nm) corresponds to a HOMO → LUMO excitation with a relatively high oscillator strength (f = 0.218), indicating an allowed and intense transition. This transition therefore represents the dominant absorption band and can be reliably assigned as the main λ_max_ of **3g**. The subsequent transitions (HOMO → LUMO+1 and HOMO–1 → LUMO) exhibit lower oscillator strengths (f = 0.121 and 0.047, respectively), confirming their weaker contributions to the overall absorption spectrum. In contrast, for **3j**, although the lowest-energy transition at 2.856 eV (λ_max_ = 434 nm) is mainly characterized by a HOMO → LUMO excitation (70.5%), its oscillator strength is extremely low (f = 0.005), indicating a near-forbidden transition with negligible intensity. The strongest absorption for **3j** instead arises from the second excited state at 3.046 eV (λ_max_ = 407 nm), corresponding to a HOMO → LUMO+1 transition with a significantly higher oscillator strength (f = 0.358).


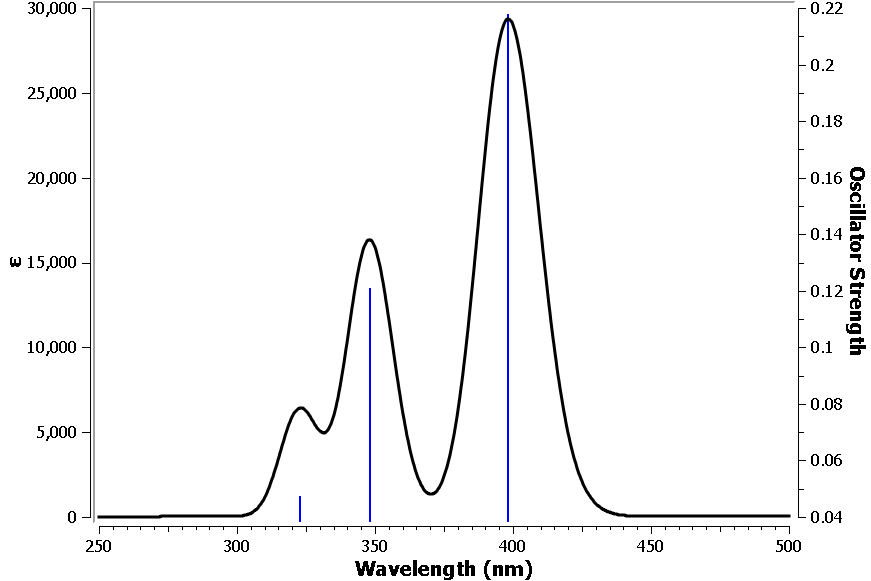


**Figure 7.** UV–Vis electronic absorption spectra for **3g**.


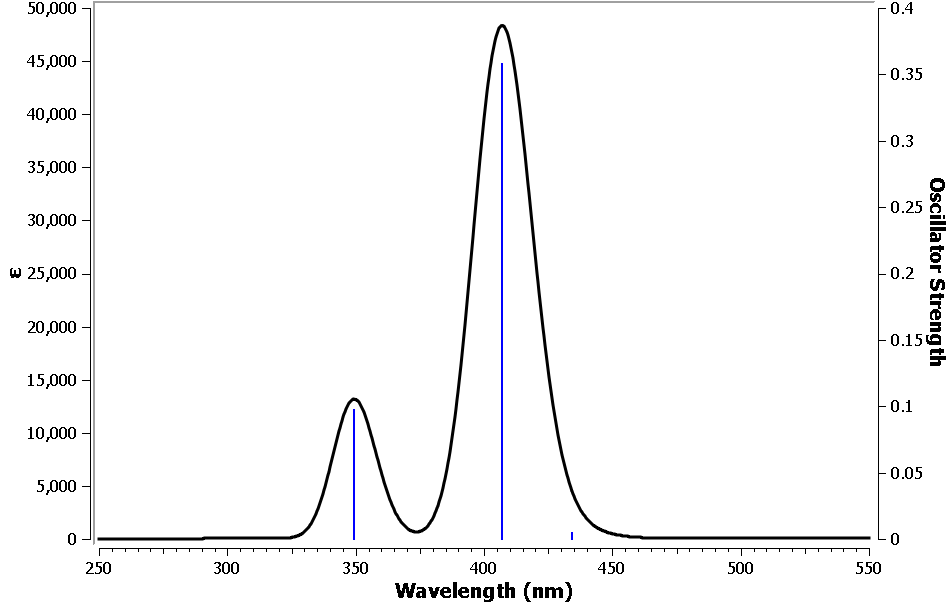


**Figure 8.** UV–Vis electronic absorption spectra for **3j**.

**Table 3.** Excitation energies, maximum wavelengths, oscillator strengths and % orbital contribution for **3g** and **3j**.

| **Spectral line number** | **Excitation energy (eV)** | **λ_max_ (nm)** | **F** | **Type of transition** | **% orbital contribution** |
| --- | --- | --- | --- | --- | --- |
| **3g** | | | | | |
| 1 | 3.113 | 398 | 0.218 | HOMO→LUMO | 69.7 |
| 2 | 3.562 | 348 | 0.121 | HOMO→LUMO+1 | 68.2 |
| 3 | 3.841 | 322 | 0.047 | HOMO-1→LUMO | 67.8 |
| **3j** | | | | | |
| 1 | 2.856 | 434 | 0.005 | HOMO→LUMO | 70.5 |
| 2 | 3.046 | 407 | 0.358 | HOMO→LUMO+1 | 69.9 |
| 3 | 3.548 | 349 | 0.097 | HOMO→LUMO+2 | 67.9 |

- 1. ^1^H NMR and ^13^C NMR Spectra

The computational ^1^H NMR spectrum of compounds **3g** and **3j**, simulated using the GIAO method, Figures 9a and 10a display characteristic chemical shifts ranging from 0 to 14 ppm, with distinct signal groupings indicating the presence of aromatic and aliphatic hydrogen environments. A prominent peak around 8.0 ppm suggests a set of equivalent aromatic protons. Additional peaks in the 3.5–4.0 ppm region point to methylene or methine protons adjacent to electronegative atoms, while signals between 1.0 and 2.0 ppm correspond to aliphatic methyl or methylene groups. The highly shifted protons to about 9.3 ppm corresponded to the NH_2_ group. Overall, the spectrum supports the structural presence of an aromatic ring, electronegative substituents, and aliphatic chains within compounds **3g** and **3j**.

Figures 9b and 10b display a simulated ^13^C NMR spectrum of **3g** and **3j** derivatives ranging approximately from 0 to 180 ppm, with multiple sharp peaks indicating distinct carbon environments. Many of the signals appear in the aromatic and unsaturated carbon region (110–170 ppm), suggesting the presence of aromatic or conjugated structures. Signals near 60 ppm and below 20 ppm are indicative of aliphatic carbons or possibly carbon atoms attached to electronegative groups.

On the other hand, Actual spectra from 600 MHz ¹H NMR and 150 MHz ¹³C NMR displayed the following key shifts: CH₃ (^1^H: 0.77, 1.27 ppm; ¹³C: 13.53, 14.39 ppm); CH₂ (^1^H: 3.98, 4.35 ppm; ¹³C: 61.56, 62.60 ppm); aromatic protons (7.46-8.35 ppm); broad NH₂ (8.13 ppm). ¹³C NMR signals for aromatic/carbonyl carbons appeared at 110-166 ppm.

Hydrogen bonding interactions were further supported by computational analysis and correlated with the experimental ¹H and ¹³C NMR spectra. From the optimized geometry, the presence of intramolecular/intermolecular H-bonds was confirmed by short donor–acceptor distances and favorable hydrogen bond angles, indicating stable H-bond formation. These interactions significantly influence the electronic environment of the involved nuclei, which is reflected in the NMR chemical shifts. In the ¹H NMR spectrum, protons participating in hydrogen bonding, such as –NH2 group in both compounds, exhibit noticeable downfield shifts due to deshielding effects arising from electron density withdrawal toward the hydrogen bond acceptor. Similarly, in the ¹³C NMR spectrum, carbon atoms bonded to heteroatoms involved in H-bonding (as for C–O or C–N) show downfield displacement compared to non-hydrogen-bonded carbons, consistent with reduced electron density.


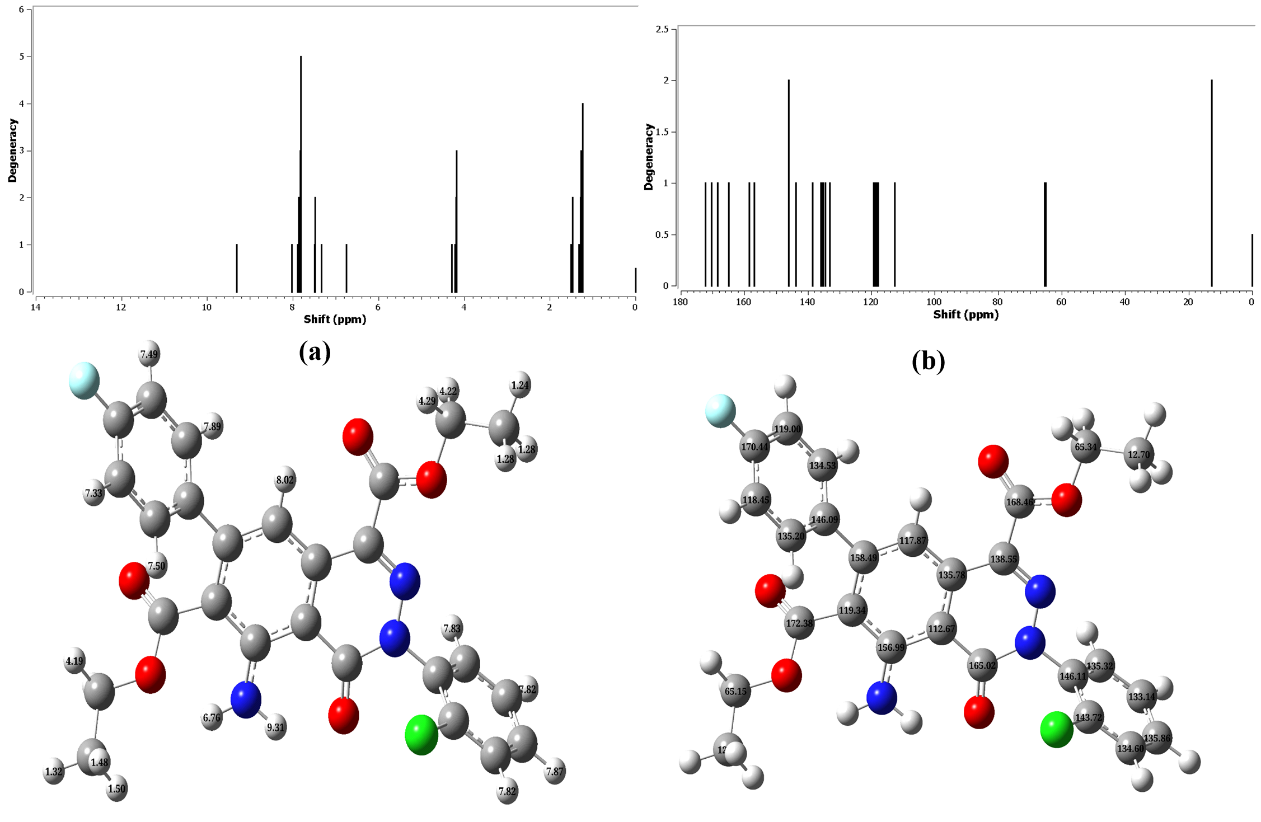


**Figure 9.** Computational (a) ^1^H‑NMR and (b) ^13^C‑NMR spectra of **3g** compound using GIAO method.


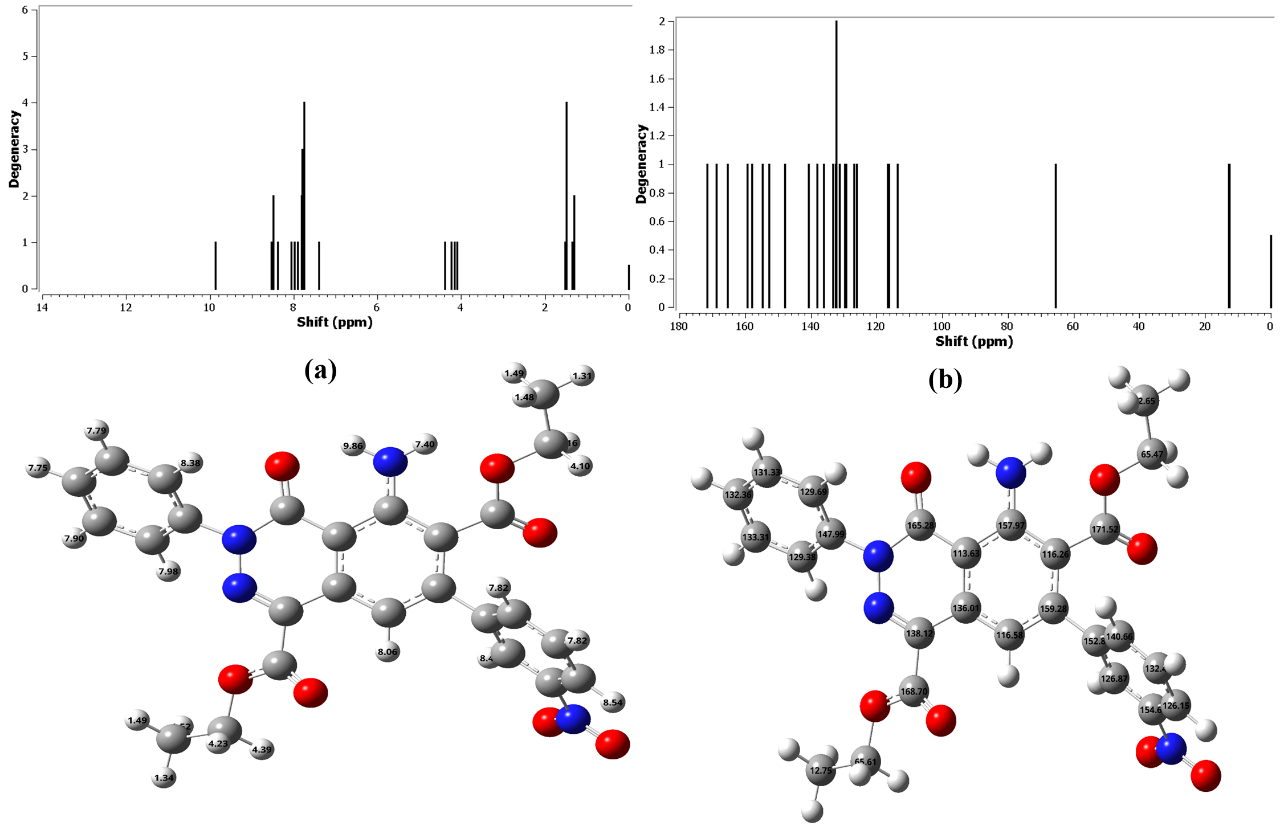


**Figure 10.** Computational (**a**) ^1^H‑NMR and (**b**) ^13^C‑NMR spectra of **3j** compound using GIAO method.

- 1. Electron localization function (ELF)

The electron localization function (ELF) provides a quantitative approach to analyzing electron pair localization, aligning with the conceptual framework of Lewis structures. It identifies regions in atomic space where electron localization is maximized, offering insights into the nature of chemical bonding and electron confinement.^6^ In this study, several key two-dimensional planes, O4-C24-O5, H2-N3-H3, C7-C8-C10, and C8-C7-C24, were analyzed for both **3g** and **3j** molecules to elucidate bonding characteristics. All atoms of interest lie within the same plane, as depicted in Figures 11 and 12, enabling precise evaluation of electron density distribution. O4–C24–O5 plane, in both designed compounds, displays pronounced electron localization between carbon and oxygen atoms, as indicated by the concentrated red regions near O atoms and the bonding basins extending toward the central carbon, suggesting strong, polar covalent C=O or C–O bonds. The H2–N3–H3 plane shows a more diffuse ELF distribution along the N–H bonds, indicative of moderately localized bonding with potential for hydrogen bonding interactions due to the lone pair on N3. However, NH_2_ plane in, **3j**, takes a more significant planarity with the surrounding atoms such as O1 and related carbon atoms (appear in Figure 12b). The C7–C8–C10 plane, reveals significant ELF delocalization across the carbon atoms, with bonding basins shared between them, characteristic of conjugated or delocalized π-systems. The C8–C7–C24 plane illustrates an intermediate case where electron localization is observed in C–C bonds but with slight asymmetry in the ELF distribution, possibly due to substituent effects or hybridization differences along the C7–C24 axis. The **3g** plane includes an ethyl group attached to O5, which describes a more planar structure.


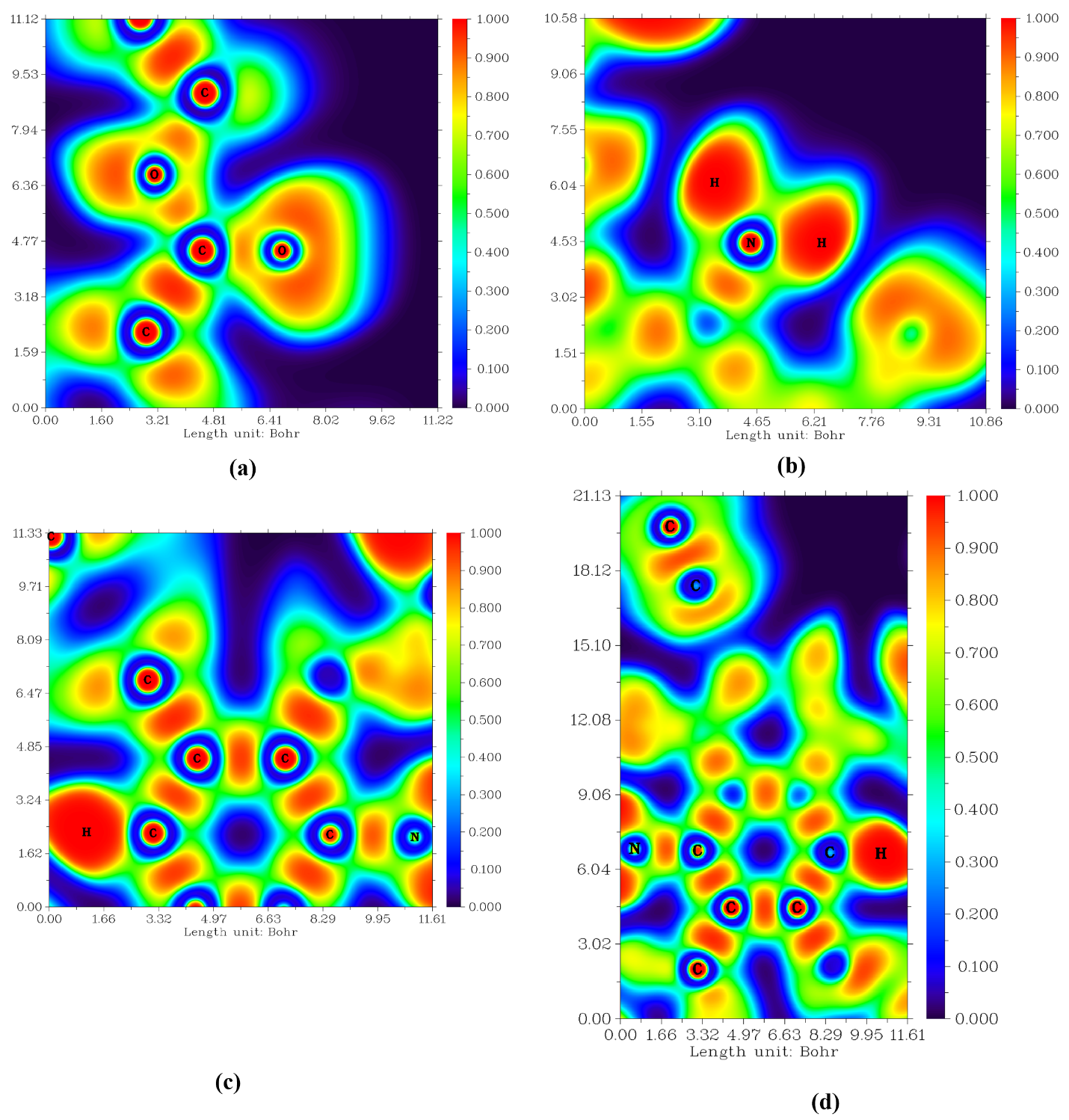


**Figure 11.** Electron localization function (ELF) colored map of 3g (**a**) O4-C24-O5, (**b**) H2-N3-H3, (**c**) C7-C8-C10, and (**d**) C8-C7-C24.


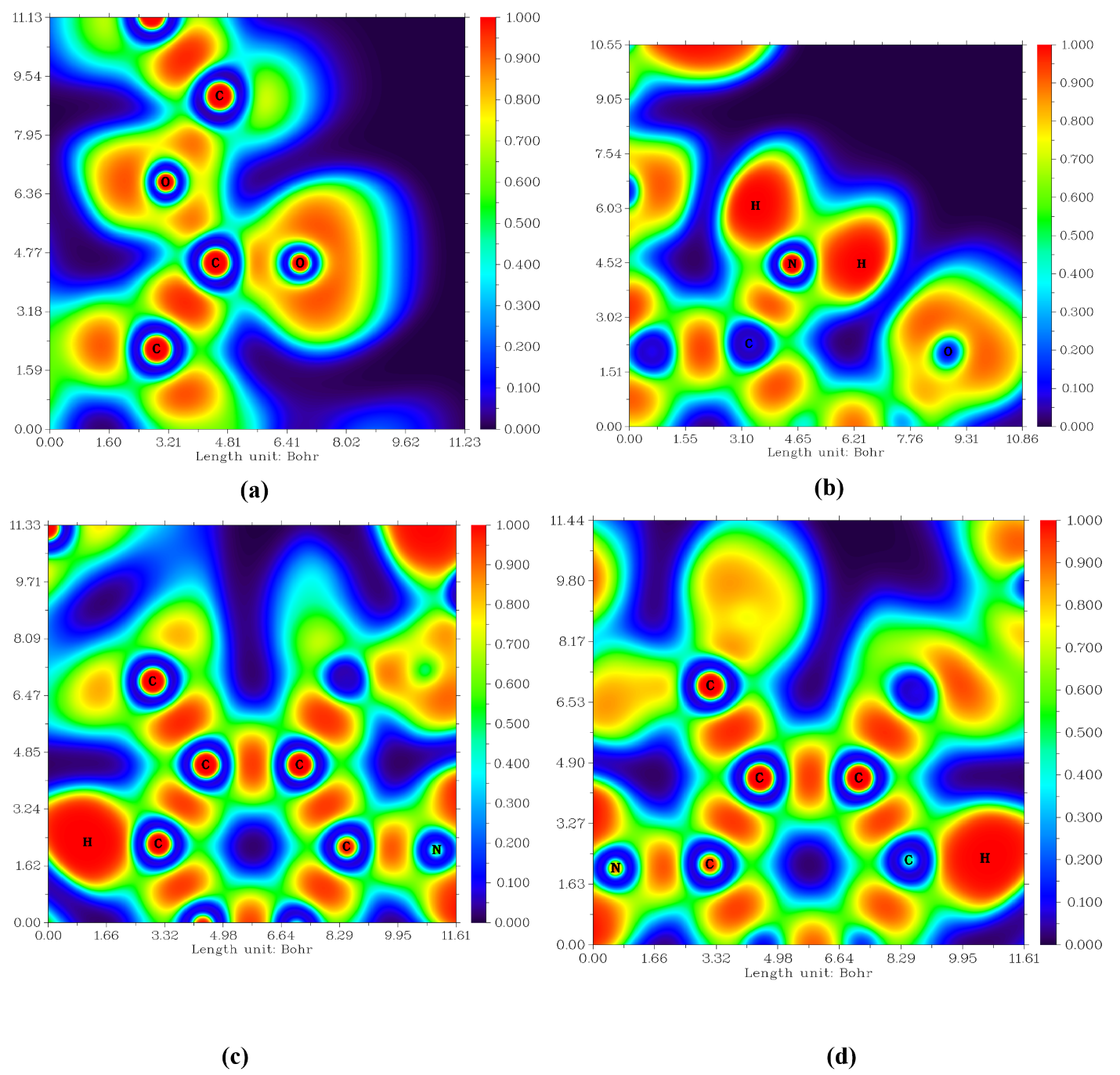


**Figure 12.** Electron localization function (ELF) colored map of **3j** (**a**) O4-C24-O5, (**b**) H2-N3-H3, (**c**) C7-C8-C10, and (**d**) C8-C7-C24.

In the case of the XY plane for both **3g** and **3j** compounds (Figure 13), the 2D ELF projections show how electrons are localized and delocalized throughout the molecular frameworks along that plane. For compound **3g**, the XY plane map highlights moderate π-electron delocalization across the main conjugated backbone, seen as extended yellow–green regions, while strongly localized electron pairs remain centered near heteroatoms, especially oxygen, as intense red basins. This pattern reflects a balanced electronic structure with clear conjugation interspersed with localized lone pairs, consistent with the typical interpretation of ELF contours where high values mark localized electron density such as lone pairs and covalent bonds and lower values indicate delocalization pathways.^7^

In contrast, compound **3j** shows a more intricate XY plane ELF distribution. The heightened heteroatom content manifests in more pronounced and spatially broader high ELF regions around oxygen and nitrogen, corresponding to stronger lone-pair localization. Additionally, the central π system exhibits extensive continuous yellow–green zones, indicating enhanced π-electron delocalization throughout the conjugated core. Relative to **3g**, the ELF features in **3j**’s XY plane suggest intensified electron localization near heteroatoms along with more extended delocalized π networks, implying increased polarization and electron mobility across the plane. These features are typical of systems with greater heteroatom integration and conjugation, as higher ELF values demarcate regions where electron pairing, whether from lone pairs or bonding electrons, is more probable (Figure 13).^8^


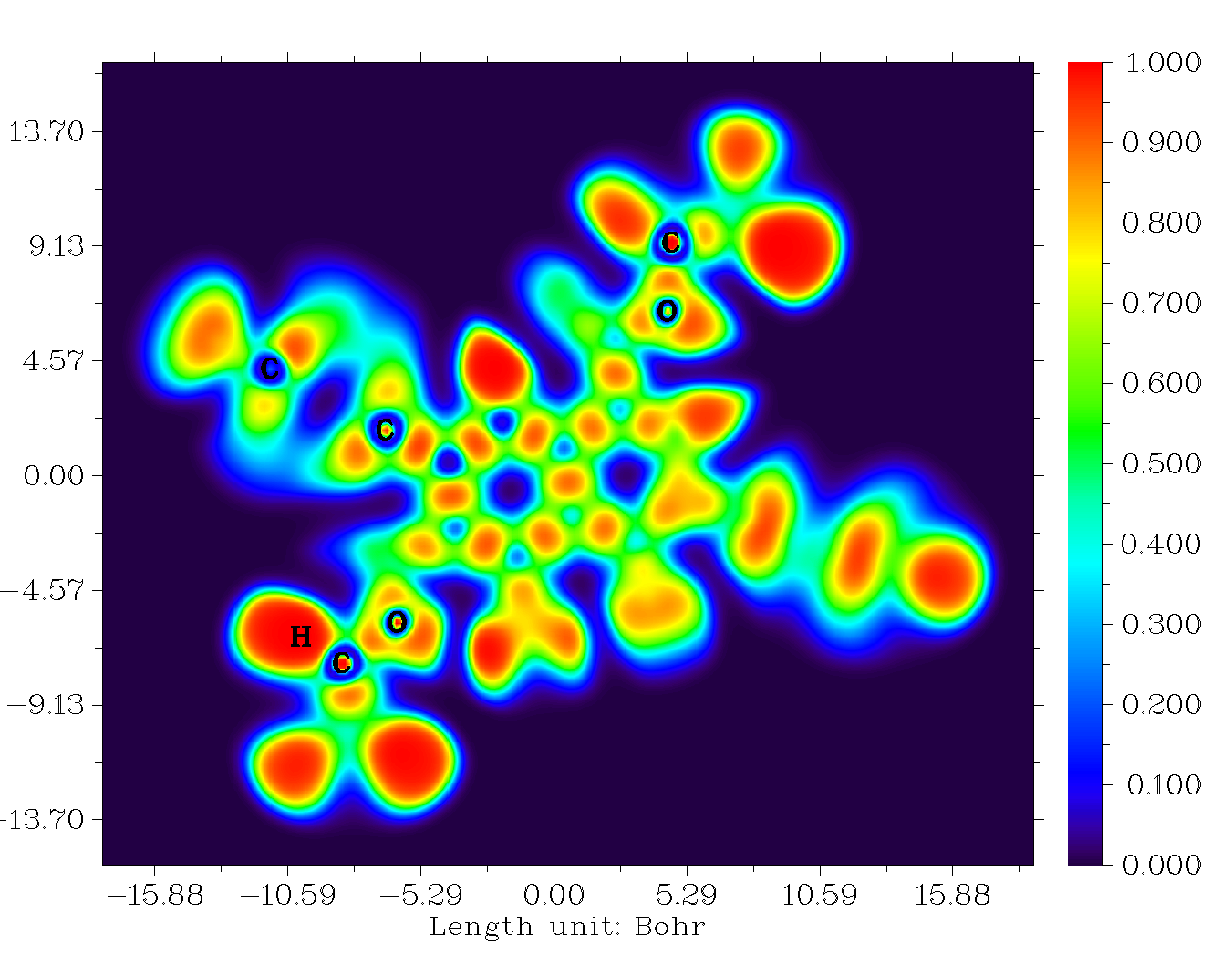

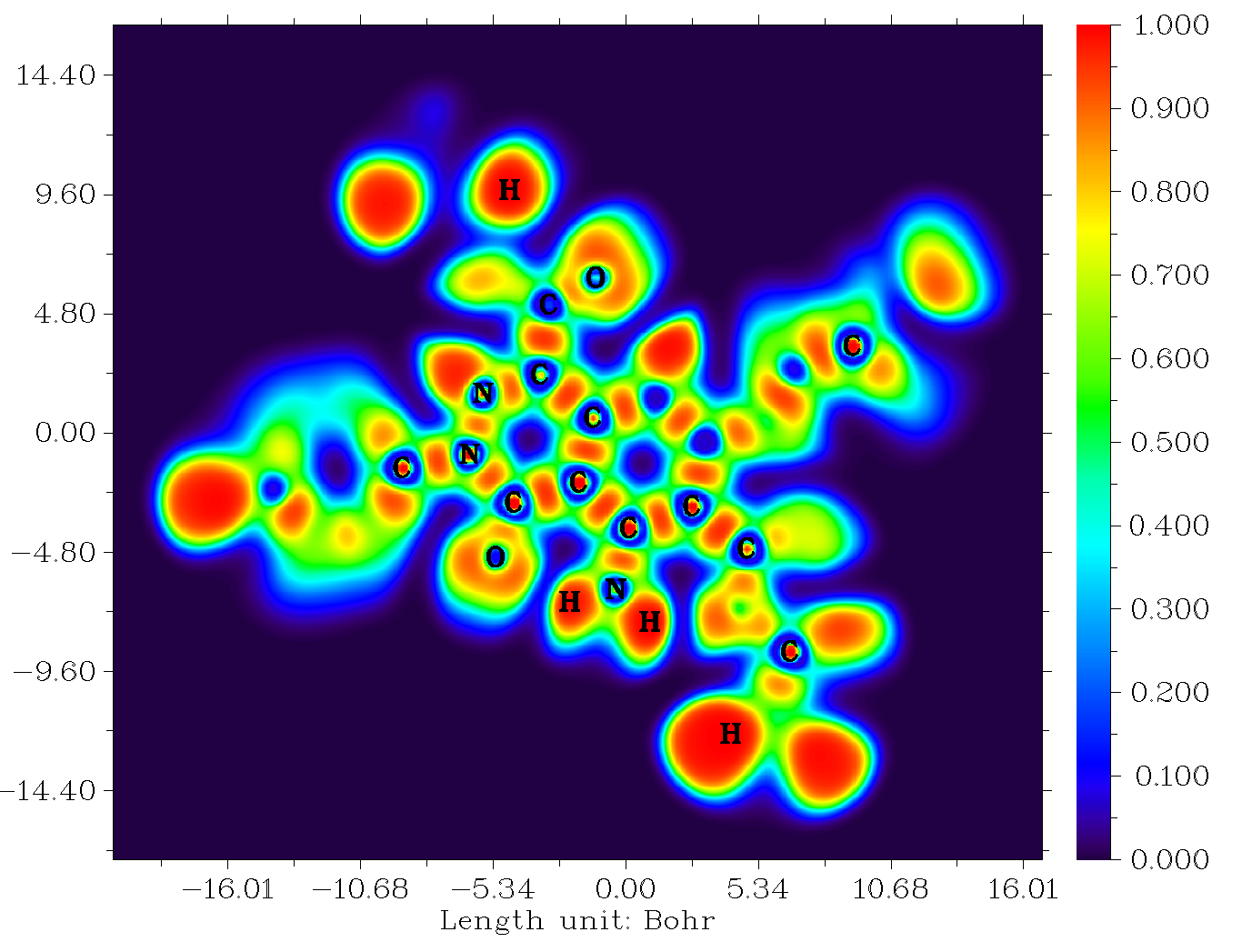


**(a)** **(b)**

**Figure 13.** Electron localization function (ELF) colored map of **3g** (**a**), and **3j** (**b**)**,** in XY plane.

- 1. Localized Orbital Locator (LOL)

The Localized Orbital Locator (LOL), as implemented within contemporary molecular modeling protocols, quantifies the degree of electron localization and distinguishes regions of strong covalent bonding and lone-pair concentration from more delocalized electron density, thereby providing a detailed picture of bonding characteristics and electronic distribution within conjugated and heteroatom-rich systems like **3g** and **3j**.^9^ LOL is a real-space electron-localization index derived from the kinetic energy density and orbital gradients. It provides a visual representation of regions where electron pairs are strongly localized, functioning similarly to the Electron Localization Function (ELF) but often offering sharper contrast between different bonding environments. High LOL values (yellow–red regions) indicate reduced electron mobility and strong localization, typically associated with covalent bonding, lone pairs, or π-electron clouds, while low LOL values (blue–purple regions) indicate delocalized or diffuse electron density. Thus, LOL contour maps are widely used to visualize bond strength, aromaticity, π-delocalization, heteroatom lone pairs, and intramolecular electronic communication.

In the LOL map of XY plane in **3g** (Figure 14a), pronounced regions of high localization (yellow–red intensity) appear along the conjugated carbon skeleton, reflecting significant π-electron density and strong covalent C–C bonding across the aromatic core. The benzene-like ring system displays uniform electron-localization maxima, confirming its aromatic stabilization and delocalized π-cloud. The substituent groups attached to the core show characteristic localization patterns: the oxygen atoms exhibit distinct localized regions corresponding to lone pairs, while the heteroatom-containing branches display slightly asymmetric LOL distributions due to differences in electronegativity and bond polarity. Notably, the terminal functional groups show concentrated electron-density pockets, indicating strong localization around more electronegative atoms, O atoms. Overall, compound **3g** demonstrates a continuous delocalized pathway across its conjugated system, supporting efficient intramolecular charge transfer capabilities. The smoother gradients and more uniform red-green areas indicate that electronic communication is distributed over the molecular framework.

The LOL contour map of XY plane in **3j** (Figure 14b) reveals a more complex electron-localization pattern compared to 3G due to the presence of additional heteroatoms (N and O) integrated within the conjugated framework. The aromatic and fused ring regions show strong localization (yellow–red), confirming stable π-delocalization, consistent with enhanced resonance stabilization. The nitrogen atoms embedded within the ring system display well-defined localized pockets corresponding to lone pairs, and these regions interact with adjacent π-systems, indicating participation in resonance. The oxygen substituents also show strong localization around their lone pairs, typically visualized as intense red spots. In **3j**, the electronic density is more polarized, particularly around N and O centers, generating sharper contrasts between localized and delocalized regions. The peripheral substituents show distinct localization associated with C–H bonds, while some extended branches show slightly diffuse green–blue regions, suggesting partial delocalization. Compared with **3g**, compound **3j** exhibits a richer network of localized lone-pair regions coupled with π-delocalized zones, indicating that heteroatom inclusion enhances the density distribution and potentially contributes to stronger electronic effects such as donor–acceptor interactions.


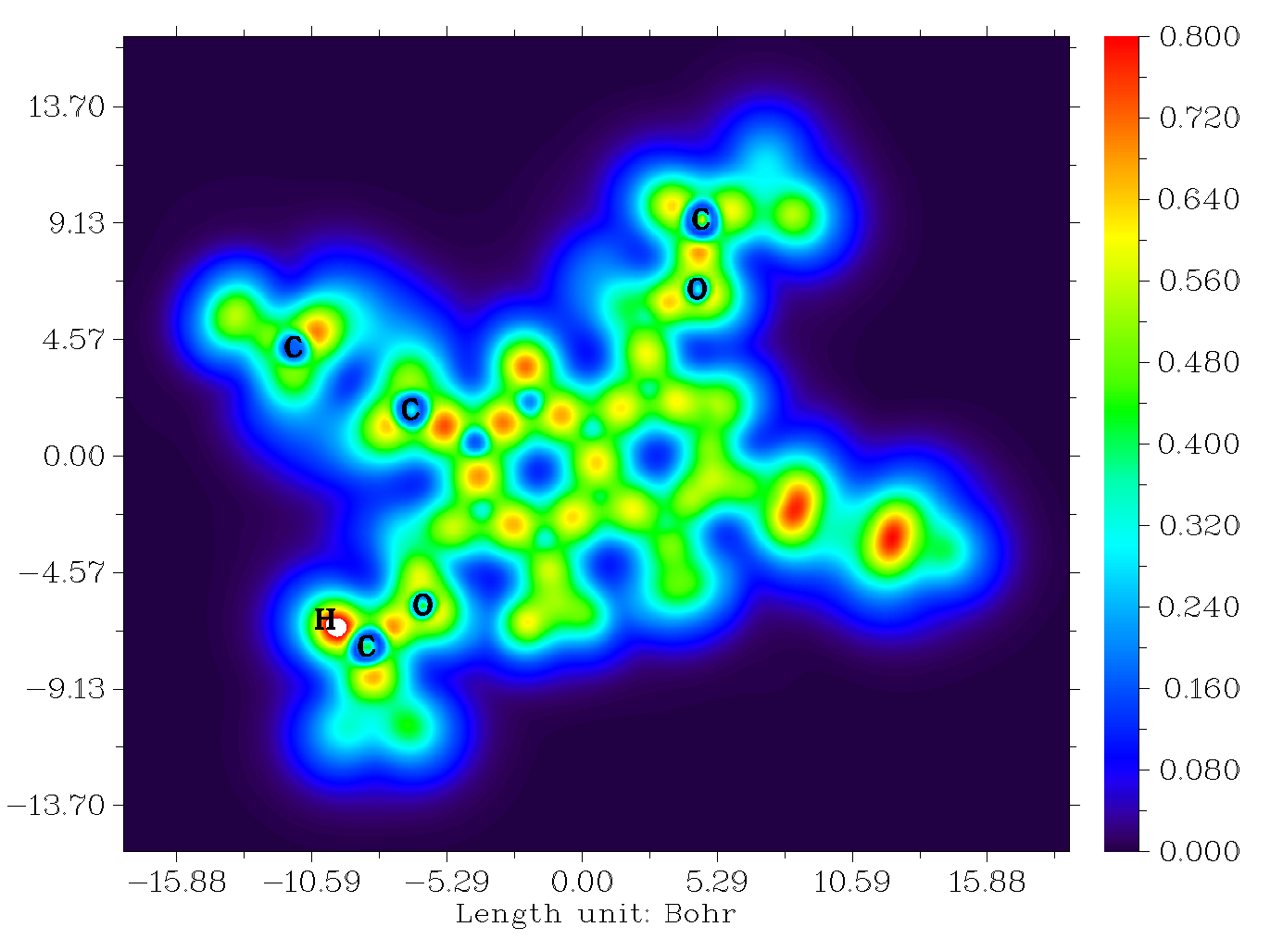

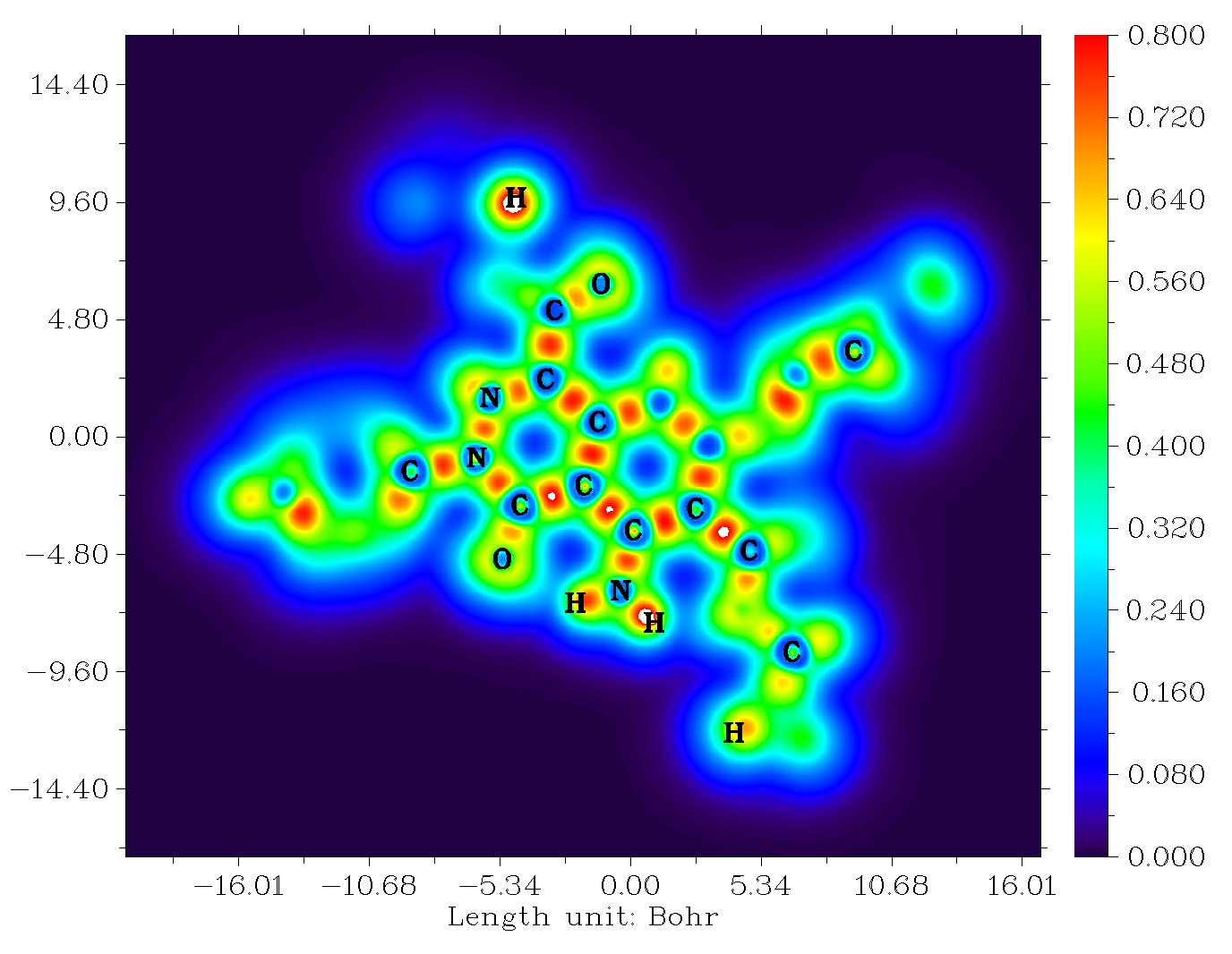


1. (**b**)

**Figure 14.** Localized Orbital Locator (LOL) colored map of **3g** (**a**), and **3j** (**b**), in XY plane.

- 1. Molecular electrostatic potential (MEP)

The molecular electrostatic potential (MEP) 3D map serves as a valuable tool for visualizing the distribution of electrostatic potential across a molecule, thereby highlighting regions of varying electron density. This topological descriptor is instrumental in elucidating non-covalent interactions, molecular recognition, and reactivity, as electrostatic forces predominantly govern long-range intermolecular interactions.^10^ In the present study, the MEP surfaces of the target compounds were analyzed using a color-coded scheme—red, orange, yellow, green, and blue-corresponding to regions of decreasing electrostatic potential. Electron-rich (nucleophilic) regions are indicated by red zones, while electron-deficient (electrophilic) regions appear in blue. The analysis revealed that oxygen atoms exhibit the highest negative electrostatic potential, indicating a strong nucleophilic character. As shown in Figures 15 and 16, the electron-rich parts in both compounds contributed to the carbonyl (C=O) group, while NO_2_ is the best electron-rich group present in **3j** compound. F and Cl atoms also exhibit some electronic-rich character in **3g,** whereas hydrogen atoms of amino and ethyl groups represent potential electrophilic sites. The phenyl rings exhibited relatively neutral potential, with a moderate π-electron density reflected by a yellow color. This distribution suggests a propensity for nucleophilic attack by oxygen atoms on electrophilic centers, particularly hydrogen atoms of primary and secondary amines, thereby facilitating electron transfer within the molecular framework.


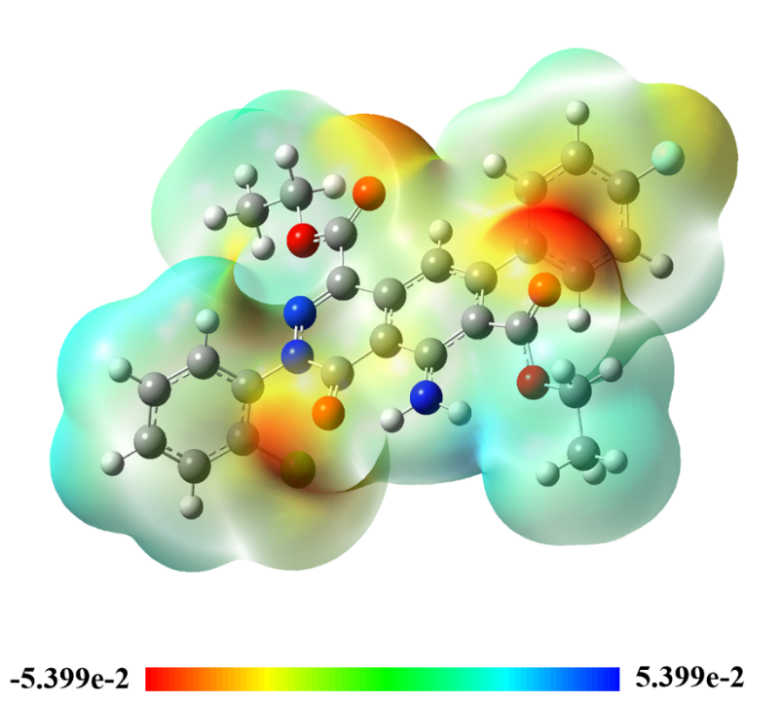


**Figure 15.** 3D-colored map of molecular electrostatic potential of **3g**.


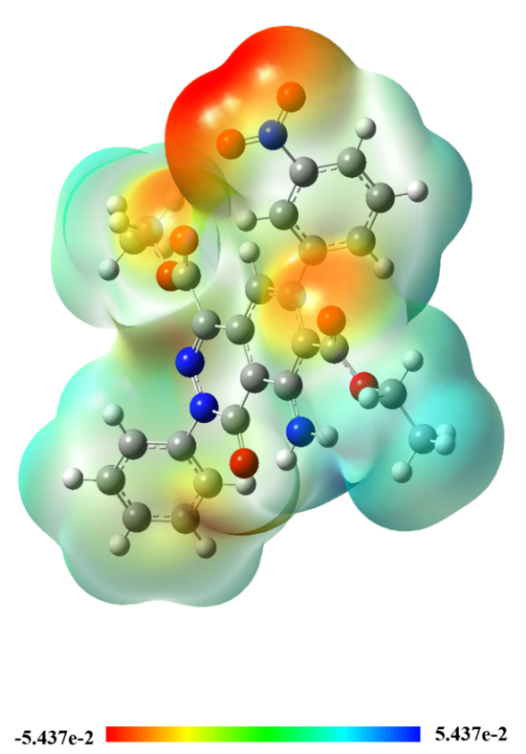


**Figure 16.** 3D-colored map of molecular electrostatic potential of **3j**.

- 1. Reduced density gradient/non-covalent-interactions (RDG/NCI)

Noncovalent interactions (NCIs) within the studied molecules were characterized using the reduced density gradient (RDG) approach, which enables the visualization of weak intermolecular forces based on the electron density and its derivatives. Color-mapped RDG *iso*-surfaces were employed to distinguish various types of NCIs.^11^ As shown in Figures 17 and 18, hydrogen bond interactions are represented in blue color, van der Waals (vdW) interactions are represented in green, while steric (repulsive) interactions appear in red. The quantity sign(λ₂)ρ, derived from the product of the electron density (ρ) and the sign of the second eigenvalue (λ₂) of the Hessian matrix, was used to evaluate the nature and intensity of these interactions, particularly hydrogen bonding (H-bond). In the present analysis, prominent H-bond interactions were identified in the molecular regions between hydrogen atoms of the amino group and the adjacent oxygen atoms.

Also, significant vdW interactions are present in the cages of aromatic rings and acetate groups in both studied heterocyclic compounds. This type indicates a weak attractive force stabilizing the molecular conformation. Additional electrostatic interactions were observed between the two phenyl rings, suggesting π–π stacking or weak dispersion interactions. Steric repulsion, predominantly located inside the phenyl rings, is a result of spatial crowding within the constrained six-membered aromatic systems. However, such destabilizing interactions may be partially mitigated by favorable, strong H-bonds and electrostatic attractions elsewhere in the studied molecules, balancing the overall intermolecular interaction landscape.


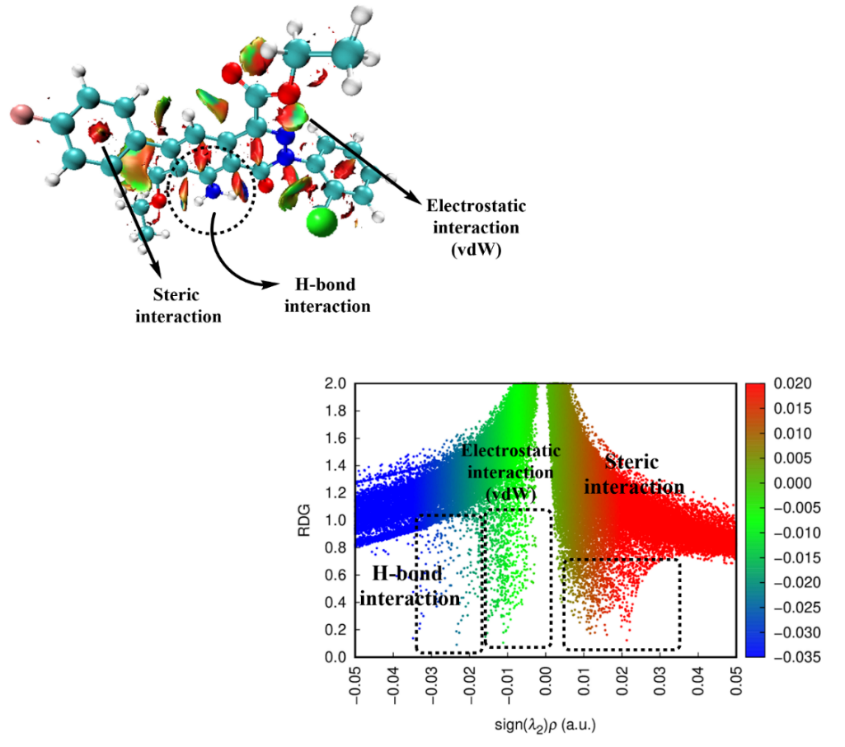


**Figure 17.** 3D-NCI map and RDG plot of the designed compound **3g**.


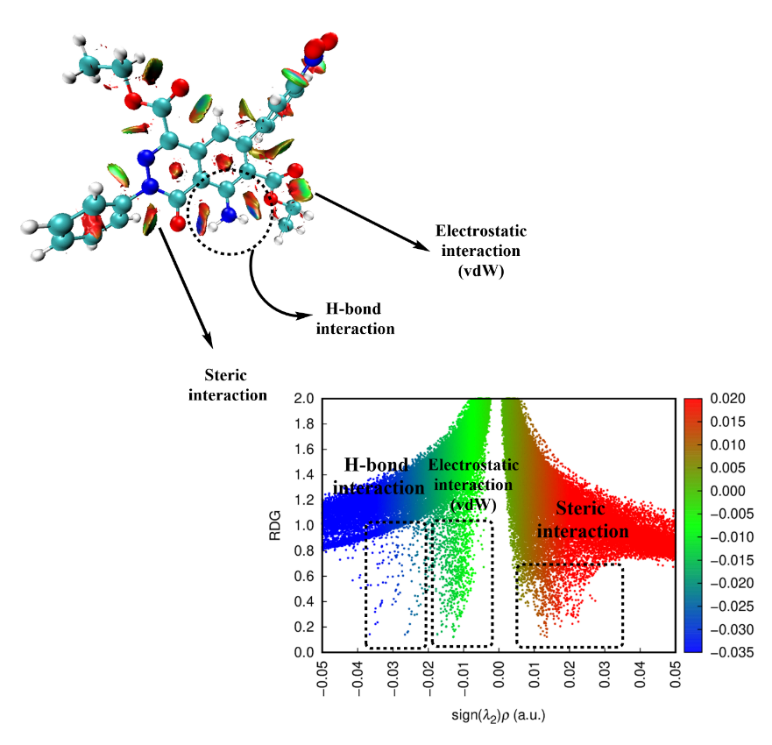


**Figure 18.** 3D-NCI map and RDG plot of the designed compound **3j**.

2. Spectral Data

**
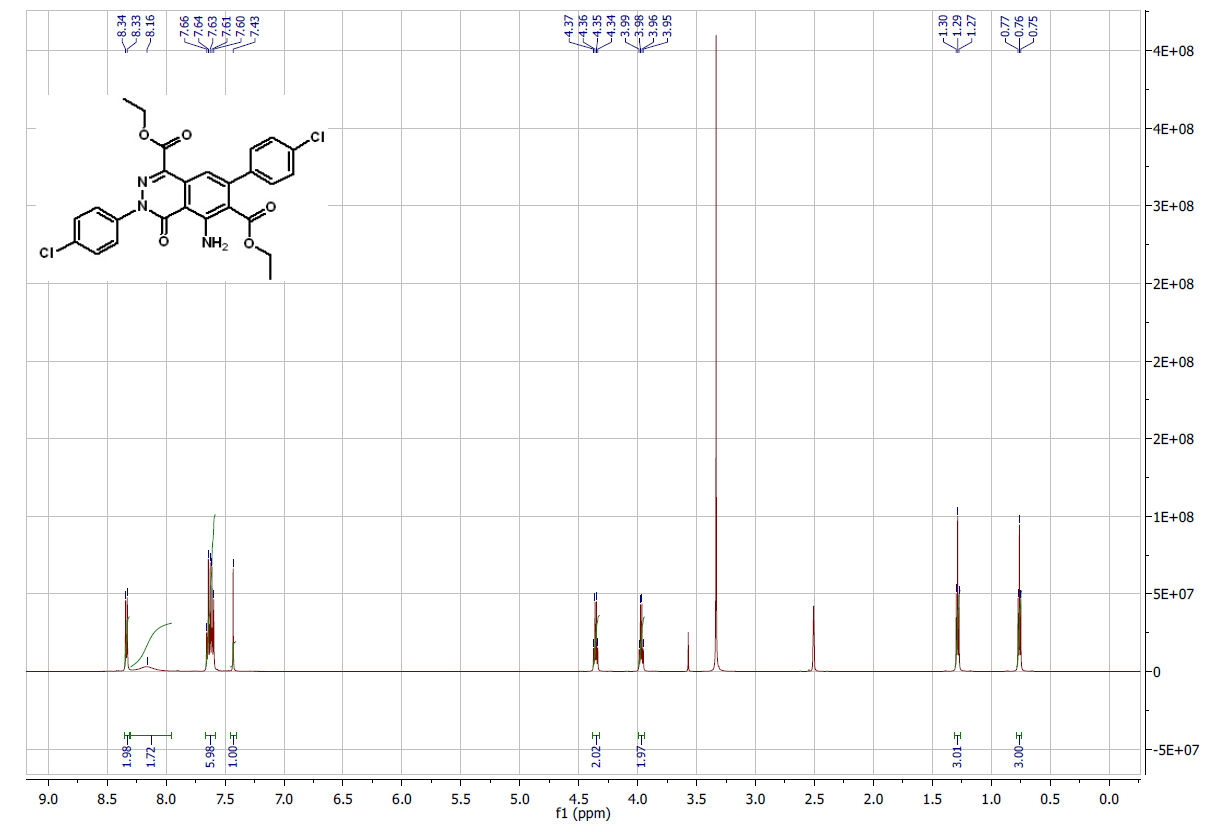
**

**Figure 19**. ^1^H NMR spectrum of compound **3a**.

**
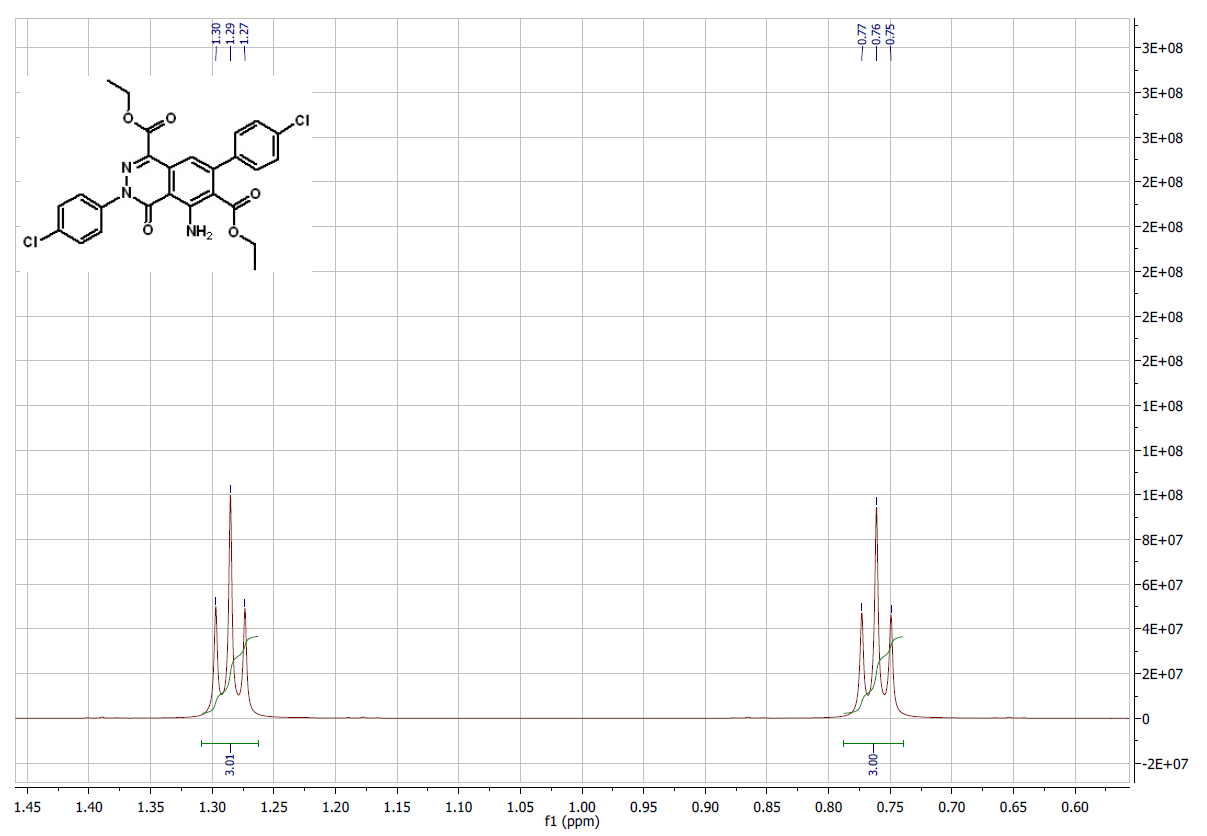
**

**
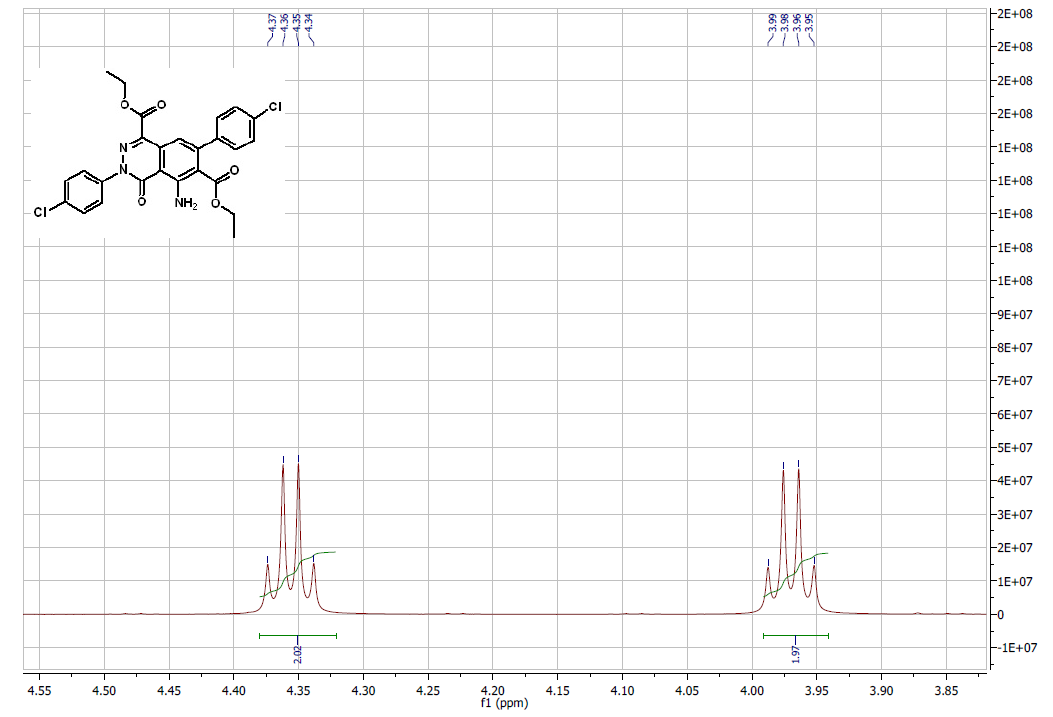
**

**Figure 20**. Expanded ^1^H NMR of compound **3a**.

**
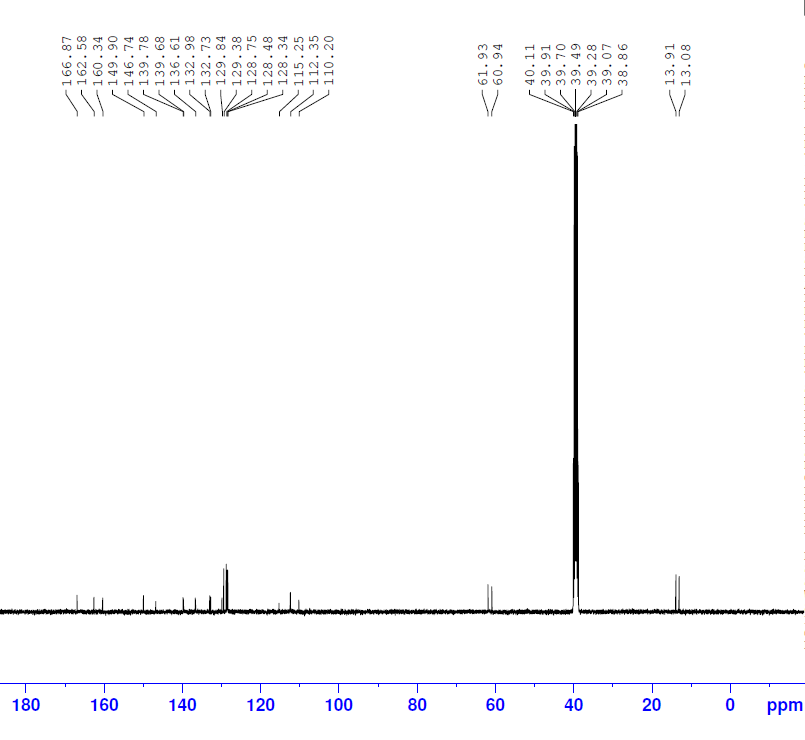
**

**Figure 21**. ^13^C NMR spectrum of compound **3a**.

**Figure 22**. Mass spectrum of compound **3a**.

**Figure 23**. IR spectrum of compound **3a**.

**
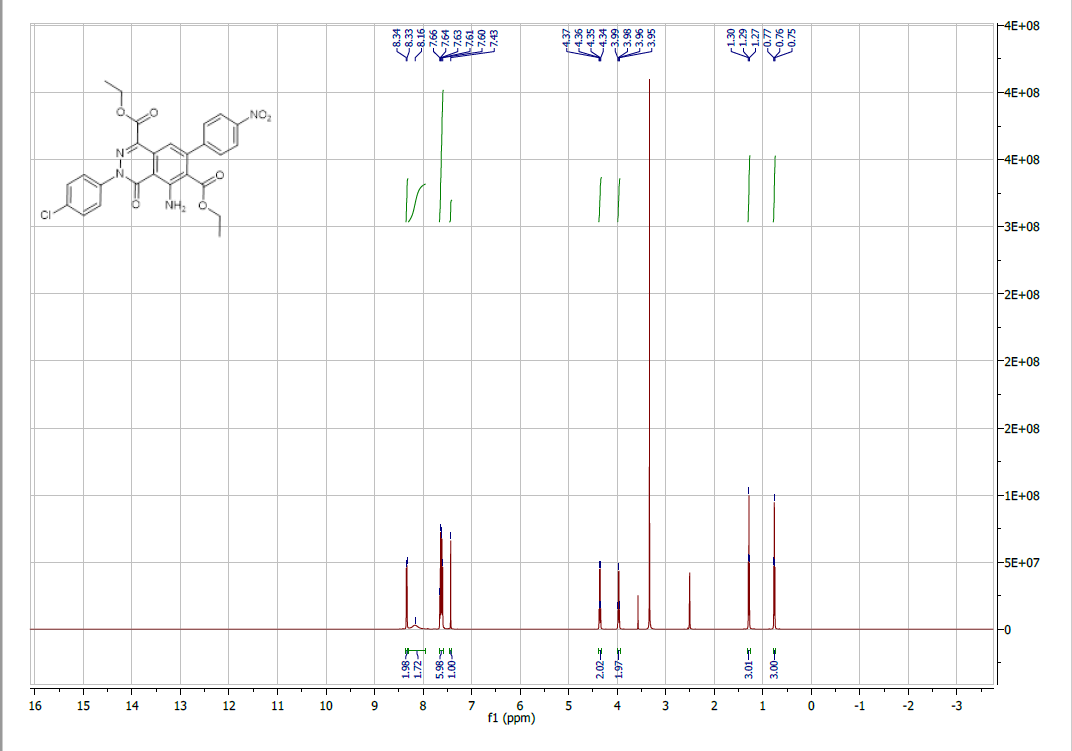
**

**Figure 24**. ^1^H NMR spectrum of compound **3b**.


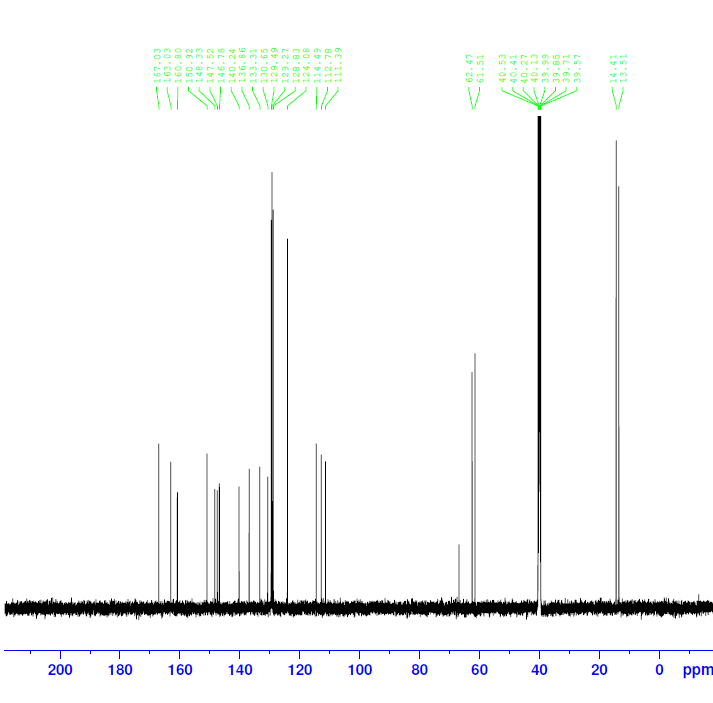


**Figure 25**. ^13^C NMR spectrum of compound **3b**.

**Figure 26**. Mass spectrum of compound **3b**.

**Figure 27.** IR spectrum of compound **3b**.

**
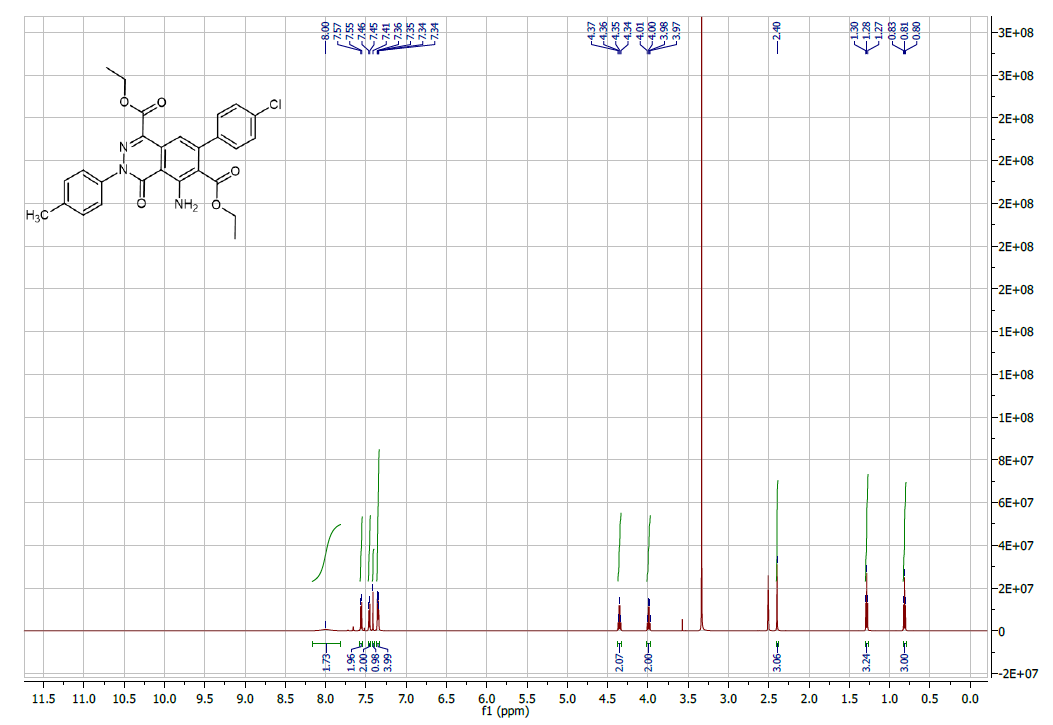
**

**Figure 28**. ^1^H NMR spectrum of compound **3c**.

**
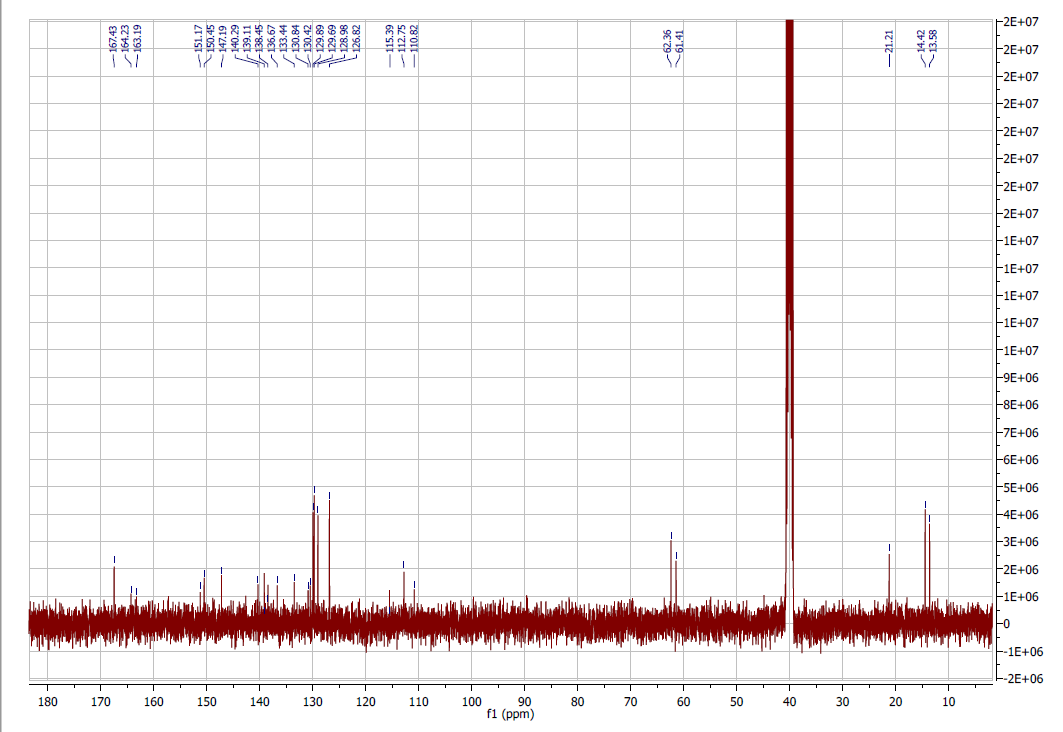
**

**Figure 29**. ^13^C NMR spectrum of compound **3c**.

**Figure 30**. Mass spectrum of compound **3c**.


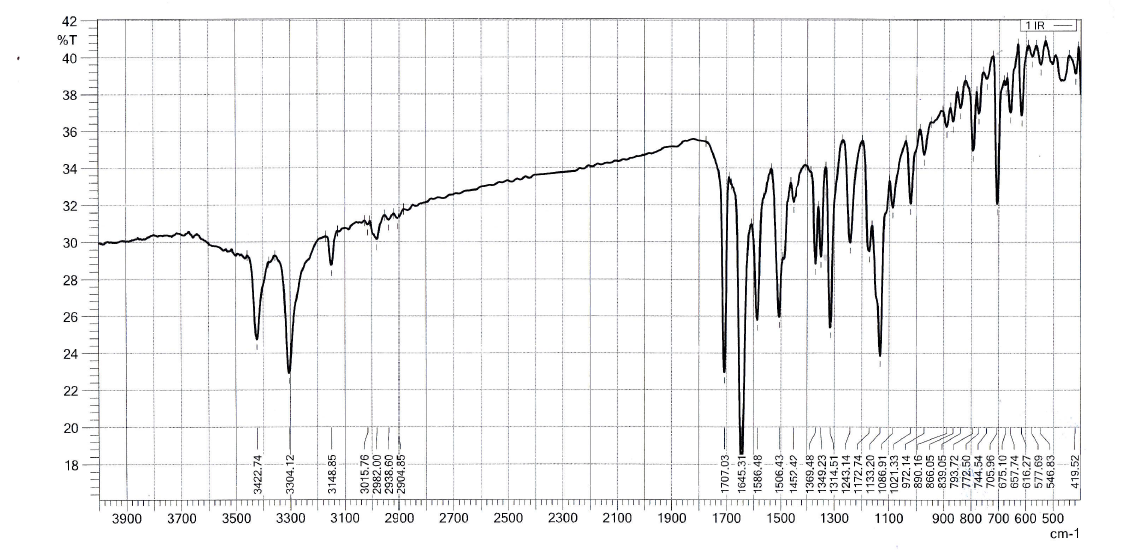


**Figure 31**. IR spectrum of compound **3c**.

**Figure 32**. ^1^H NMR spectrum of compound **3d**.


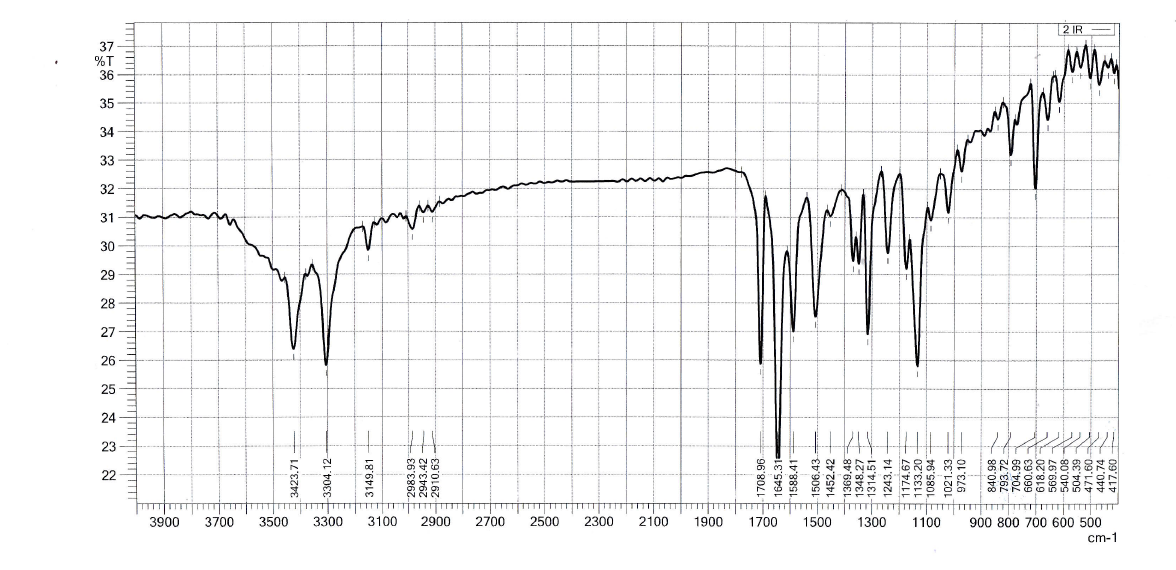


**Figure 33**. IR spectrum of compound **3d**.

**
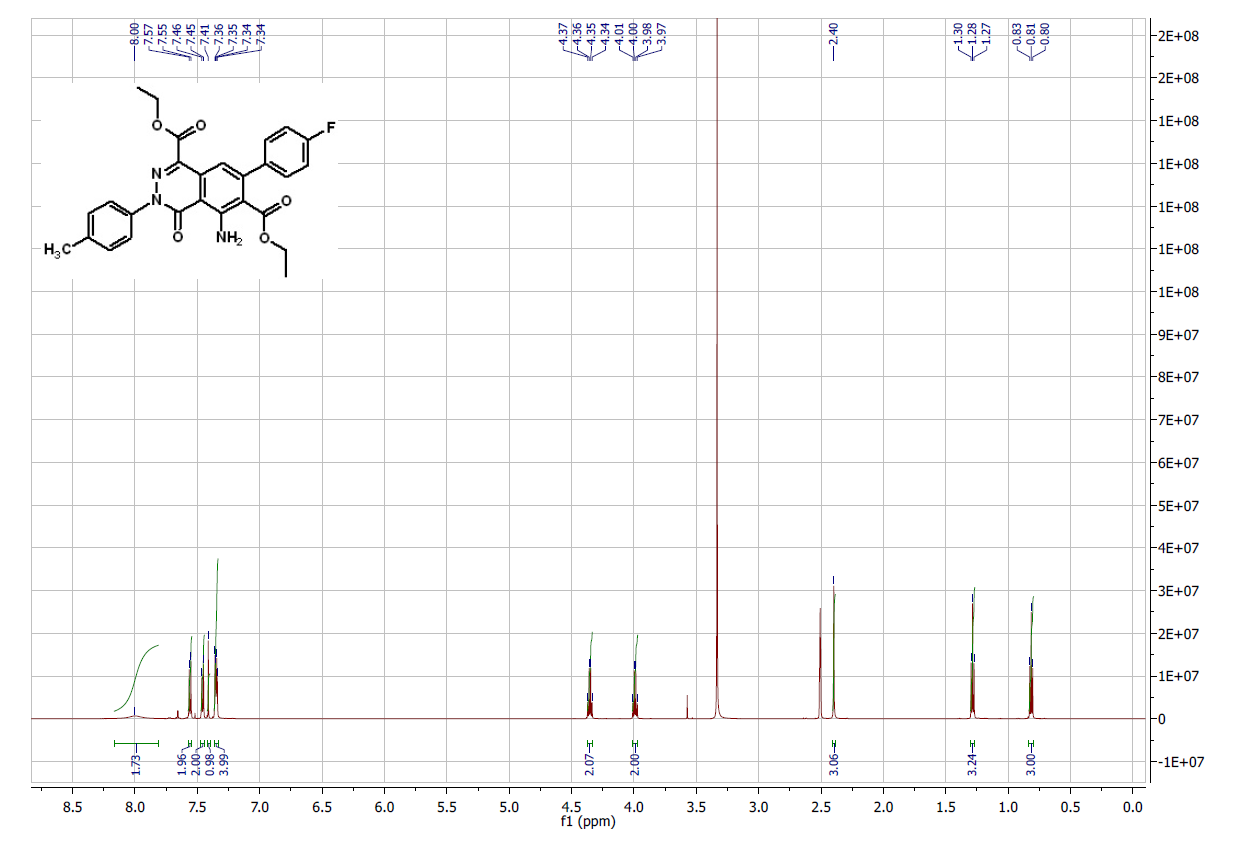
**

**Figure 34**. ^1^H NMR spectrum of compound **3e**.

**
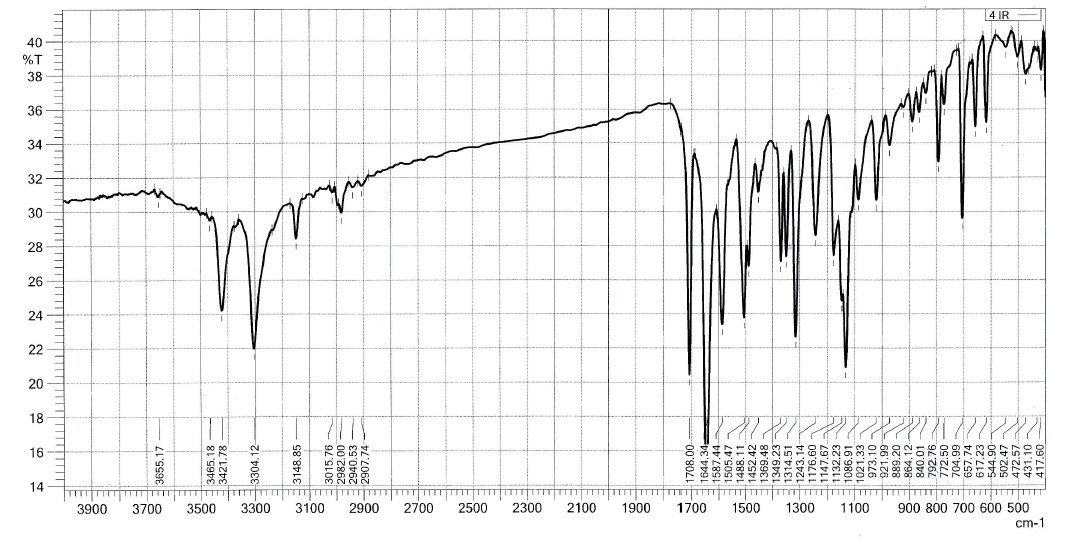
**

**Figure 35**. IR spectrum of compound **3e**.


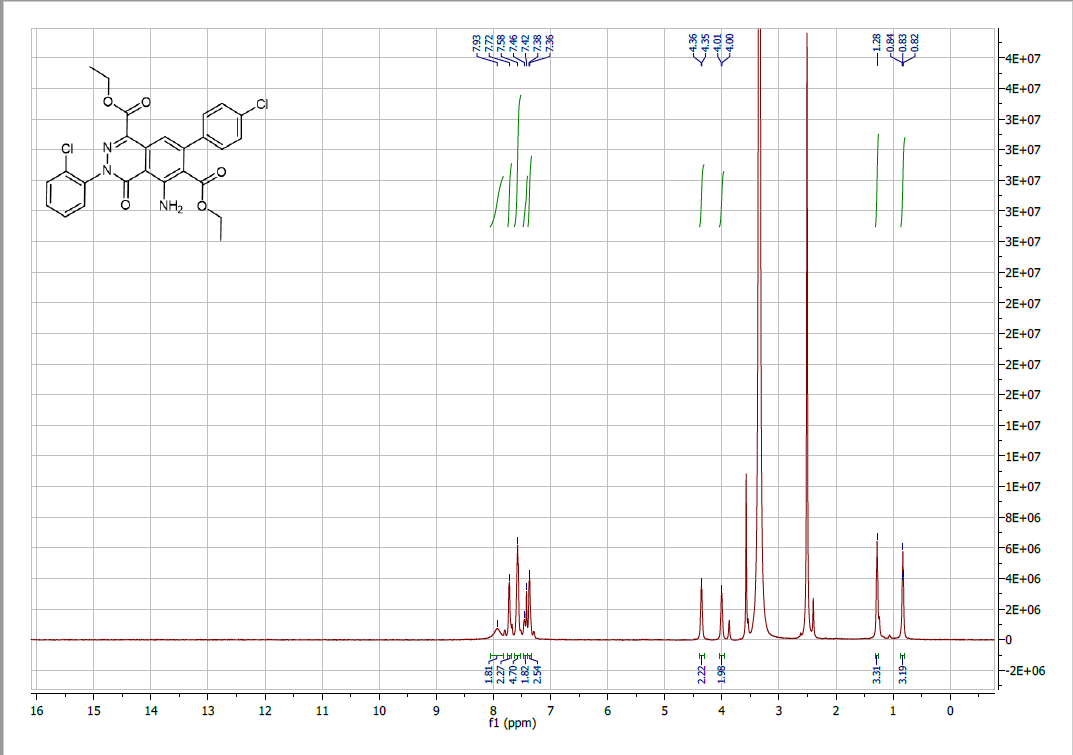


**Figure 36**. ^1^H NMR spectrum of compound **3f**.

**
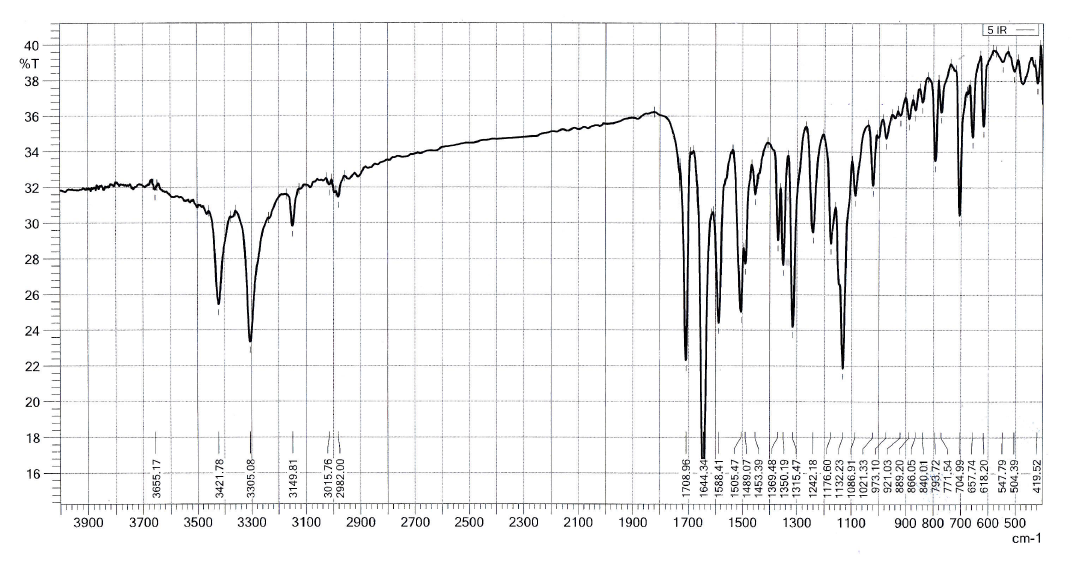
**

**Figure 37**. IR spectrum of compound **3f**.


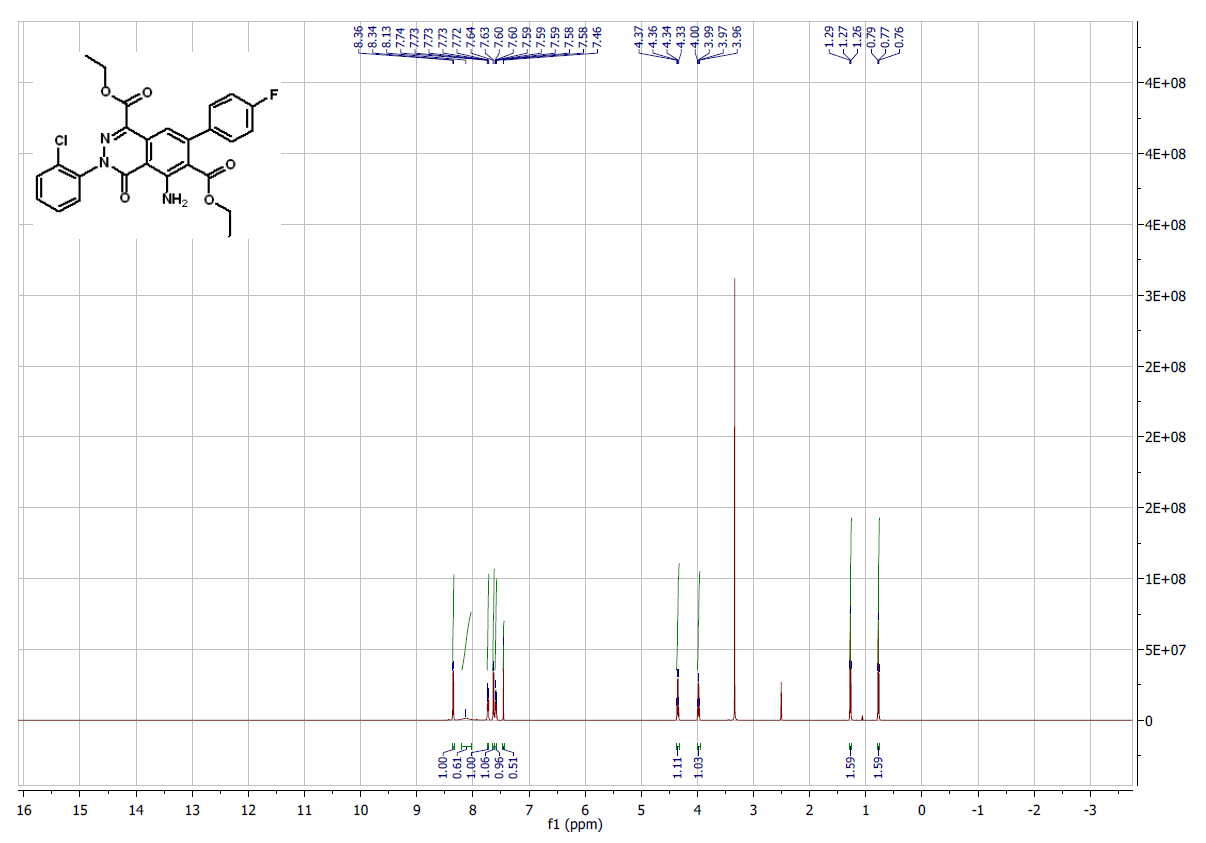


**Figure 38.** ^1^H NMR spectrum of compound **3g**.


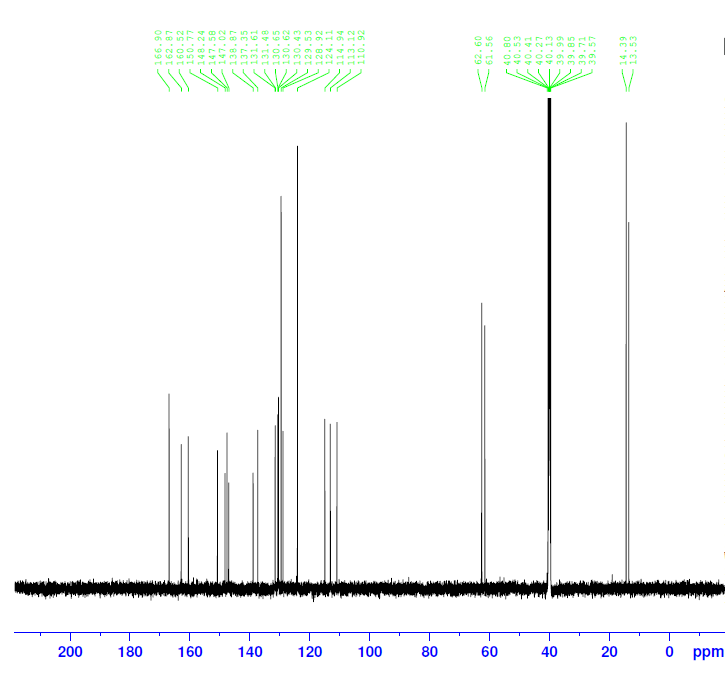


**Figure 39**. ^13^C NMR spectrum of compound **3g**.

**Figure 40**. IR spectrum of compound **3g**.


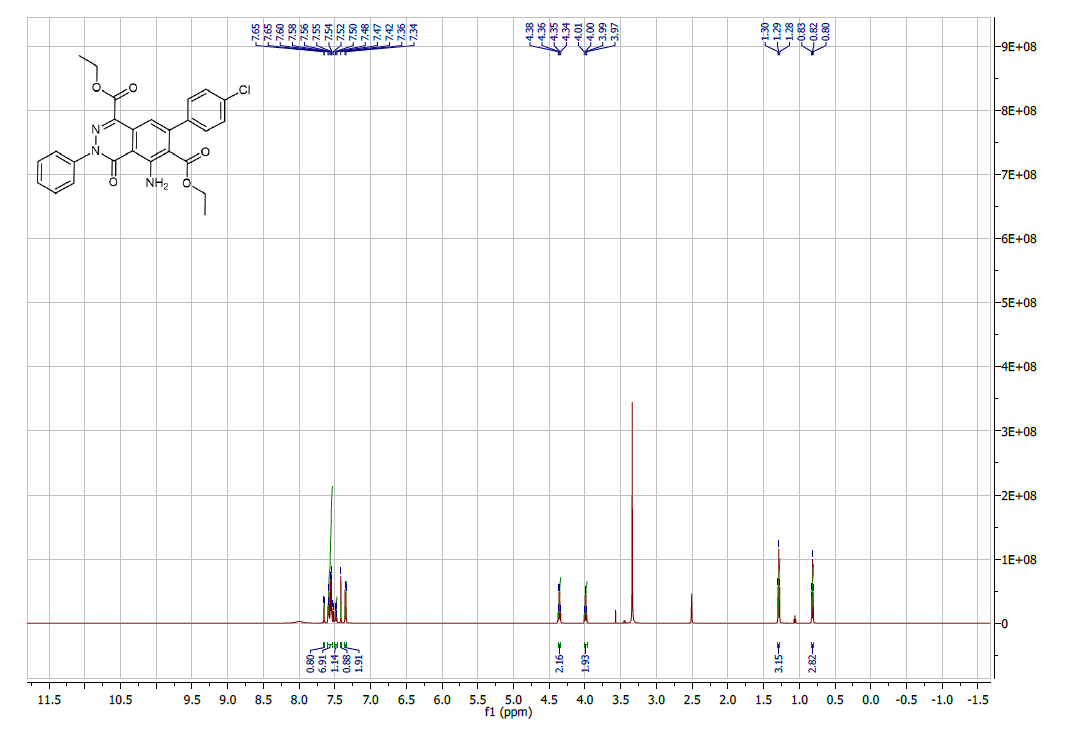


**Figure 41**. ^1^H NMR spectrum of compound **3h**.

**Figure 42**. IR spectrum of compound **3h**.


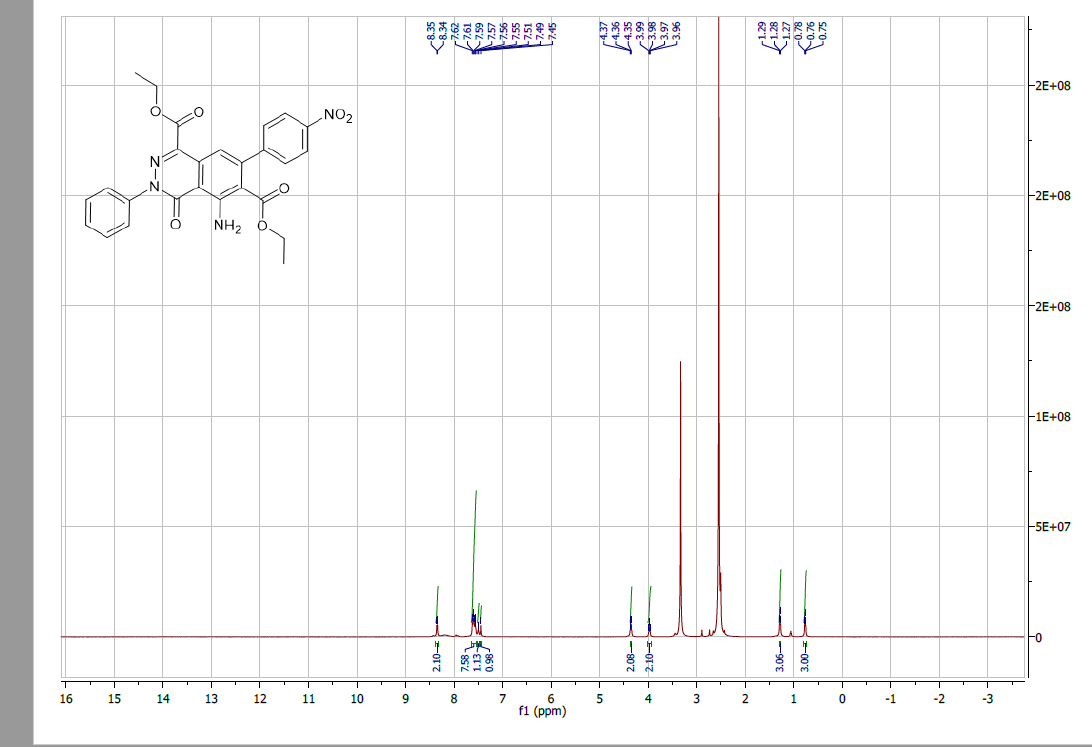


**Figure 43**. ^1^H NMR spectrum of compound **3i**.


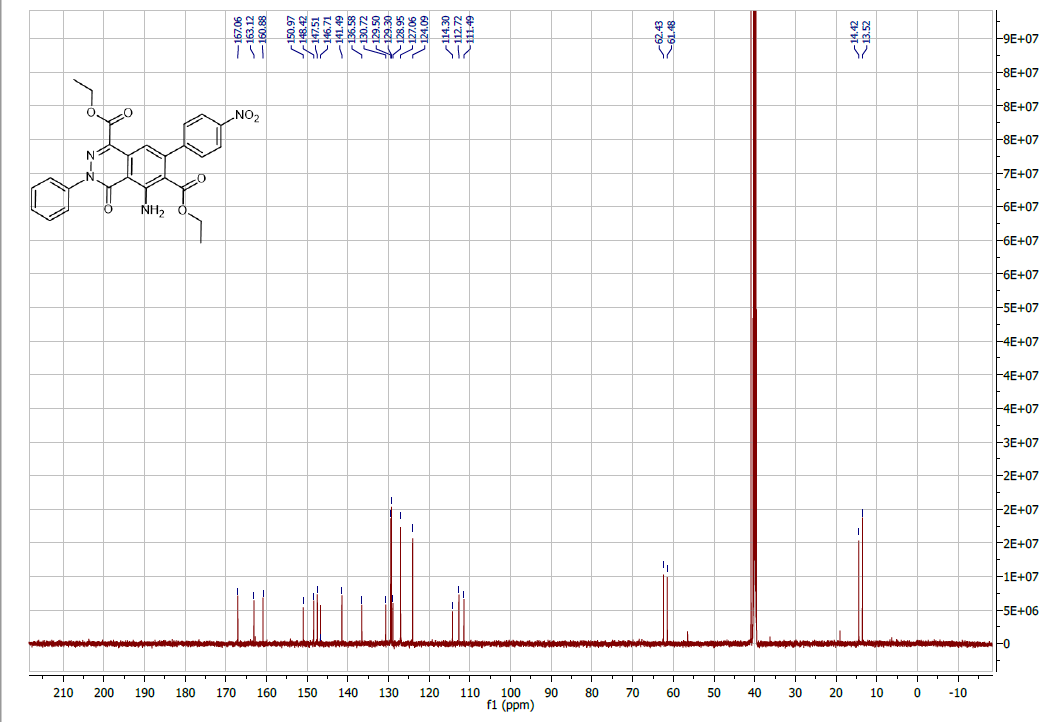


**Figure 44**. ^13^C NMR spectrum of compound **3i**.


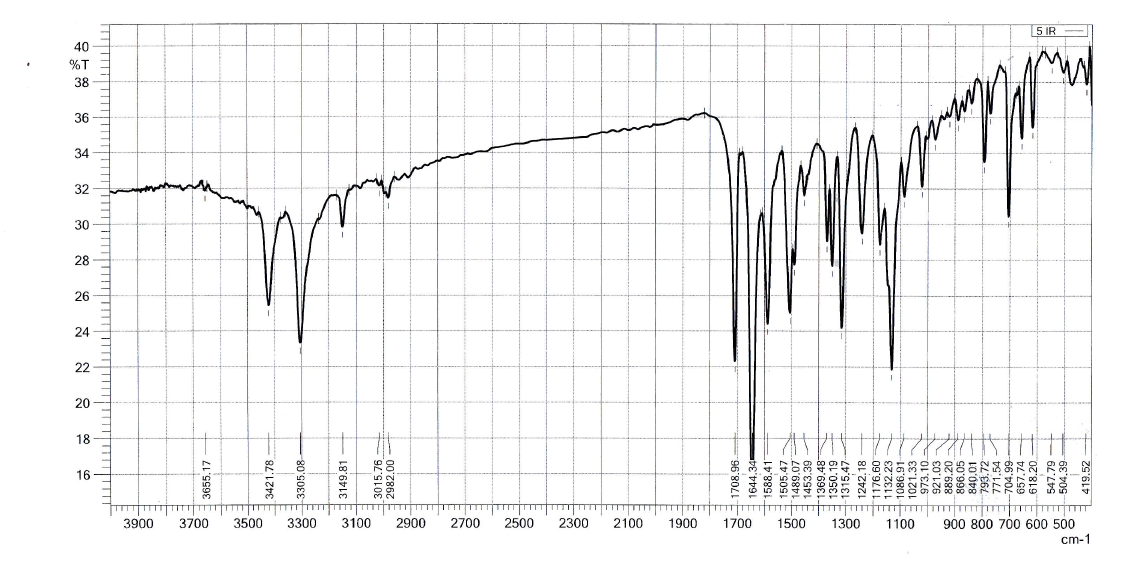


**Figure 45**. IR spectrum of compound **3i**.

**Figure 46**. ^1^H NMR spectrum of compound **3j**.

**Figure 47**. IR spectrum of compound **3j**.

3. References

1. Parr, R. G. & Weitao, Y. *Density-Functional Theory of Atoms and Molecules*. (Oxford University Press, 1989).

2. Fayed, T. A., Gaber, M., Abu El‐Reash, G. M. & El‐Gamil, M. M. Structural, DFT/B3LYP and molecular docking studies of binuclear thiosemicarbazide Copper (II) complexes and their biological investigations. *Appl. Organomet. Chem.* **34**, 1–20 (2020).

3. Eletmany, M. R. *et al.* Novel arylazo nicotinate derivatives as effective antibacterial agents: Green synthesis, molecular modeling, and structure-activity relationship studies. *J. Saudi Chem. Soc.* **27**, 101647 (2023).

4. Abdellah, I. M., Zaky, O. S. & Eletmany, M. R. Visible light photoredox catalysis for the synthesis of new chromophores as co-sensitizers with benchmark N719 for highly efficient DSSCs. *Opt. Mater.* **145**, 114454 (2023).

5. Yu, J., Su, N. Q. & Yang, W. Describing Chemical Reactivity with Frontier Molecular Orbitalets. *JACS Au* **2**, 1383–1394 (2022).

6. Elbadawy, H. A., Ali, A. E.-D., Elkashef, A. A., Foro, S. & El‐Sayed, D. S. Zinc(II)‐facilitated nucleophilic addition on N‐(4‐chlorophenyl) carbon hydrazonoyl dicyanide and hybrid complex formation: X‐ray, spectral characteristics, DFT, molecular docking, and biological studies. *Appl. Organomet. Chem.* **36**, e6793 (2022).

7. Khaoua, O. *et al.* Synthesis, in vitro antimicrobial activity, theoretical DFT-based reactivity investigations, NCI-RDG, NLO, EFL, LOL, AIM analyses, molecular docking, and dynamic simulations of novel 2-(Hydroxy(tetrazolo[1,5-a]quinolin-4-yl)methyl)acrylonitrile derivative. *J. Mol. Struct.* **1349**, 143849 (2026).

8. Boutamdja, M., Khaoua, O., Benbellat, N., Laib, S. & Bendaikha, T. Synthesis, characterization, NCI-RDG, EFL, LOL, AIM, TDDFT/ fluorescence, and eco-toxicity analyses of a polymerizable photo stabilizer based on 2-hydroxybenzophenone. *J. Mol. Struct.* **1349**, 143782 (2026).

9. Khaoua, O. Reactivity, bioactivity, and antileishmanial activity of dihydrosyrindine and syringine: Modelling, cytotoxicity, molecular docking, molecular dynamics, and MM-GBSA analyses. *J. Mol. Graph. Model.* **142**, 109183 (2026).

10. Saleh, G., Gatti, C., Lo Presti, L. & Contreras‐García, J. Revealing Non‐covalent Interactions in Molecular Crystals through Their Experimental Electron Densities. *Chem. Eur. J.* **18**, 15523–15536 (2012).

11. Emara, M. M. *et al.* Electronic and structural perturbations of microporous ZIF-67 nanoparticles and Cr(VI) molecule during adsorptive water decontamination unveiled by experimental and quantum computational investigations. *J. Mol. Liq.* **390**, 123042 (2023).
